# Infrasound exposure is linked to aversive responding, negative appraisal, and elevated salivary cortisol in humans

**DOI:** 10.3389/fnbeh.2026.1729876

**Published:** 2026-04-27

**Authors:** Kale R. Scatterty, Dawson VonStein, Lisa B. Prichard, Brian C. Franczak, Trevor J. Hamilton, Rodney M. Schmaltz

**Affiliations:** 1 Department of Psychology, MacEwan University, Edmonton, AB, Canada; 2 Neuroscience and Mental Health Institute, University of Alberta, Edmonton, AB, Canada; 3 Department of Biological Sciences, MacEwan University, Edmonton, AB, Canada; 4 Department of Mathematics and Statistics, MacEwan University, Edmonton, AB, Canada

**Keywords:** aversion, behaviour, cortisol, ELISA, infrasound, perception, psychoacoustics, stress

## Abstract

**Introduction:**

Infrasound describes sound wave frequencies below 20 Hz, which are typically imperceptible to humans. Some animals perceive and demonstrate aversion to infrasound, raising concerns about its potential adverse effects as an anthropogenic pollutant. Recent research suggests humans may also respond to infrasound, despite being below the conventional limit of human hearing. This study explored the non-auditory impact of infrasound on human mood and stress responding.

**Methods:**

Participants (*n* = 36) were exposed to calming or unsettling music with infrasound (~18 Hz) present or absent in a 2 × 2 between-subjects design (*calming* vs. *unsettling*, *infrasound on vs. off*). Self-report measures were collected immediately post-exposure, and saliva was collected immediately pre-exposure and 20 min post-onset for cortisol assay.

**Results:**

Participants did not detect infrasound above chance (*p* = 0.241). Infrasound was associated with elevated salivary cortisol (*p* = 0.022, *r_rb_* = 0.390) and higher self-reported irritability (*p* = 0.049, *η^2^* = 0.096), disinterest (*p* = 0.044, *η^2^* = 0.121; *p* = 0.047, *η^2^* = 0.118), and sadness appraisal (*p* = 0.002, *η^2^* = 0.253) across both music conditions, with no expectancy effects. Interest, irritability, sadness appraisal, and cortisol were also identified as important predictors of infrasound exposure via random-forest modeling.

**Discussion:**

Without auditory detection nor expectancy effects, infrasound exposure was linked to elevated cortisol and more negative affective self-reporting. These findings align with previous animal studies and suggest infrasound may be aversive to humans, acting as a potential environmental irritant and contributing to more negative subjective experience.

## 1 Introduction

Infrasound can be defined acoustically as soundwaves with an upper frequency limit below 20 Hz (Bedard and Georges, 2000). Infrasound has been postulated to cause aversion and feelings of fear in supposedly haunted locations and to contribute to anxiety, distress, and reduced well-being in the vicinity of energy infrastructures such as wind turbines (Boczar et al., 2022; Scatterty et al., 2023; Salt and Hullar, 2010; Persinger, 2013; Burt, 1996; Chaban et al., 2021; Leventhall, 2007; Friedrich et al., 2023; Tandy and Lawrence, 1998). Infrasound also occurs naturally, generated, for example, by tectonic or volcanic activity (Hamama and Yamamoto, 2021; Watson et al., 2022; Marchetti et al., 2019), convective storms (Sindelarova et al., 2015), and air-water interactions such as during upstream water discharges (Che et al., 2023). Infrasound is also, however, prevalent in urban areas near ventilation systems, air conditioning, low-rumbling pipes, traffic and building power, heating, mechanical systems (Persinger, 2013; Burt, 1996; Butkus and Vasiliauskas, 2013; Grafkina et al., 2019; McComas et al., 2019; Wynn and Dugick, 2023; Nowicki et al., 2014). Exploratory field recordings also detected low-frequency acoustic energy in the infrasound range from similar urban sources in Edmonton (AB, Canada) as well as during musical performances (see Supplemental materials).

Animal models suggest that infrasound may alter affective state and stress responses (Scatterty et al., 2023; Salt and Hullar, 2010; Karlsen, 1992a; Karlsen, 1992b; Enger et al., 1993; Sand and Karlsen, 1986; Sand and Karlsen, 2000; Bui et al., 2013; Karlsen et al., 2004; Sand et al., 2001), however, it is unknown whether this also applies to humans, as direct behavioral, physiological, and anatomical comparisons in the context of infrasound exposure are limited and cannot yet be reliably made. Some studies indicate that infrasound can negatively affect human sleep and potentially induce feelings of fatigue, nausea, or anxiety (Persinger, 2013) as well as elevated feelings of annoyance and discomfort (Møller, 1984). However, research on the impact of infrasound on humans has been conflicting (Leventhall, 2007; Maijala et al., 2021; Mühlhans, 2017). While some investigations report little significant effect of infrasound exposure on human physiological or psychological measures (Leventhall, 2007) others have documented adverse reactions, including discomfort, anxiety, and sleep disturbances (Persinger, 2013; Møller, 1984). A likely contributor to these mixed findings is that studies tend to rely heavily on observational designs and self-report outcomes, with limited experimental control over infrasonic exposure and inconsistent reporting or validation of stimulus characteristics. Additionally, relatively few controlled studies pair verified infrasound exposure and its effects on self-reporting with established physiological markers of affective or stress-related change. Currently these factors make it difficult to determine whether infrasound can *causally* modulate affect and stress at both psychological and physiological levels under controlled conditions.

Experimental paradigms that pair verified infrasonic exposure with both self-report and physiological measures provide a direct way to test whether infrasound influences affect and stress responses in humans. In the present study, we paired the presence or absence of infrasound (~18 Hz, 75–78 dB) with music stimuli designed to be calming or unsettling and examined both self-reported affect and pre-post salivary cortisol as convergent indicators of affective and stress-related response. We hypothesized that infrasound exposure would shift self-reported affect negatively and increase cortisol relative to no-infrasound conditions, independent of music type. We further expected that any effects would be detectable independently of participant awareness of the presence of infrasound in the room. Accordingly, we tested whether infrasound exposure heightens negative affective responses and increases salivary cortisol relative to infrasound-off controls, and whether any such effects depend on music context within the 2 × 2 factorial design. By combining verified infrasound exposure with convergent self-report and physiological outcomes, this study provides a controlled investigation of whether infrasound can modulate human affect and stress physiology under experimentally controlled conditions.

## 2 Methods and materials

### 2.1 Participants

A convenience sample of undergraduate MacEwan University students (*n_target_ = 40, n_total_* = 36; 9 males, 27 females; *μ_Age_* = 23.471, *SEM* = 1.231, *min* = 18, *max* = 36; Edmonton, AB, Canada) was recruited from second and third-year psychology classes and granted 1% bonus credit toward their final class grade for participating in the study. Participants were randomly assigned to one of four groups: (i) *infrasound on + calming music*, (ii) *infrasound off + calming music*, (iii) *infrasound on + unsettling music*, and (iv) *infrasound off + unsettling music,* resulting in a 2×2 grouping structure. Participants were considered eligible for the study if they were adults (≥18 years) able to provide informed consent and complete the protocol and reported no health concerns that might reasonably confound auditory perception (e.g., hearing impairment). Participants who failed to follow pre-testing instructions upon enrollment (e.g., refraining from food, beverages other than water, and marijuana or tobacco products for at least 1 hour prior to testing) were also to be excluded. Since the sample size was small, effect sizes were also calculated and reported as they are independent of sample size limitations. Given the sample’s limited size and heavy skewedness toward female participants, unbiased analyses of predictive variable importance (discussed ahead) were employed to descriptively complement significance findings. This study received ethical approval from the MacEwan University Research Ethics Board. All research was performed in accordance with relevant guidelines and regulations.

All participants provided experimental informed consent after an introduction and explanation of the study. Participants were informed that they could withdraw consent at any point and none withdrew during the study period. Testing of participants took place over 10 days (July 24, 2023—August 3, 2023) during which students were allowed to sign up for a 1-h time slot between 9 a.m. and 5 p.m. each day via the online participant recruitment system. Participants were instructed to refrain from consuming food, beverages other than water, and marijuana or tobacco products for at least 1 hour prior to the testing period. No participants reported health concerns outside of psychiatric medication, such as history of hearing impairment. Each participant was only able to participate in the study once, and credit was only granted to the student after completion of the study. A script was used for welcoming, informing, and instructing participants to ensure that each participant received the same communications, language, and contact with the attending researchers (see Supplementary materials). Condition assignment was randomized in Qualtrics and configured to maintain balanced allocation across the available testing time slots (9 a.m.–5 p.m.) and testing days, reducing the likelihood that any single condition disproportionately occurred at a particular time of day.

### 2.2 Infrasound administration

**Figure 1:**
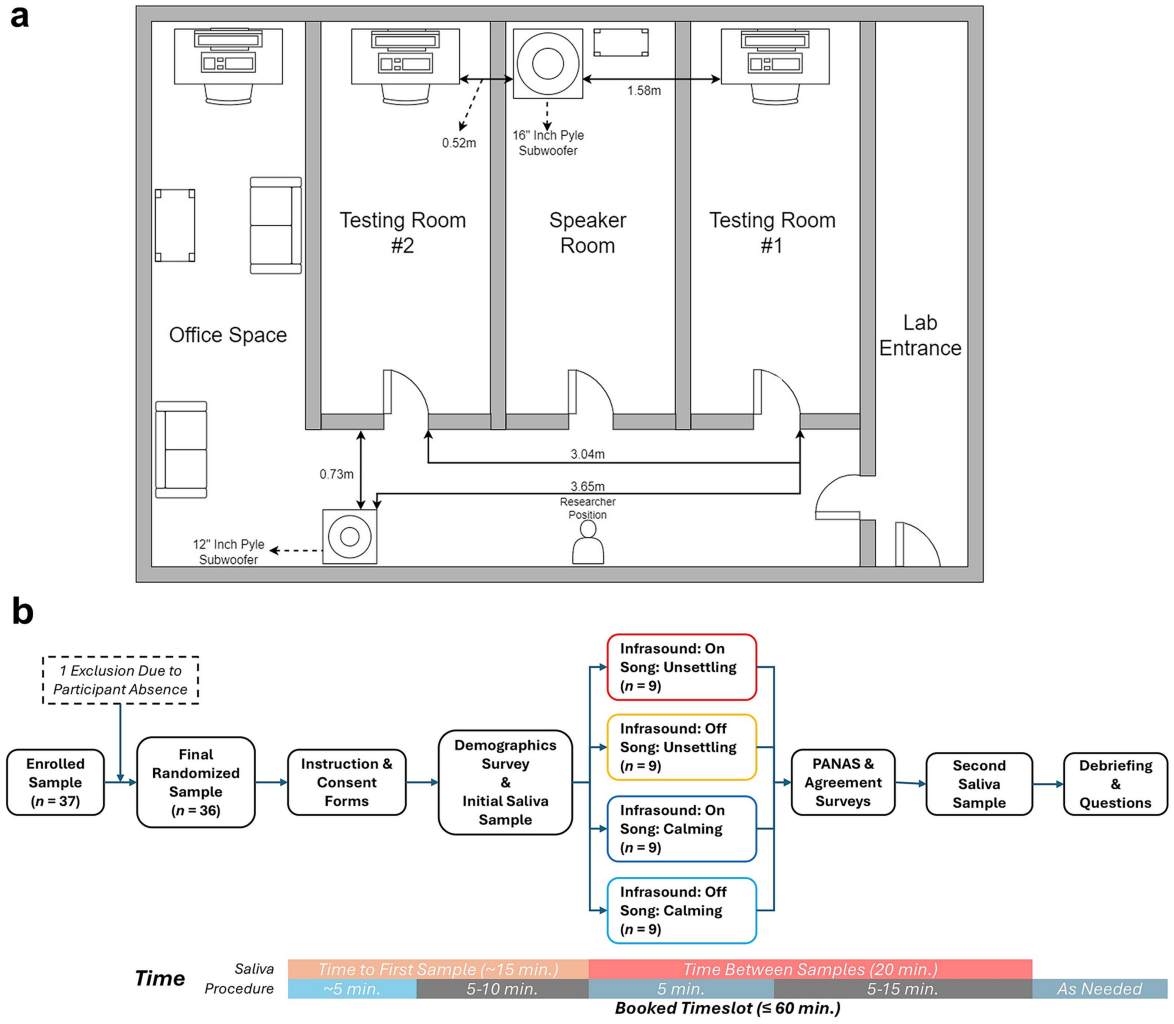
Visual layout and methodology of participant trials. **(a)** Layout of the testing area and equipment used in producing infrasound. Solid arrowed lines represent distances between points. **(b)** Procedural flowchart and timeline of participant testing, including group allocation (2 × 2: infrasound on/off × music type calming/unsettling) and saliva collection timing relative to audio exposure (baseline immediately pre-exposure; second sample 20 min after audio onset).

Infrasound was generated via two out-of-sight subwoofer speakers: A 12″ Pyle subwoofer oriented toward the participants from the hallway outside of the testing rooms, and a 16″ Pyle subwoofer oriented toward the ceiling located in a room between the two testing rooms (Figure 1a). This two-speaker configuration was identified during initial setup and in-room validation after testing multiple configurations, as it provided stable frequency and amplitude measurements at the participant location in both testing rooms while minimizing potential non-acoustic cues (e.g., audible room resonance, mechanical artifacts) that could unblind the condition.

The testing rooms were 3.04 m apart with the 12″ speaker positioned adjacent to the left testing room, and the 16″ speaker positioned 0.61 m from the left testing room and 1.52 m from the right testing room. Calming and unsettling music was played to participants via separate consumer-grade computer speakers connected to the survey computers. The infrasound speakers were each connected to PI-9598 signal generators (Pasco Scientific, Roseville, CA, United States) and participants were exposed to infrasound frequencies centered ~18 Hz collectively between the two sources. 18 Hz was selected as the infrasound stimulus based on prior animal findings showing aversive responses ~15 Hz with similar trends toward 20 Hz (Scatterty et al., 2023) in combination with testing room optimization to achieve stable, validated stimulus delivery at the participant location.

The infrasound generator was optimized so that the sine wave amplitudes and frequencies used were stable with little fluctuation and did not cause readily observable mechanical disturbances due to room resonance. Infrasound frequencies were validated in each testing room using a Spider −20 microphone with Spider EDM 6.0 software (Crystal Instruments, Santa Clara, CA, United States) and were consistently present at an amplitude between 75–78 dB in both rooms. This amplitude range was considered safe for human exposure for the duration of the testing period^1^. The achieved amplitude range was also within the range of those commonly generated by mechanical energy, ventilation, and heating technologies that often lie between 70–80 dB within a distance of 100 m from the source (Salt and Hullar, 2010; Persinger, 2013; Chaban et al., 2021; Butkus and Vasiliauskas, 2013). With the infrasound generator off, a stable baseline of existing background low-frequency noise was established across both testing rooms. Turning the generator on consistently produced a distinct peak around 18 Hz that was ~35–40 dB above baseline. Corresponding FFT/spectral power visualizations comparing OFF versus ON conditions are provided in the Supplementary materials (see “Infrasound Testing Room Validation Notes”).

Ambient light and temperature conditions in each testing room were consistently maintained across days, and participants, as well as their assigned groups, were randomly distributed between rooms to control for potential undetected confounding differences.

### 2.3 Music stimuli

Participants were exposed to one of two audio clips, designed to be either *calming* or *unsettling*. These types were selected by the researchers to represent contrasting affective valence; *calming* expected to elicit typically positive responses, *unsettling* expected to elicit negative. The calming clip was instrumental and intended for meditation, whereas the unsettling clip consisted of horror-themed ambient audio intended to elicit discomfort. Clips were presented *via* consumer-grade computer speakers connected to the survey computers and were approximately 5 min in duration. Participants were randomly assigned to each music condition (*calm* vs. *unsettling*) and to concurrent infrasound exposure (*on vs. off*), resulting in a 2 × 2 design. The audio clips used in this study are provided in the Supplementary materials.

Music was selected as an audible stimulus known to evoke affective responses in humans, providing a sensitive context for testing whether infrasound alters affective experience and evaluation of auditory stimuli, while explicitly modeling music valence in the factorial design. Choosing music types that contrasted in positive and negative valence also allowed for evaluation of whether the cortisol measure was sensitive to affective responding, should each music type elicit the expected response (increase for *unsettling*, decrease for *calming*). Music files were high-pass filtered to remove unintended low-frequency content in the infrasonic range. Similarly, the computer speakers used for music playback were not expected to produce sub-20 Hz output.

### 2.4 Self-report measures

A survey was designed and administered using the online survey platform Qualtrics (Qualtrics, 2023, Provo, Utah, United States, https://www.qualtrics.com/) including timed questions to ensure participants completed the testing period within the same time frame. In addition to written consent forms, participant consent was also collected within the survey. The survey included demographic and medication status items, followed by affect and stimulus-evaluation measures described below.

#### 2.4.1 Positive and negative affect schedule (PANAS)

Participants completed a 20-item Positive and Negative Affect Schedule (PANAS) (Watson et al., 1988) immediately following the music clip, using the standard 5-point intensity scale (“*very slightly*” to “*extremely*”) to rate how they felt at that moment. The PANAS was selected for its strong psychometric properties, including extensive validation as a measure of both baseline and experimentally altered positive and negative affect (Watson et al., 1988; de Carvalho et al., 2013; Crawford and Henry, 2004; Díaz-García et al., 2020). In addition to the standard PANAS, participants completed two brief sets of custom items designed to capture (i) affective evaluation of the music clip (14 descriptors) and (ii) self-reported description of affect during the clip (12 descriptors), each rated on a 9-point agreement scale (“*strongly disagree*” to “*strongly agree*”). The full item wording and response formats are provided in the Supplementary materials.

### 2.5 ELISA cortisol assay

Cortisol is an established hormonal biomarker for stress (James et al., 2023) and is suggested to also play a role in negative affective states (Qin et al., 2016). Four Invitrogen Cortisol Competitive Human ELISA Kits were purchased from Fisher Scientific (Fisher Scientific Company, 112 Colonnade Road, Ottawa, ON) and stored in a—20 °C freezer prior to use, as per supplier recommendation. ELISA assays are commonly used to detect cortisol levels in urine, blood, feces, and saliva and have been well validated both internally by the supplier and externally by third-party studies (Invitrogen, 2018; Gholib et al., 2019; Thomsson et al., 2014; Gatti et al., 2009). All cortisol testing took place in a biological sciences laboratory at MacEwan University and analyses of samples were conducted using a Beckman Coulter DTX880 Multimode Detector (Beckman Coulter Inc., Indianapolis, IN, United States) and Multimode System Software (Version 3.3; Beckman Coulter Inc., Indianapolis, IN, USA).

All ELISA testing was carried out as outlined by the manufacturer protocol. All samples were run in duplicate; thus, two ELISA plates were required to determine the optical density of each sample. Plate 1 consisted of the “before” and “after” samples of participants 1 to 19 and Plate 2 consisted of the “before” and “after” samples of participants 20 to 36. Plates were run at an optimized dilution ratio of 1:2 and only one sample was deemed to be unfit and excluded from statistical testing due to an anomalously low cortisol concentration and markedly low viscosity consistent with water dilution (suspected to be rinse water rather than saliva). All duplicate values were checked for anomalous discrepancies and averaged when none were found.

### 2.6 Saliva collection

Participants were welcomed individually into the lab and assigned to one of two isolated testing rooms (Figure 1a). Following the consent procedure and an overview of the study, participants followed computerized instructions as the researcher relocated to an adjacent room containing the infrasound generator. At this point, the researcher learned of the infrasound condition *via* a face-down, randomized notepad and either activated the infrasound at an amplitude between 75–78 dB in both rooms or left it inactive. No further researcher-participant interaction occurred until data collection was complete. The researcher was blind to both song and infrasound condition in all interactions with the participants prior to data collection.

Each participant was exposed to a single musical stimulus. For participants in the infrasound condition, infrasound exposure lasted for a duration of 4 min and 40 s, giving the researcher time to subtly turn the infrasound on and off at the beginning and end of each ~5-min music clip. Immediately following, participants completed the modified PANAS. This assessed their current affective state, evaluation of the music, and affective experience during the music. Following the completion of the PANAS, participants were asked to indicate whether they thought the infrasound was on during the music clip. All post-exposure surveys were completed immediately after the audio clip, and participants were instructed to provide their second saliva sample 20 min after audio onset, regardless of whether the survey had been completed yet. Participants who gave their second sample mid-survey were then instructed to continue the survey until completion. A visual summary of the protocol sequence and key timing anchors, including saliva collection relative to audio onset, can be found in Figure 1b.

#### 2.6.1 Saliva collection

On each desk in the testing rooms, two 5 mL Eppendorf tubes were placed open and with a marked line at the 1 mL point within fields on a sheet of paper labelled “before” and “after.” Eppendorf tubes have been used in previous studies collecting saliva samples and were considered appropriate for this study (Mohanan et al., 2019; Bellagambi et al., 2020; Mohamed et al., 2012). Beside the tubes were two ~3-inch lengths of plastic straw, sterilized prior to testing. Small plastic cups were filled half-way with clean drinking water and placed at each desk for mouth rinsing. A point-form instruction sheet, as seen in the Supplementary materials, was provided to remind participants of the collection steps and when to give samples. Prior to the survey and music clips, participants were given a demonstration of each step of the saliva collection. Participants were instructed to rinse their mouths prior to giving their samples by swishing their mouths with water and swallowing. The rinsing procedure was to be done 3 times before collection to standardize sampling and minimize potential contamination from residual food, beverages, or oral hygiene products present earlier in the day. Participants were then to spit through the straw into the Eppendorf tube and aim to fill it approximately to the marked 1 mL line. After sample collection, participants were instructed to close the Eppendorf tube securely and place it back on the desk in the corresponding “after” field. A new length of straw was used for each sample. The researcher present was to ensure that the pre-stimulus samples were given immediately prior to audio exposure and post-stimulus samples were 20 min after audio exposure. 20 min post-stimulus was determined to be an optimal time to detect changes in salivary cortisol levels (Robert-Mercier et al., 2014; Qi et al., 2016; Iqbal et al., 2023).

All collection tubes were pre-labelled with an anonymous participant identification number, with the letter “A” representing their first sample, and “B” representing their second sample (e.g., “12B”). Participants were informed that these numbers were only for data processing and could not be traced back to their personal information. After sample collection, the participant numbers were entered into the end of the survey by the researcher to pair self-report scores to corresponding saliva sample. Following testing and collection, all participants were anonymous from the data.

Upon completion, all sample collection materials (cups, straws, etc.) were discarded and the testing area was sanitized with 70% ethanol. Samples were stored in a tray and transported at the end of each testing day to a −20 °C freezer for storage and later testing. Each sample was only thawed once when used for ELISA. All procedures involving the samples after initial freezing took place in the same laboratory space as the freezer in which they were stored to avoid transport-related thaw events. Researchers wore full-length laboratory coats and nitrile gloves for the duration of each testing period. Gloves were changed between each testing period to ensure sterility and avoid cross-contamination.

### 2.7 Statistical analysis

All data were analyzed using GraphPad Prism (v9.1.2; GraphPad, San Diego, CA, United States), JASP (v0.17.1; JASP Team, 2023), and R Statistical Software (v4.4.1; R Core Team, 2024). Statistical significance was set at *α* = 0.05 (95% confidence). Effect sizes and test values were reported alongside *p*-values to support transparent interpretation. Data are presented as means ± standard error of the mean (SEM). Potential outliers were screened using ROUT (robust regression and outlier removal), which flags observations with unusually large residuals while controlling the false discovery rate. A Q value of 1 (*Q* = 1) was selected, corresponding to a 1% maximum false discovery rate for outlier identification, and no outliers were detected. Normality and variance equality were verified using the D’Agostino–Pearson and Bartlett’s tests, respectively. Given the small group sizes, formal assumption tests were treated as supplementary and interpreted alongside residual diagnostics (e.g., Q–Q and residual-versus-fitted plots). Univariate and small subset analyses of variance (ANOVAs) were conducted across all independent variables. Given that final sample size could not be predetermined, *post hoc* sensitivity analyses were used (G*Power; *α* = 0.05) to estimate the effect sizes detectable with 80% power for the completed design.

Optical density data were modeled in GraphPad as cortisol standard concentration versus optical density for both standards and saliva samples. A non-linear regression model was used to generate the standard curves, and sample cortisol concentrations were interpolated and adjusted by a factor of two to account for 1:2 dilution.

A chi-squared test was used to assess the relationship between self-reported and actual infrasound presence, indicating whether participants could correctly identify and thus consciously detect infrasound in their environment.

PANAS scores were analyzed using 2 × 2 two-way ANOVAs testing effects of song condition (*unsettling* vs. *calming*) and infrasound exposure (*on* vs. *off*). Model assumptions were evaluated using residual diagnostics, including assessment of variance homogeneity. Main and interaction effects were investigated with Tukey’s post-hoc comparisons when appropriate. Where mean differences are reported, they reflect the first-listed level minus the second-listed level (for example, Off—On), so positive values indicate higher scores for the first-listed level. Because this item-level testing increased false-positive risk, and applying a single global multiplicity correction across many different tests could conversely inflate false-negative risk in small samples, item-level results were interpreted as descriptive signals rather than confirmatory findings and were emphasized only when supported by effect sizes and convergent patterns across related measures.

Cortisol data were analyzed using a 2 × 2 × 2 three-way ANOVA with song condition, infrasound exposure, and time (*pre-* vs. *post-stimulus*) as factors. Each PANAS score was coded numerically and entered as a scale (quasi-continuous) covariate as an exploratory adjustment to evaluate whether infrasound effects on cortisol change remained after accounting for post-exposure self-reported affect. Significant effects of infrasound on cortisol change were interpreted as physiological evidence of infrasound-elicited aversion when the estimated effect size magnitude warranted interpretive weight. Any such effects were then cross-referenced with variable importance scores (discussed ahead) to help contextualize the ANOVA results.

To check for participant-expectancy effects, a repeated-measures 2 × 2 ANOVA compared participants’ post-exposure self-reported belief that the infrasound was on/off against its actual presence. Significant effects of participants’ reported detection of infrasound were to be interpreted as a potential participant-expectancy effect.

Finally, a conditional inference forest (CIF) was fitted via R to investigate variable importance. The CIF is a useful tool that accounts for bias toward predictors with many split points and high potential for interactions and is ideal for highly complex data with many variables and more predictors than cases (e.g., in datasets with self-report data from lower sample sizes, relative to the number of predictors) (Levshina, 2020). The results from the CIF provide unbiased importance scores of each predictor which are used to investigate the relationships between the considered variables and the presence of infrasound and cortisol change. R code for this model can be found in the Supplementary materials.

Analyses were structured around two primary outcomes with different measurement structures. The post-exposure self-report outcomes (PANAS and related items) were analyzed as between-subject outcomes in the 2 × 2 design. In contrast, pre and post-exposure salivary cortisol levels were analyzed with time as a within-participant factor alongside the between-subject factors (music condition and infrasound exposure). Exploratory follow-up analyses (e.g., item-level patterns and descriptive variable-importance summaries) were interpreted cautiously and were not treated as confirmatory evidence. This approach was used alongside effect size reporting to provide additional context when interpreting results across multiple, non-matched comparisons. Findings of interest therefore centered on results that reached statistical significance, showed stronger effect sizes, and were consistent across related analyses. Variable-importance scores were also considered descriptively to help contextualize patterns in the data and explore whether variables of interest also ranked highly in predictive importance. These scores were not treated as a substitute for inferential testing or as any form of multiple-comparisons correction; rather, they were considered as one complementary signal alongside *p*-values, effect sizes, and consistency across related measures when prioritizing which patterns warranted discussion. Effect sizes were interpreted as small (*f* ≥ 0.10; *η^2^* ≥ 0.01), moderate (*f* ≥ 0.25; *η^2^* ≥ 0.06), or large (*f* ≥ 0.40; *η^2^* ≥ 0.14).

## 3 Results

We found that participants in the infrasound-on condition reported higher irritation, lower interest, and rated the stimulus as sadder than those in the infrasound-off condition. Infrasound also raised salivary cortisol levels, both independently and in conjunction with irritation and discomfort. These self-report differences and cortisol changes were not influenced by music type nor self-reported perception of the presence of infrasound. Final sample sizes amounted to *n* = 9 participants per group (*n*_total_ = 36; 9 males, 27 females; *μ_Age_* = 23.471 y/o, *SEM* = 1.231, *min.* = 18 y/o, *max* = 36 y/o). One participant was excluded due to poor saliva sample quality, resulting in *n*_total_ = 35. All referenced tables can be found at the end of this article and in the Supplementary materials.

Sensitivity analyses indicated that with *N* = 36, the study had 80% power to detect large between-subject effects (Cohen’s *f* ≈ 0.6; *η^2^* ≈ 0.28) and moderate to large within–between interaction effects in the pre–post model (*f* ≈ 0.23–0.35*; η^2^* ≈ 0.05–0.11), while smaller effects would be expected to have reduced power and should be interpreted with caution.

### 3.1 Participant detection of infrasound

**Figure 2:**
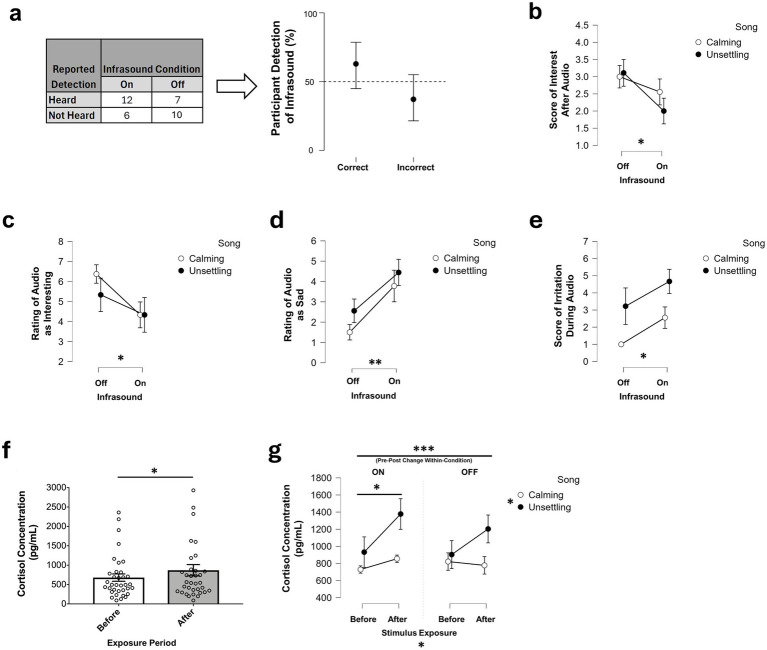
Self-report and hormonal effects of infrasound exposure. **(a)** Accuracy of identifying infrasound during music. **(b–e)** Self-reported interest, music rated as interesting/sad, and irritation by song and infrasound. **(f)** Cortisol change before vs. after exposure. **(g)** Descriptive visualization of cortisol concentration *before* vs. *after* stimulus exposure, by song and infrasound condition; effects shown are derived from the full factorial model and *post hoc* tests (stimulus exposure: *p* = 0.031, *η^2^* = 0.142; song: *p* = 0.019, *η^2^* = 0.084; infrasound on: *p_bonf_* = 0.045; *d* = 0.710; within-condition pre–post: *p_bonf_* < 0.001, *d* = 0.347). Black/white dots show group means ± SEM. Lines connect condition means across infrasound levels within each song condition (not within-participant trajectories); error bars denote SEM and may be absent when SEM = 0. *p < 0.05, ***p < 0.001.

In evaluating the ability of participants to accurately report the detection of infrasound, a Chi-squared independence test between the participant’s reported detection and infrasound returned a *p*-value that was greater than the conventional 5% criterion (Figure 2a; *p* = 0.2406). This suggests that differences in participant responses to infrasound cannot be explained by accurate self-reported perception of the presence of infrasound.

### 3.2 Affective self-report scores

Self-reported scores were evaluated first at the general scale level and then by item. When evaluating general PANAS scores alone, between-condition differences were observed in overall positive and negative affect for post-exposure ratings referring to how participants felt *during* and *after* the audio clip, as well as in ratings of the affective characteristics of the music itself (Supplementary Figure S1; Supplementary Tables S1–S3). Though not significant, these trends in differential affective reporting between infrasound conditions prompted closer investigation for item-level differences in the affective variables themselves.

A moderate-to-large main effect of infrasound was found on self-reported interest after exposure (Figure 2b, Table 1; *F*(1, 31) = 4.410, *p* = 0.044, *η*^2^ = 0.121). Participants in the infrasound-on condition reported lower interest than participants in the infrasound-off condition. No further effects were found on all other variables reporting how participants felt after stimulus exposure (Supplementary Figures 2a–g; *p* > 0.05).

**Table 1:** Summary of effects of infrasound on self-reported affective variables.

Self-report variable	Direction	*F*	df	*p (< 0.05)*	*η^2^*	Importance (*μ* > 0.002)
Interest after exposure	↓	4.410	(1,31)	0.044*	0.121	0.004
Music rated as interesting	↓	4.290	(1,31)	0.047*	0.118	0.006
Music rated as sad	↑	11.078	(1,31)	0.002**	0.253	0.019
Irritability during exposure	↑	4.176	(1,31)	0.049*	0.096	0.008

Directional arrows represent elevation or reduction in reporting. F corresponds to the test statistic yielded by 2 × 2 two-way ANOVA. *η*^2^ (eta-squared) denotes effect size. Importance reflects mean variable-importance scores from the conditional inference forest; values > 0.002 exceeded the minimum importance threshold used to identify informative predictors of infrasound exposure. Full outputs for each effect can be seen in the Supplementary Tables S10–S13. *p < 0.05, **p < 0.01.

For participant description of the music, there was a moderate-to-large main effect of infrasound on whether participants found the music interesting (Figure 2c, Table 1; *F*(1, 31) = 4.290, *p* = 0.047, *η*^2^ = 0.118) in which participants reported finding the music less interesting when the infrasound was on. Participants also showed a large main effect of infrasound on whether they found the music sad (Figure 2d, Table 1; *F*(1, 31) = 11.078, *p* = 0.002, *η*^2^ = 0.253) when the infrasound was on. No further effects were found on all other variables reporting how participants described the music (Supplementary Figures S3a–f; *p* > 0.05).

A main effect of infrasound on self-reported irritability was observed (Figure 2e, Table 1; *F*(1, 31) = 4.176, *p* = 0.049, *η^2^* = 0.096), indicating an increase in irritability levels among participants during infrasound exposure to the music clip. Analysis of other emotional responses during the music exposure revealed no additional effects (Supplementary Figures S4a–f; *p* < 0.05), indicating that the observed infrasound effect may be specific to irritability without a broader impact on participant emotional states.

### 3.3 Cortisol levels

Irrespective of self-reported affective scores, cortisol concentrations were higher post than pre-exposure in the infrasound-on condition (Figure 2f; *W*(35) = 192, *p* = 0.022, *r_rb_* = 0.390) demonstrating an increase in cortisol after stimulus exposure. However, exploratory covariate-adjusted analyses indicated that infrasound-related cortisol change effects persisted after accounting for post-exposure self-reported affect. These covariate models were included as a robustness check, not as confirmatory tests of higher-order interactions.

Assumptions of normality and homoscedasticity were met for group self-report scores and cortisol levels, and a Three-Way ANOVA model (*calm* vs. *unsettling,* infrasound *on* vs. *off, before* vs. *after* cortisol level) with the PANAS variables as covariates was considered suitable for the data. After the music clip, feeling upset had a large main effect on cortisol change (Supplementary Table S4; *F*(1, 30) = 10.413, *p* = 0.003, *η*^2^ = 0.215) and feeling guilty had a small interaction effect with stimulus exposure on cortisol change (Supplementary Table S5; *F*(1, 31) = 4.772, *p* = 0.037, *η*^2^ = 0.014), though both results were expected. Interestingly, infrasound had a main effect on cortisol change even when accounting for significant feelings of irritability (Table 2; *F*(1, 31) = 4.557, *p* = 0.041, *η*^2^ = 0.106), as well as a moderate main effect on cortisol change when accounting for feelings of fear (Table 2; *F*(1, 31) = 4.229, *p* = 0.049, *η*^2^ = 0.093). All other effects of infrasound on cortisol change accounting for self-report scores after the music clip were not significant (Supplementary Table S6).

**Table 2:** Summary of infrasound-related effects on salivary cortisol across covariate-adjusted and unadjusted models.

Self-report covariate	Effect on cortisol	*F*	df	*p (< 0.05)*	*η^2^*	Importance (*μ* > 0.002)
Irritability after exposure	↑	4.557	(1,31)	0.041*	0.106	< 0.002
Afraid after exposure	↑	4.229	(1,31)	0.049*	0.093	< 0.002
Bad during exposure	↑	6.612	(1,31)	0.015*	0.125	< 0.002
Irritated during exposure	↑	5.609	(1,31)	0.025*	0.125	0.008

Unadjusted effects	Effect level	Contrast	Effect on cortisol	Inference	*p* (< 0.05)	*η*²
Stimulus exposure	Main	Before *vs.* After	↑	Increase over time	0.031*	0.142
Song	Main	Calming *vs.* unsettling	↑ (calming) ↓ (unsettling)	Parallels song affective valence	0.019*	0.084

Post-hoc comparison	Corrected	Contrast	Effect on cortisol	Inference	*P_bonf_* (< 0.05)	*d*
Infrasound by calming *vs.* unsettling	Bonferroni	Infrasound/calming *vs.* infrasound/unsettling	↑(calming)↓ (unsettling)	Higher cortisol across both song types post-exposure	0.045*	0.710
Infrasound by before *vs.* after exposure	Bonferroni	Infrasound/before *vs.* infrasound/after	↑	Increased cortisol from pre-post within each infrasound condition	< 0.001***	0.347

Directional arrows represent elevation or reduction in cortisol levels associated with the listed factor or contrast. For covariate rows, F, *df*, *p*, and *η*^2^ summarize the infrasound effect on cortisol after adjusting for the listed self-report covariate. Importance reflects mean variable-importance scores from the conditional inference forest; values > 0.002 exceeded the minimum importance threshold used to identify informative predictors of infrasound exposure. Post-hoc *p* values are Bonferroni-corrected. Full outputs for covariate-adjusted effects can be seen in the Supplementary Tables S14–S17. *p < 0.05, **p < 0.01, ***p < 0.001.

During the music clip, infrasound had a large main effect on cortisol change when accounting for reported scores of feeling bad (Table 2; *F*(1, 31) = 6.612, *p* = 0.015, *η*^2^ = 0.125) as well as another large effect on cortisol change when accounting for reported scores of feeling irritated (Table 2; *F*(1, 31) = 5.609, *p* = 0.025, *η*^2^ = 0.125). All other effects on cortisol change accounting for self-report scores during the music clip were not considerable (Supplementary Table S7).

No significant effects were found on cortisol change when accounting for self-reported scores on emotive description of the music clip (Supplementary Table S8).

Within the full unadjusted factorial model (i.e., without self-report covariates), cortisol levels demonstrated a main effect of stimulus exposure (*before vs. after*; *p* = 0.031, *η^2^* = 0.142) and a main effect of song (*calming* vs. *unsettling*; *p* = 0.019, *η^2^* = 0.084), and Bonferroni-adjusted *post hoc* tests indicated significant between-group cortisol changes in the infrasound condition (*p_bonf_* = 0.045; *d* = 0.710) and within-condition pre–post cortisol changes in both conditions (*p_bonf_* < 0.001; *d* = 0.347). A visualization of these interactions between music and infrasound conditions can be seen in Figure 2g; inferential conclusions are based on the full factorial model and are interpreted in conjunction with both statistical significance and effect sizes for magnitude.

### 3.4 Participant-expectancy effects

When evaluating for potential expectancy effects on cortisol change brought about by participant expectations that infrasound was present, it was found that there was no main effect of participants’ self-reports (Supplementary Figure S5; Supplementary Table S9; *F*(1, 31) = 0.019, *p* = 0.891, *η*^2^ < 0.001) nor an interaction effect with the actual presence of infrasound (*F*(1, 31) = 0.166, *p* = 0.687, *η*^2^ = 0.005) on cortisol change. This suggests that participants did not show elevations in cortisol levels due to preconceptions that there was infrasound present.

### 3.5 Investigating variable importance *via* conditional inference forests

**Figure 3:**
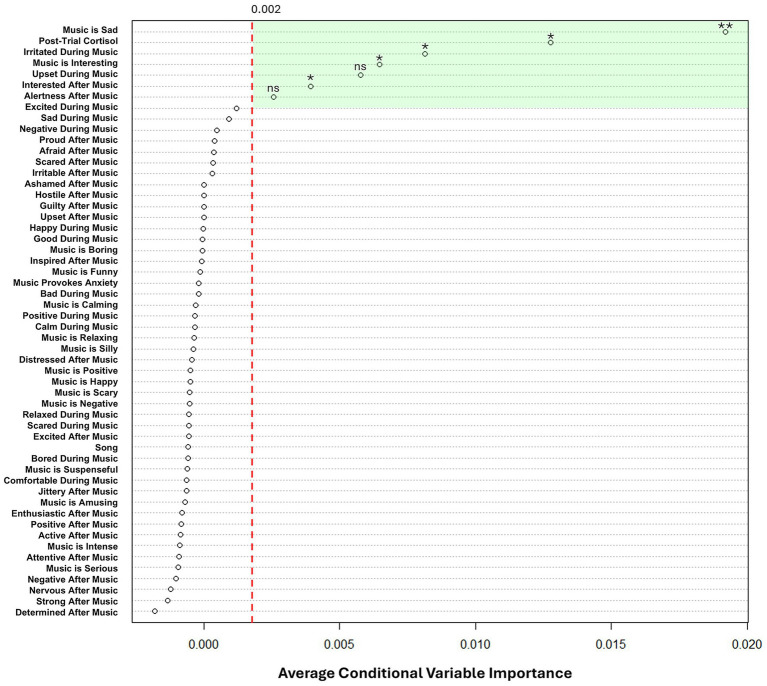
Exploratory importance rankings of variables as predictors of the presence of infrasound *via* a conditional inference forest (CIF). Conditional variable importance scores from a conditional inference forest (25 trials). *X* axis: Study variables; *Y* axis: Conditional importance. Red dashed line = minimum importance threshold (>0.002). Variables both significant and important were considered for interpretation; variables above threshold but not significant indicate likely interaction. Significance values are from univariate tests, not the CIF. ns, Not significant, *p < 0.05, **p < 0.01.

A conditional inference forest (CIF) was implemented to investigate variable importance with respect to the presence of infrasound. In this analysis, a CIF containing 500 trees was fitted to 25 random permutations of the variables of interest. After averaging importance scores, an absolute minimum of the mean importance scores was calculated at *μ* = 0.002. Notably, participant descriptions of the music as sad (*μ* = 0.019, *SD* = 0.002), cortisol levels after the audio exposure (*μ* = 0.012, *SD* = 0.003), irritation during the music (*μ* = 0.008, *SD* = 0.002), description of the music as interesting (*μ* = 0.006, *SD* = 0.002), feeling upset during the music (*μ* = 0.006, *SD* = 0.002), feelings of interest after exposure (*μ* = 0.004, *SD* = 0.002), and feelings of alertness (*μ* = 0.003, *SD* = 0.002) were the only variables with average importance score above μ = 0.002. These results can be seen in Figure 3.

A second CIF was fitted to assess variable importance with respect to cortisol change. Following the procedure outlined for infrasound, we calculated the absolute minimum of the mean importance score to be *μ* = 0.002. Notably, the presence of infrasound returned an average importance score (*μ* = 0.06, *SD* = 0.01) above *μ* = 0.002. Supplementary Figure S6 of the supplemental materials displays the results from this CIF which shows that the distributions of variable importance score for both infrasound and cortisol change are similar.

## 4 Discussion

### 4.1 Infrasound elicits negative affective and physiological responses

In line with our primary aims, infrasound exposure was associated with more negative self-reported responses and increased salivary cortisol. These findings are interpreted in context of the post-exposure self-report and pre–post cortisol designs. Participants in the *infrasound-on* condition reported more negative post-exposure affect than those in the *infrasound-off* condition, across music conditions. Self-report measures showed that infrasound decreased interest in the music, increased irritation during listening, and increased descriptions of the music as sad across both conditions. Self-report scores also validated that calming music increased positive affect and unsettling music increased negative affect, irrespective of infrasound. Infrasound elevated cortisol levels in participants who reported greater feelings of guilt and upset while showing similar effects independent of feeling bad and irritated. This suggests that although the *infrasound-on* condition was associated with more negative post-exposure affect (particularly higher disinterest and irritation), cortisol levels increased even when accounting for heightened negative mood states. This highlights both subjective and physiological evidence of infrasound-elicited negative affect and aversion. Importantly, no noteworthy effects of infrasound were found on measures of positive affect and CIF results were broadly consistent with the significance findings, where levels of cortisol, sadness, irritation, and interest were important predictors. These findings appear to support previous studies suggesting that infrasound may increase negative affect and evoke aversive responses in humans.

### 4.2 Absence of conscious detection and expectancy effects

Participants appeared to be no better than chance at identifying the presence of infrasound, and whether the participants’ thought infrasound was present or not was found to score low as a predictor of the presence of infrasound. In addition to this, participant expectations of the presence of infrasound did not show a statistically detectable association with cortisol level change. This indicates that emotional and physiological changes were unlikely to be explained by conscious audition or participant-expectancy effects. Such findings support prior research indicating that although humans may not typically audibly detect infrasonic frequencies (i.e., below ~20 Hz) (Bedard and Georges, 2000; Møller and Pedersen, 2004), the presence of infrasound may still impact emotional states or behavioral responses (Persinger, 2013; Leventhall, 2007; Møller and Pedersen, 2004). Participants who were exposed to infrasound reported feeling less interest and described the music clip itself as less interesting and more sad than those who were not. Coupled with increased irritability during the music clip with infrasound on, these findings suggest that infrasound may be aversive to humans and may negatively impact mood.

### 4.3 Infrasound evokes irritability and annoyance rather than anxiety

Interestingly, increases in self-reported anxiety were not detected, despite common suggestions that infrasound may be anxiogenic. Heightened irritability, however, without an increase in anxiety-related variables parallels previous research suggesting that infrasound intensifies annoyance (Persinger, 2013; Møller, 1984) rather than anxiety (Scatterty et al., 2023). Animal models have been used to study the adverse affective impact of infrasound on behaviour, with some fish species exhibiting aversive responses to infrasound (Scatterty et al., 2023; Karlsen, 1992a; Karlsen, 1992b; Enger et al., 1993; Sand and Karlsen, 1986; Sand and Karlsen, 2000; Bui et al., 2013; Karlsen et al., 2004; Sand et al., 2001) that can commonly be interpreted as anxiety-like or fear-like behaviors. These aversive effects were found at amplitudes and frequencies similar to those that might be experienced when in the vicinity of human infrastructures (Salt and Hullar, 2010; Persinger, 2013; Butkus and Vasiliauskas, 2013) and those used in this study. For example (Scatterty et al., 2023), found that 15 Hz infrasound triggered aversive behavioral responses in wild-type zebrafish (*Danio rerio*) during a controlled open field test. In these fish studies, animals detected infrasound via the otolithic organs. While humans perceive auditory stimuli instead via cochlear transduction, otoliths are still present and responsible for similar vestibular senses, suggesting that infrasound may be detected through sub-auditory perception (Salt and Hullar, 2010; Karlsen, 1992b; Enger et al., 1993; Sand and Karlsen, 2000). It is possible that terrestrial vertebrates (e.g., humans) retained infrasonic perception through the otoliths as a non-auditory sense, while audition migrated to the cochlea over time. Furthermore, the extensive connectivity between the vestibular system and the limbic system in humans offers potential insights as to how vestibular sensation influences emotional responses (Rajagopalan et al., 2017; Neumann et al., 2023; Uno et al., 2006).

### 4.4 Relationship between irritation, cortisol, and aversion

The alterations in cortisol further strengthen the connection between infrasound exposure and human aversion. Cortisol was interpreted as a valence-nonspecific index of stress-related arousal, while self-report measures were used to provide affective context for interpreting the direction of experience. Infrasound increased cortisol levels even when accounting for the heightened feelings of irritability already found in the self-report measures. Infrasound likewise increased cortisol when accounting for feelings of fear and discomfort (feeling “bad”). Marginal evidence was also noted in that infrasound may have detrimental effects on levels of excitement and sadness as well. These results agree with previous findings that infrasound elicits annoyance both behaviorally and physiologically (Persinger, 2013; Møller, 1984). Interestingly, perception of the music as ‘sad’ was both the most significant and most important predictor of the presence of infrasound and second most important predictor of cortisol change. This could be interpreted as an affective component that complements the effects on irritation and disinterest, expressed as resulting emotional discomfort that negatively impacts mood.

The negatively affective element to the apparent irritant effects of infrasound further raises concerns about what effects more prolonged exposure could have on emotional well-being over time. These findings were independent of any interaction with the passage of time (*before vs. after*), demonstrating that infrasound had such effects regardless of any innate cortisol increase occurring over the trial period (i.e., participants becoming frustrated with the length of the experiment). Importantly, the effects of infrasound condition (*on vs. off*) on cortisol were observed across both music conditions, indicating that this pattern was also not likely driven by or confounded with music type. Prior room validation further suggests it is unlikely that uncontrolled background infrasound differences account for these findings.

### 4.5 Limitations and directions for future research

The patterns of irritation and disinterest seen in this study, interpreted as an involuntary aversive reaction, correspond to the avoidance responses observed in zebrafish by Scatterty et al. (2023), demonstrating infrasound-elicited aversion. To further explore this finding, future studies could investigate the irritant and aversive effects of infrasound using tests specifically designed to detect differences and changes in each, such as the Brief Irritability Test (Holtzman et al., 2015) or the Multiple-Item Annoyance Scale (Schreckenberg et al., 2018). This study also only exposed participants to a condition where infrasound was either present or absent and relied heavily on post-exposure self-report measures to indicate emotional affective responses. To strengthen the evidence of the irritant and aversive properties of infrasound behaviorally, future studies could employ a forced-choice task where participants choose between audio stimuli with or without infrasound. This would assess whether participants prefer to avoid stimuli containing infrasound frequencies. Similarly, PANAS data were only collected after exposure without a baseline for comparison, making within-subjects verification of self-reported affective change difficult to quantify or verify. Accordingly, PANAS findings should be interpreted as post-exposure differences between randomized conditions. A pre-exposure PANAS may also introduce priming or expectancy effects by cueing participants to monitor specific emotions during the subsequent infrasound exposure, and therefore this limitation may simply present as a trade-off in design between experimental control and baseline comparison.

Regarding frequency, only one target was used (~18 Hz) based on previous findings in zebrafish (Scatterty et al., 2023), stimulus validation, and room optimization. While this target was deemed appropriate for this study, future studies should explore whether similar or null effects can be observed in a range of frequency targets above or below ~18 Hz. The modest sample size and use of a convenience sample of young adult undergraduate students limit generalizability to other age groups and populations. While randomization across conditions in our between-subject design helped reduce the likelihood of individual differences in hearing ability and listening behaviour, future studies should replicate these findings in larger and more diverse samples. Given that the sample was predominantly female, menstrual cycle phase and hormonal contraceptive use represent unmeasured sources of variability that may influence cortisol concentrations and physiological or emotional responsiveness. These variables were not recorded in the present study and should be assessed or considered in future work where feasible.

Importantly, Figure 2f illustrates that music clips without infrasound present yielded the expected effects of calming music, reducing cortisol levels and unsettling music raising cortisol levels. Equally notable was the reversal of the calming music effect and the intensification of response to the unsettling music when infrasound was on. These findings also illustrated that the timing between initial and post-stimulus samples was sensitive enough to detect changes in cortisol levels (Figure 2f). Future studies will validate these findings by analyzing blood or urine samples to confirm salivary measures of cortisol change due to infrasound exposure with stimulus duration and sample collection times adjusted according to the physiology of each method. Although condition assignment was balanced across the testing window and cortisol was assessed using within-participant pre–post change, salivary cortisol reflects a latent physiological response and therefore the pre-exposure sample should not be interpreted as a purely instantaneous baseline. Future studies may further reduce potential for time-of-day effects by restricting sessions to narrower time windows or explicitly modeling specific diurnal time points. Future studies will also revisit animal models, leveraging salivary cortisol as a biomarker for infrasound-elicited irritation, to validate previous findings and strengthen the results of this study. This will include returning to the zebrafish model of infrasound-elicited aversion by Scatterty et al. (2023), assessing replicate animals for cortisol level changes in skin mucus (Carbajal et al., 2019; Kulczykowska, 2019; Franco-Martinez et al., 2022), holding-water concentrations (Félix et al., 2013; Midttun et al., 2022), blood plasma (Sadoul and Geffroy, 2019), or whole-body sampling (Philippe et al., 2023; Ramsay et al., 2009). Replicate experiments using mammalian animal models (e.g., rodents) will also be considered to establish generalizability. Lastly, the evaluation of expectancy effects on cortisol change in this study only assessed participant reports on whether they believed the infrasound was on or off without accounting for how strongly they felt about their answers. A future study may further investigate the effects of participant expectation by informing a participant that the infrasound is on and evaluating their self-report and physiological responses regardless of whether the infrasound is truly on or not.

### 4.6 Conclusion

Overall, this study used a combination of self-report and biological measures to demonstrate that infrasound can have irritant, and aversive properties on humans. Similarly, infrasound appears to influence increases in negative affective evaluation. No evidence was detected in this sample suggesting that infrasound is anxiogenic or elicits positive affect during exposure to positive stimuli, implying that infrasound is associated with negatively affective states and not a general increase in arousal. Considering the prevalence of infrasound in and around human habitats, these findings emphasize the potential value of identifying and mitigating sources of infrasound pollution within our environments.

## Data Availability

The original contributions presented in the study are included in the article/Supplementary material, further inquiries can be directed to the corresponding authors.

## Footnotes

1
www.cdc.gov
