# Supplementary material for: Infrasound exposure is linked to aversive responding, negative appraisal, and elevated salivary cortisol in humans
Source: Front Behav Neurosci. 2026 Apr 27;20:1729876. doi: 10.3389/fnbeh.2026.1729876 (PMC13158192; doi:10.3389/fnbeh.2026.1729876)
Supplement: Supplementary file 2 [file Supplementary_file_1.docx]

**Infrasound Elicits Aversive Responses on Self-Report and Hormonal Markers of Irritation**

**SUPPLEMENTAL MATERIALS**

**Figures**

**
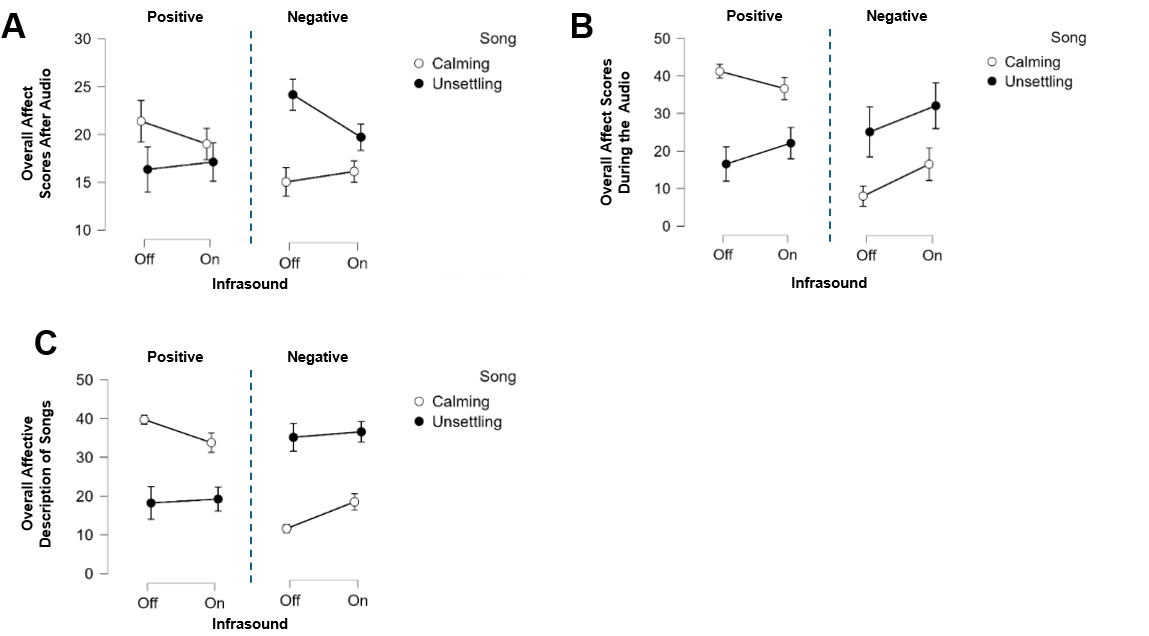
**

**Figure 1:** *Two-Way ANOVA results of overall self-reported PANAS scores.* Interaction plots represent positive and negative affective change (A) after the audio, (B) during the audio, and (C) in participant descriptions of the music. All infrasound results in this figure were non-significant. Error bars represent S.E.M.


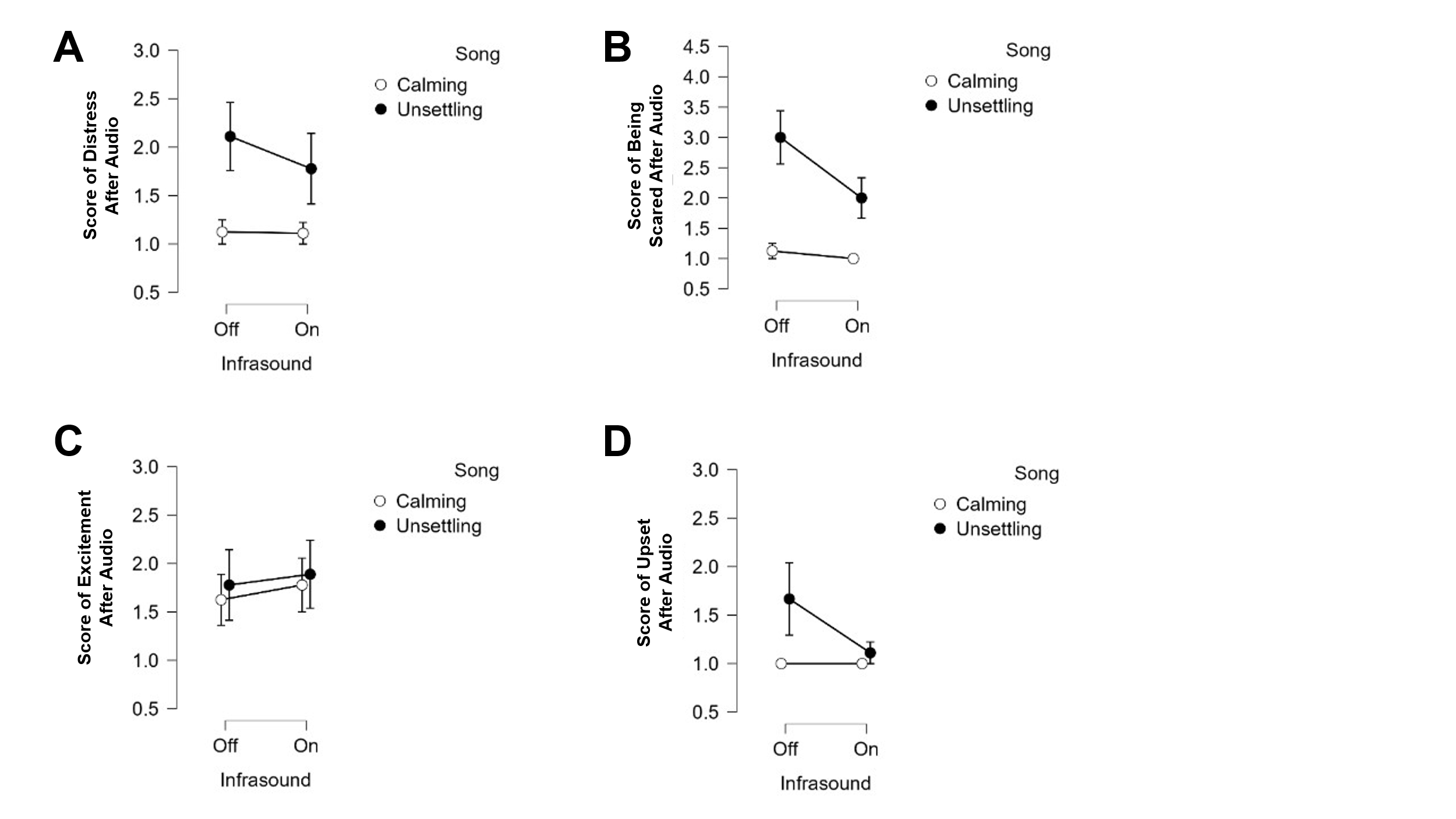


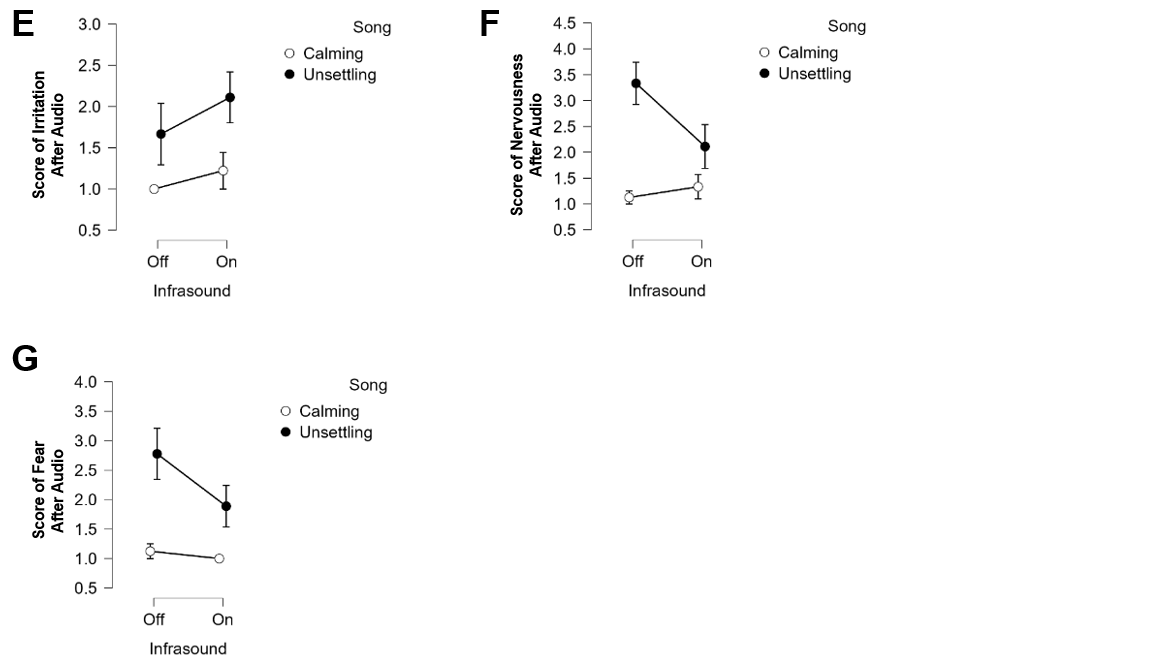


**Figure 2:** *Two-Way ANOVA results of self-reported PANAS scores on how participants felt after the listening period.* Interaction plots represent scores of (A) Distress, (B) Being scared, (C) Excitement, (D) Irritation, (E) Nervousness, and (F) Fear). All infrasound results in this figure were non-significant. Error bars represent S.E.M.


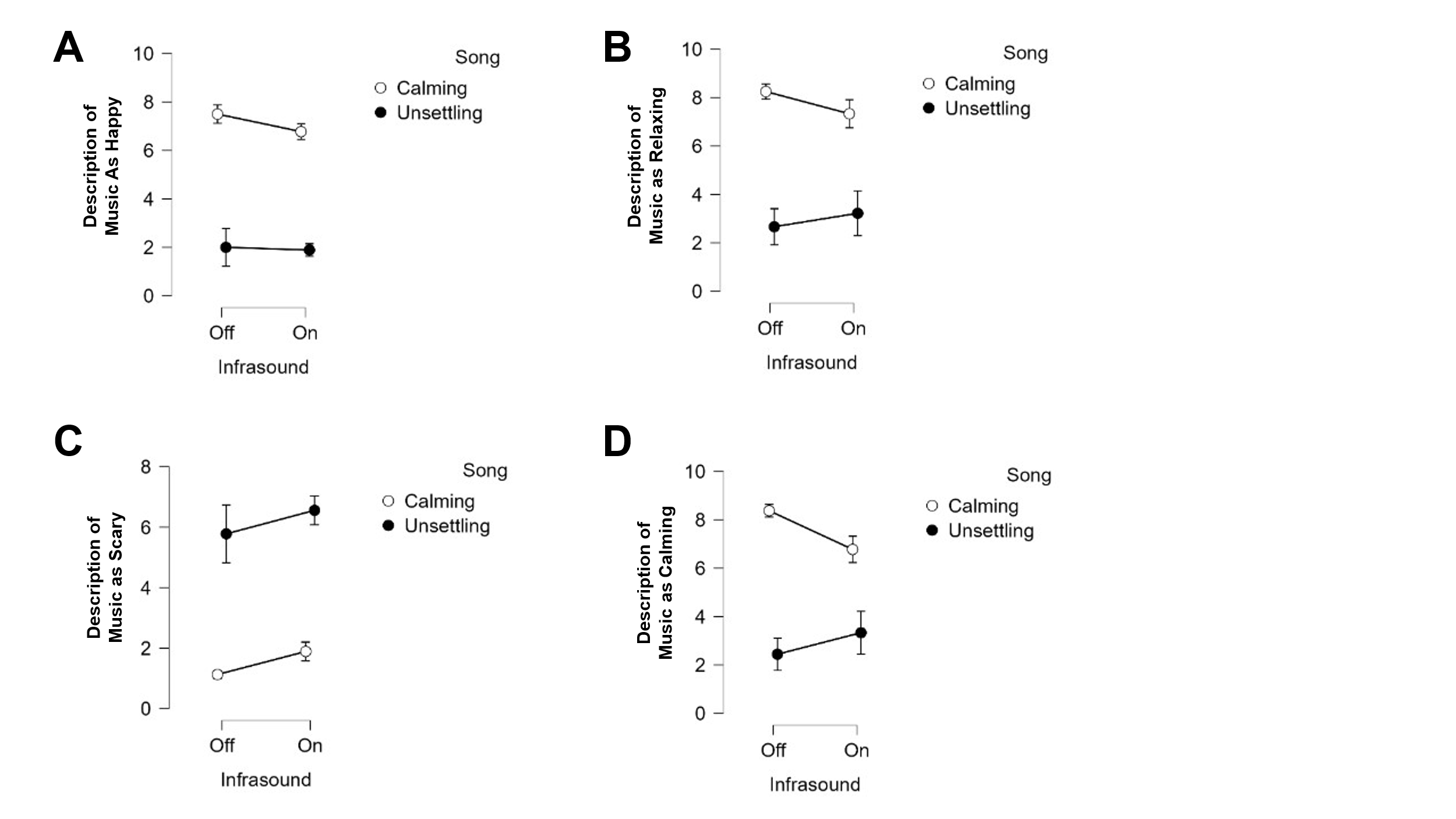


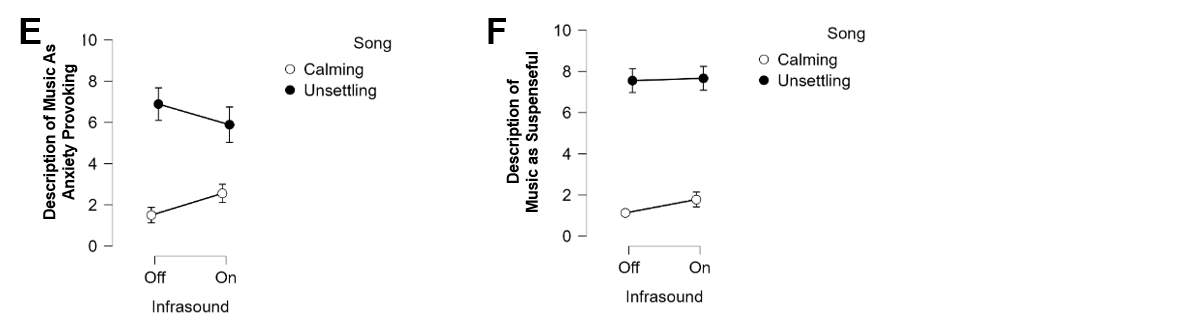


**Figure 3:** *Two-Way ANOVA results of self-reported PANAS scores on how participants described the music clips.* Interaction plots represent participant descriptive scores of the music clip they listened to as (A) Happy, (B) Relaxing, (C) Scary, (D) Calming, (E) Anxiety provoking, and (F) Suspenseful. All infrasound results were non-significant. Error bars represent S.E.M.


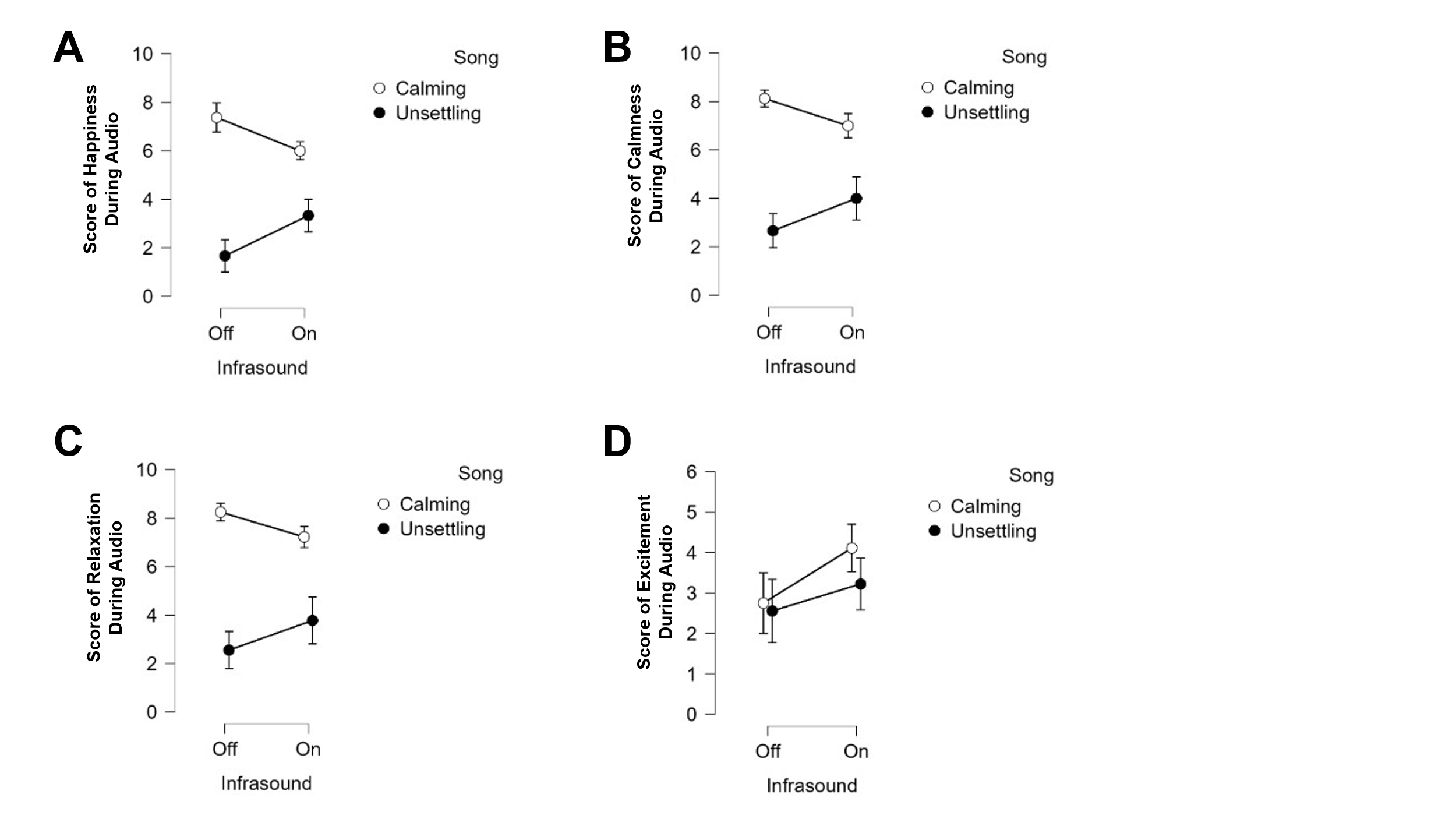


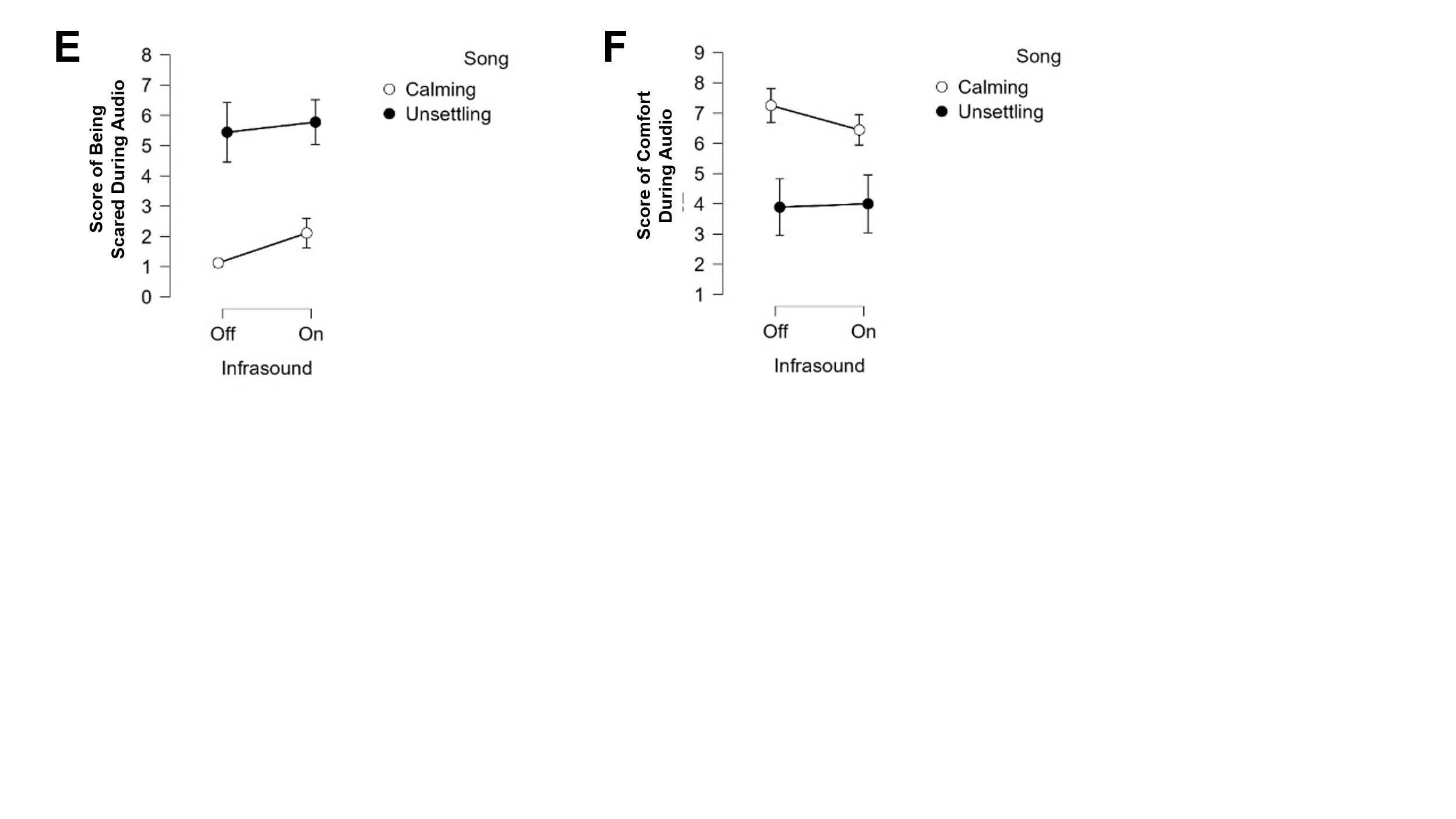


**Figure 4:** *Two-Way ANOVA results of self-reported PANAS scores of how participants felt during the music clips.* Interaction plots represent participant scores of feeling (A) Happy, (B) Calm, (C) Relaxed, (D) Excited, (E) Scared, and (F) Comfortable. All infrasound results were non-significant. Error bars represent S.E.M.


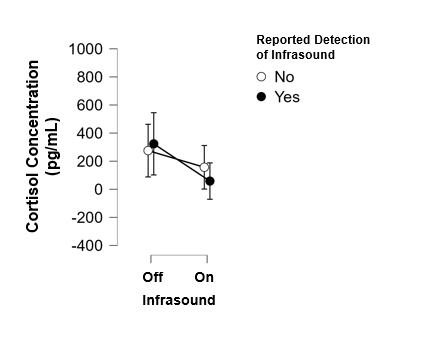


**Figure 5:** *Two-Way ANOVA results of participant expectancy effect on cortisol change evaluation.* The interaction plot represents participants’ cortisol levels based on their reports of the infrasound as being on or off, separated by whether the infrasound was truly on or off. No significant effects were found and an expectancy effect was deemed unlikely. Error bars represent S.E.M.


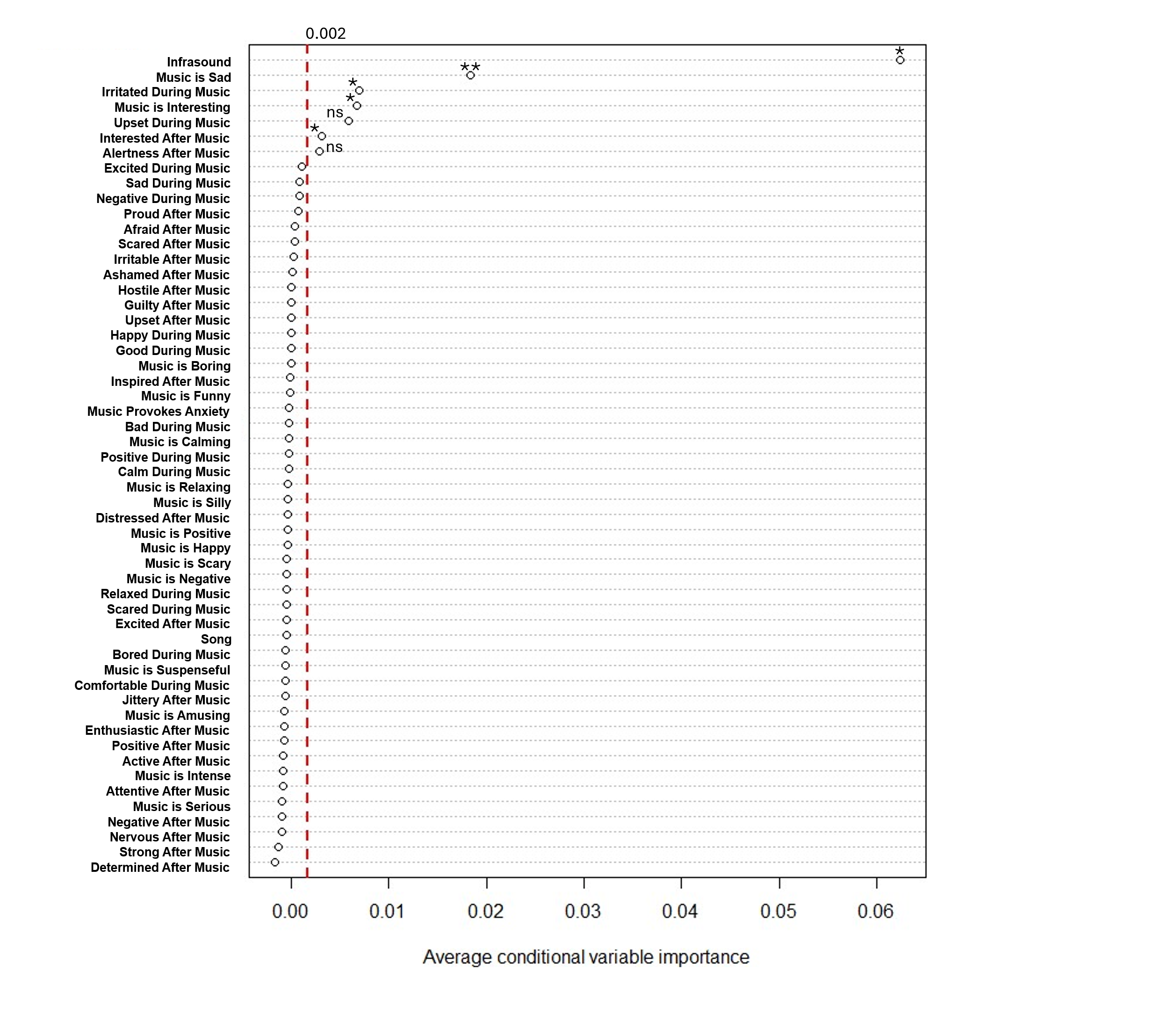


**Figure 6:** *Conditional inference forest results of variable importance in predicting cortisol change.* The x axis represents the independent variables of the study. The y axis represents the conditional importance of the independent variables, averaged across 25 statistical trials. The red dashed line represents the calculated absolute minimum importance value, which acts as a threshold of variable importance to the predictability of how much cortisol levels changed. Only variables that are both significant and important are considered valid predictors of cortisol change. *ns* = Not significant, **p* < 0.05, ***p* < 0.01.


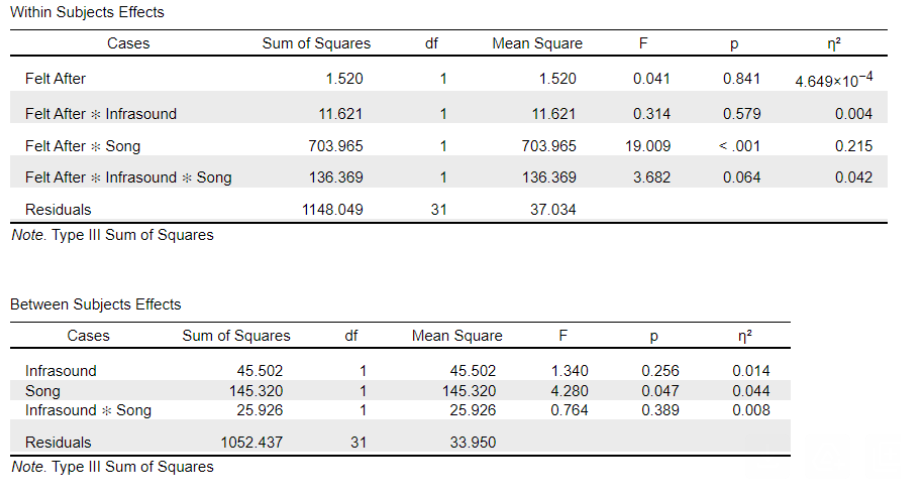


**Table 1:** *Effects of infrasound on overall PANAS affective scores after the listening period.*

*
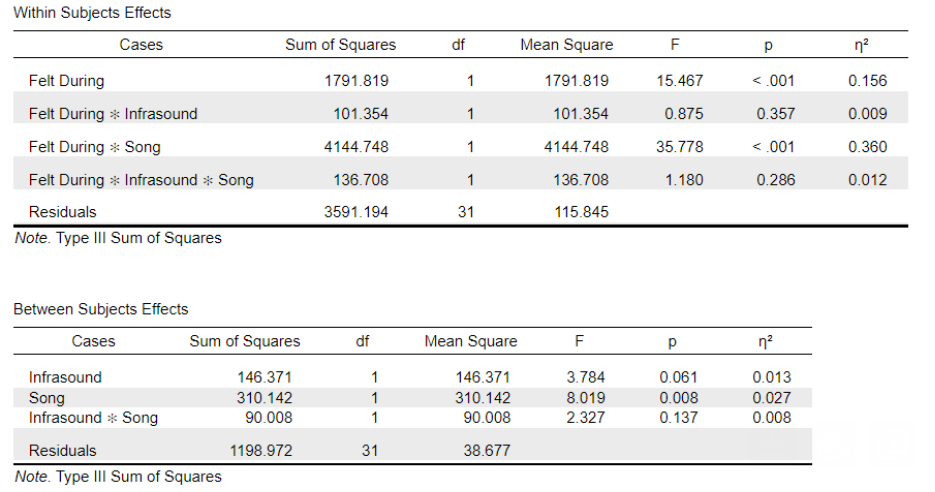
*

**Table 2:** *Effects of infrasound on overall PANAS affective scores during the listening period.*

*
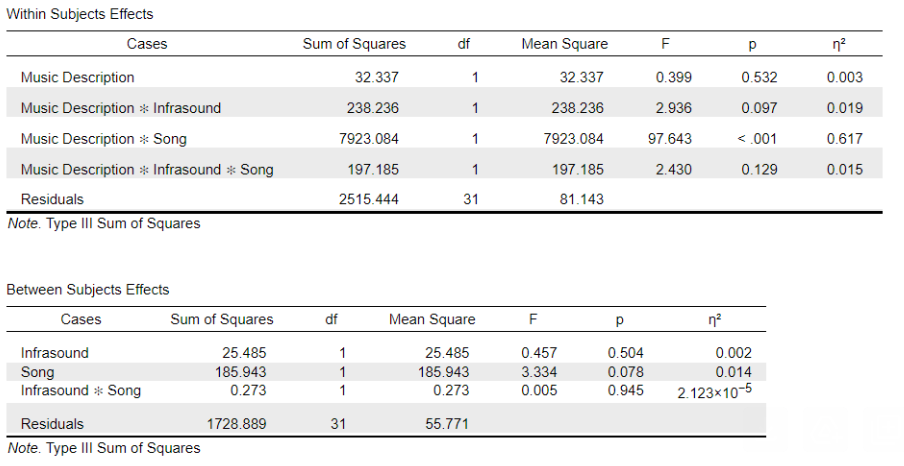
*

**Table 3:** *Effects of infrasound on overall PANAS affective scores of participant descriptions of the music clips.*


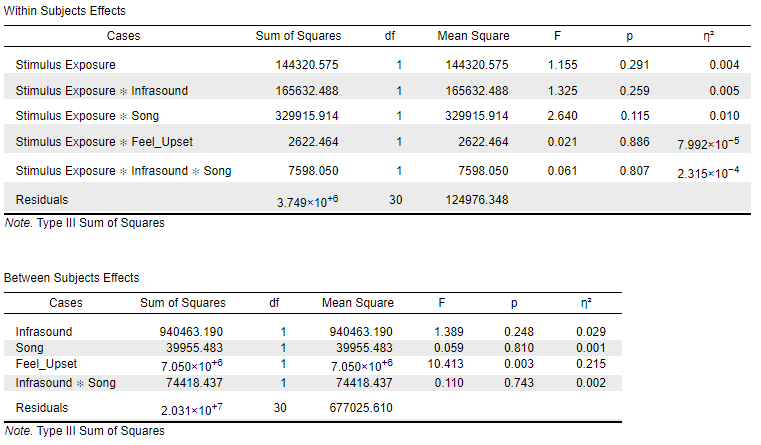


**Table 4:** *Effects of feeling upset on cortisol change by song and infrasound exposure.*


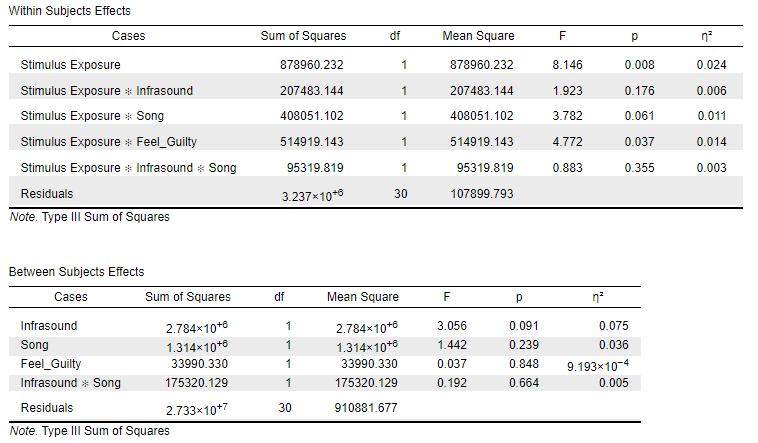


**Table 5:** *Effects of feeling guilty on cortisol change by song and infrasound exposure.*


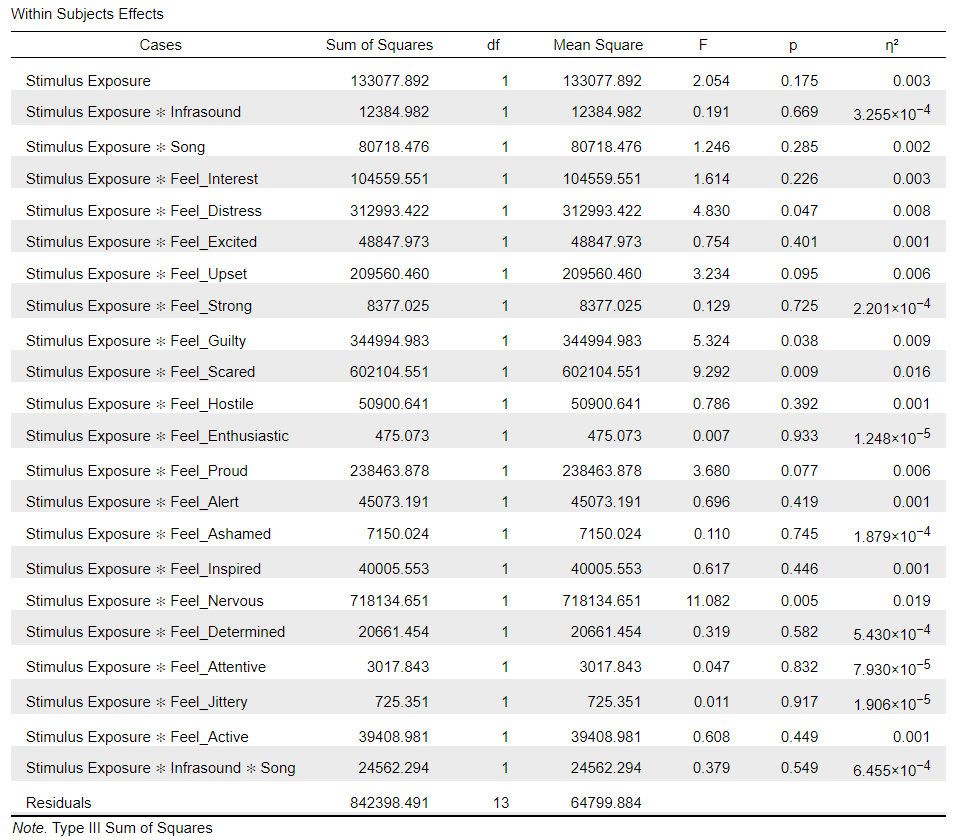


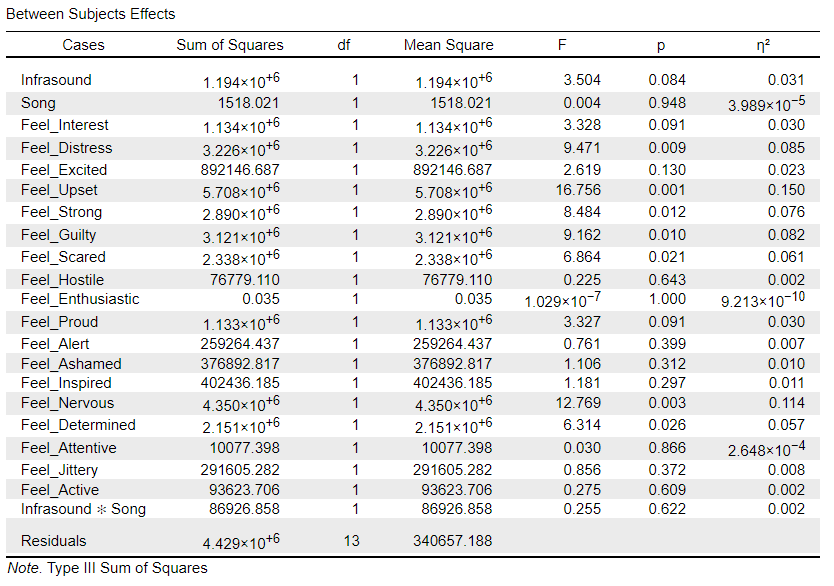


**Table 6:** *Effects of infrasound on cortisol change when accounting for feelings of all non-significant PANAS variables after audio exposure.*


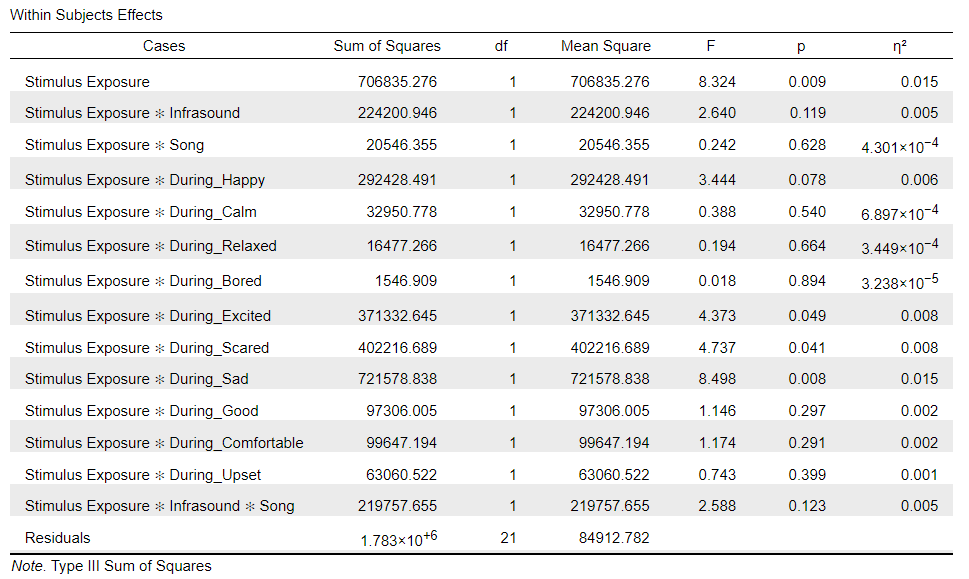


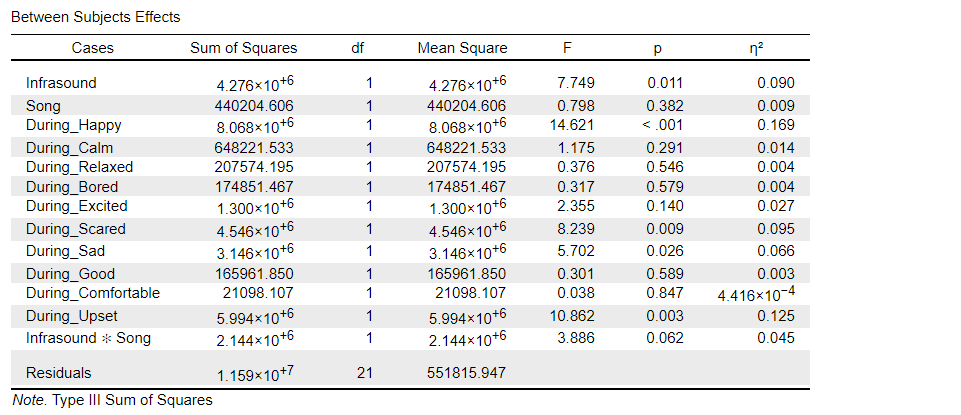


**Table 7:** *Effects of infrasound on cortisol change when accounting for feelings of all non-significant PANAS variables during audio exposure.*


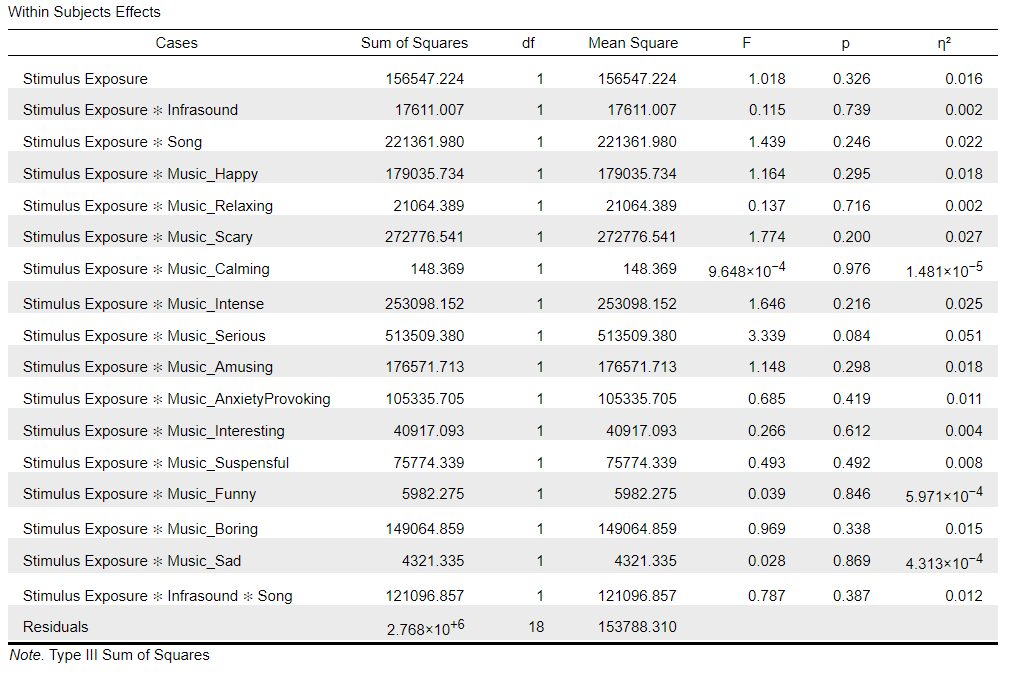


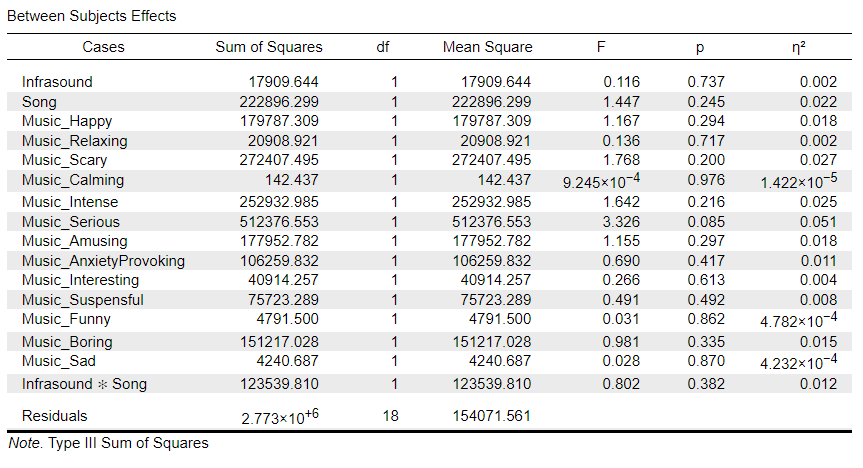


**Table 8:** *Effects of infrasound on cortisol change when accounting for feelings of all non-significant PANAS variables of participant descriptions of the music clips.*


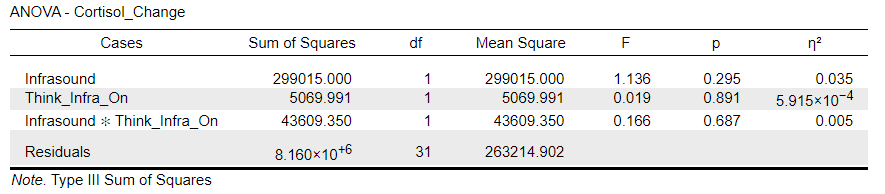


**Table 9:** *Evaluation of potential expectancy effects of participant reporting of presence of infrasound on cortisol levels.*

**Audio Stimuli: Unsettling and Calming Song Clips**

***Unsettling Song***

<https://drive.google.com/file/d/1UxN9EPlBSVUyLgf7vEjjagJLHOHpyMww/view?usp=sharing>

***Calming Song***

<https://drive.google.com/file/d/1KveKTmFwgdLGhzVa8qncZprad2sXr3A4/view?usp=sharing>

**Infrasound Field Recording Notes**

*NOTE: Crystal Instruments service representatives have informed us that this microphone may not be appropriately sensitive to frequencies between 0-5 Hz. All peaks seen in the below figures @ 5 Hz and below should be considered unreliable and can be ignored. These spectra are provided as illustrative examples of the acoustic profiles measured across conditions. They were used qualitatively to verify that infrasonic energy was present at higher amplitude in the infrasound-on condition relative to infrasound-off; no statistical analyses were conducted on these plots. Interpretation focuses on the infrasonic band (<20 Hz) amplitude/peaks.*

**March 30, 2023 – Day 1 – Urban Environment – 11:30 am to 1:15 pm**

Downtown Edmonton, AB (Within and around MacEwan University Main Campus)

*NOTE: The Spider-20 mic accuracy declines below 5 Hz, thus frequencies between 5-19 Hz were the point of focus.

**NOTE: Frequency peaks that were not notably different from frequencies seen above 20 Hz on the spectrum were not considered notable evidence of infrasound. For a sub-20 Hz peak to be considered significant, it would have to be both one of the highest on the spectrum, AND be visibly elevated from the rest of the spectrum.

Zebrafish Lab @ MacEwan (Building 5 Basement)

Ambient sound level = 46.1 dB


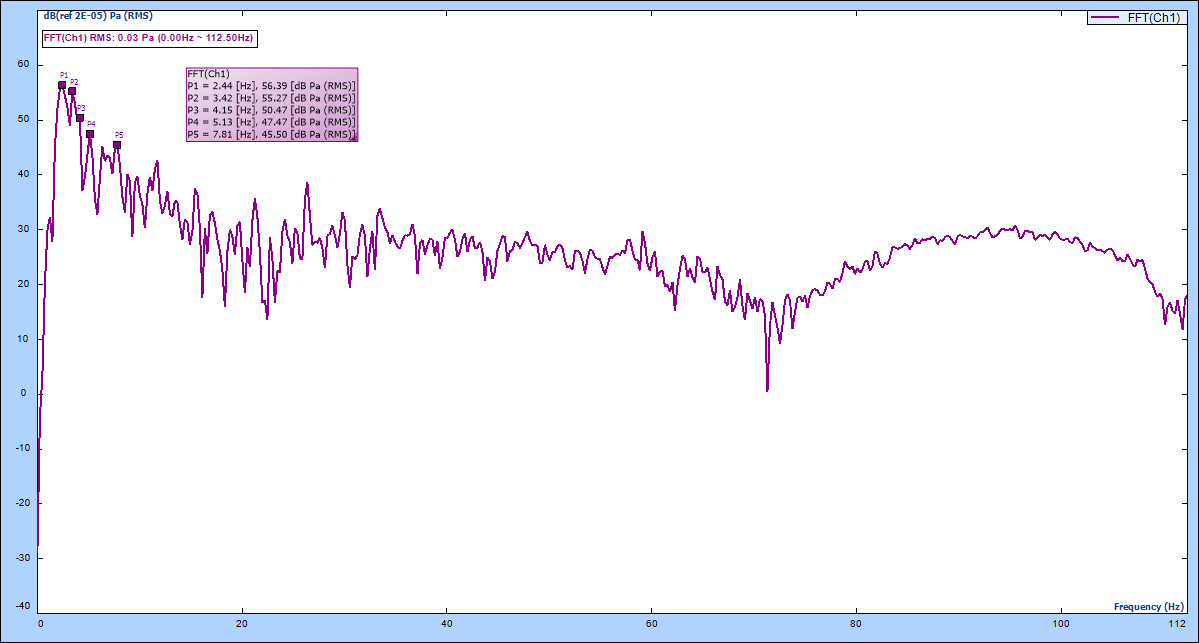


*Note: Not much infrasound present, < 5 Hz is unreliable so the 45.5 dB @ 7.81 Hz is the only significant frequency.*

Some infrasound present

MacEwan Building 5 basement outside west side mechanical room

ASL = 50.8 dB


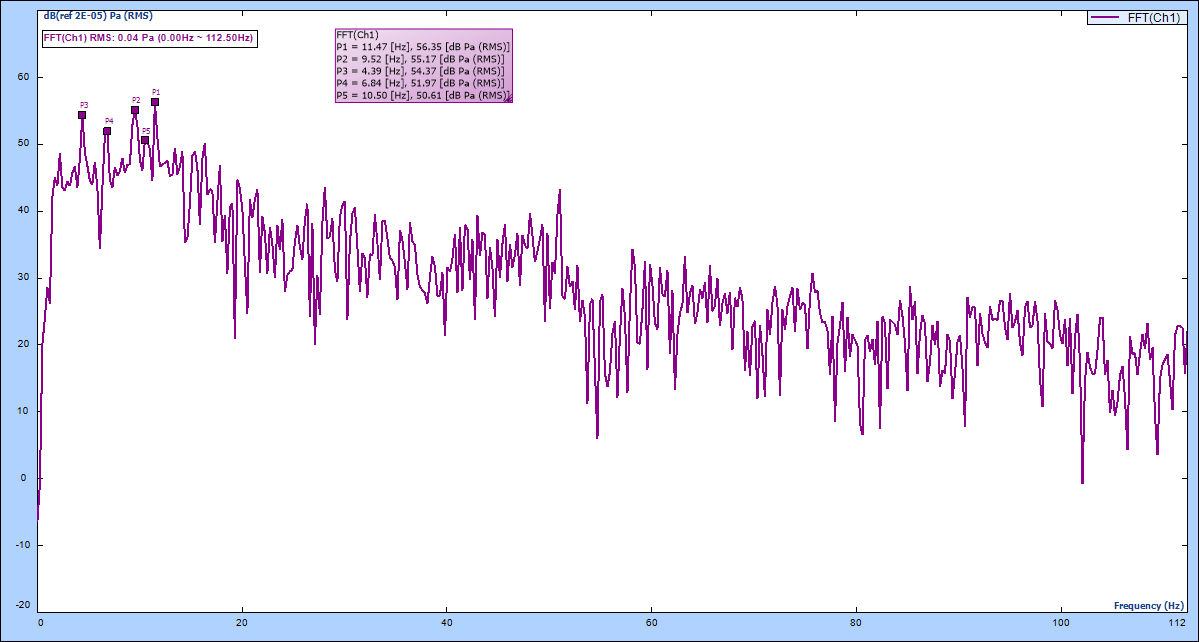


*Note: More going on here for sure. The 9.52, 10.5, and 11.47 Hz are all significant and even higher than the ambient sound level.*

Infrasound present

MacEwan University Library – Building 6 Floor 2 (Common Area)

ASL = 49.8 dB


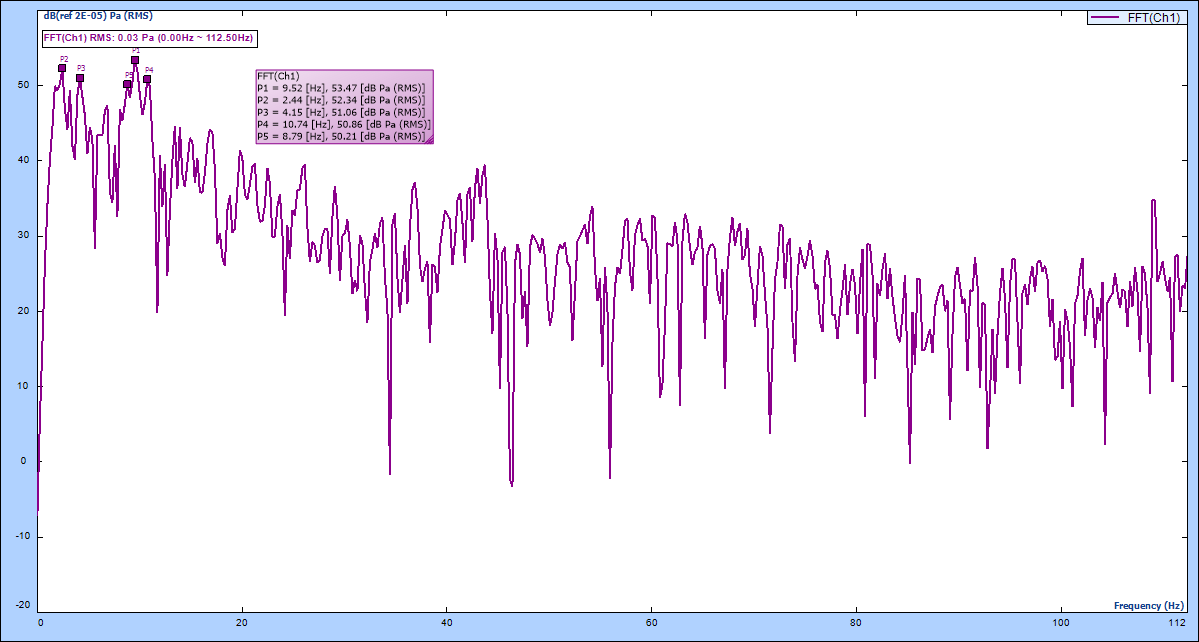


*Note: Some interesting peaks @ 8.79, 9.52, & 10.74 Hz but a bit quieter than the mechanical room and not as loud.*

Some infrasound present

MacEwan University Library – Building 7 Floor 3 (Silent Area)

ASL = 49.7 dB


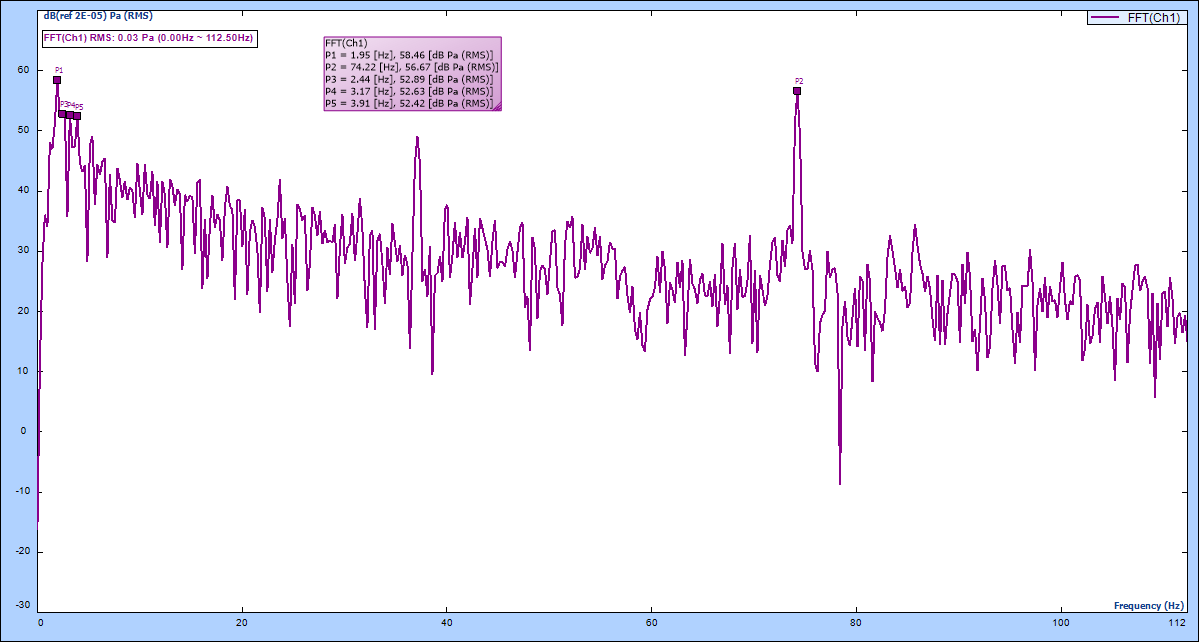


*Note: All peaks are below 5 Hz of above 20 Hz so there is nothing going on here for infrasound.*

No infrasound present

104^th^ St Bus Stop Between SAMU building & Robbins building (Heavy Traffic Area)

ASL = 68.9 dB


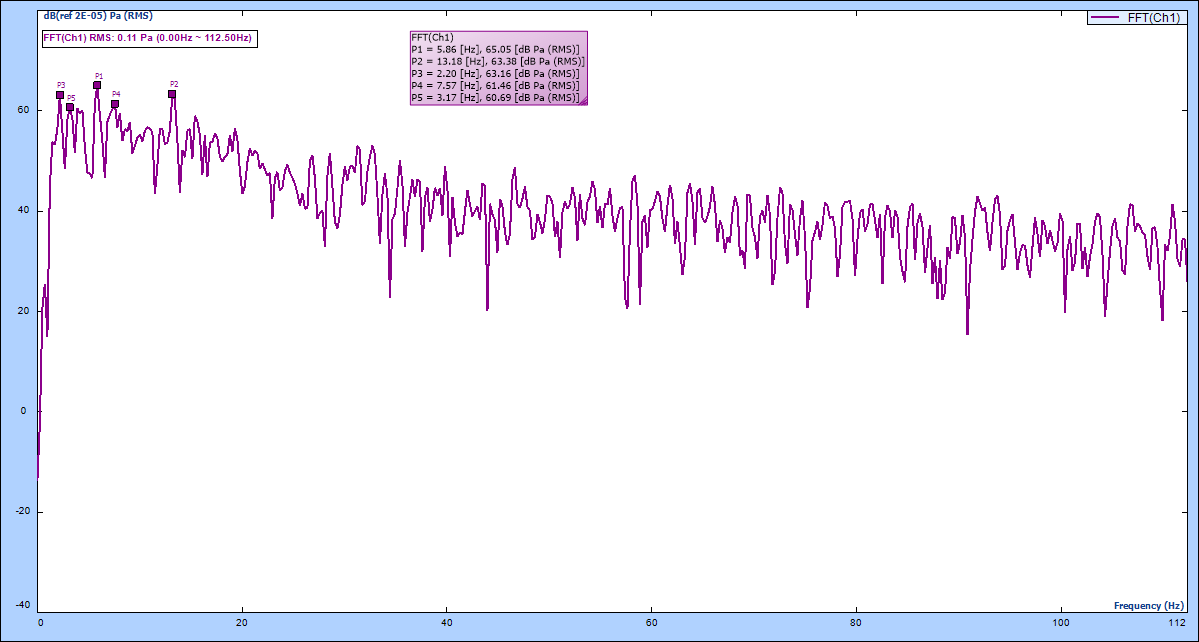


*Note: Busses and LRT line nearby. Louder peaks shown at 7.57 and 13.18 Hz. Interestingly well below the ambient sound level – should investigate what this means.*

Infrasound present

Construction site on 104 ave in front of MacEwan campus

ASL = 72.2 dB


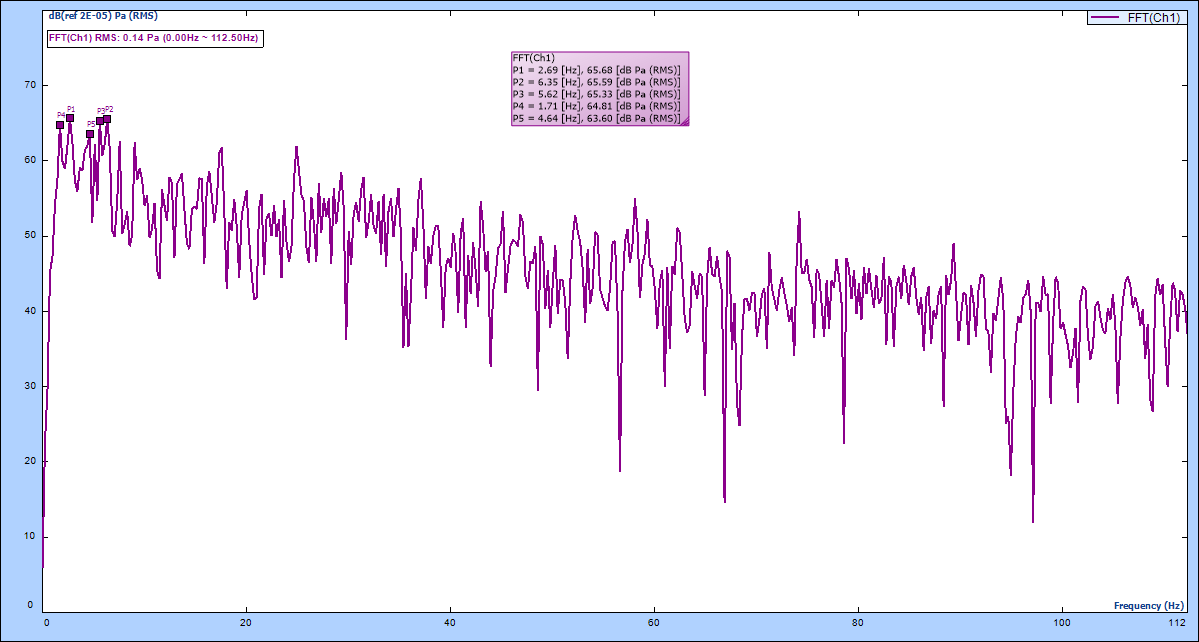


*Note: Strangely enough, only a notable peak at 6.35 Hz. Everything ~5 and below is unreliable. I was expecting infrasound here for sure but it should be noted that there was only 1 piece of heavy equipment running at the time. Everything else was hand tools and power tools.*

Little infrasound present

**Day 2 – April 2, 2023 – University Symphony Orchestra @ The Winspear Centre – 10:30 am to 1 pm**

Before dress rehearsal (10:30 am)

ASL: 44.6 dB


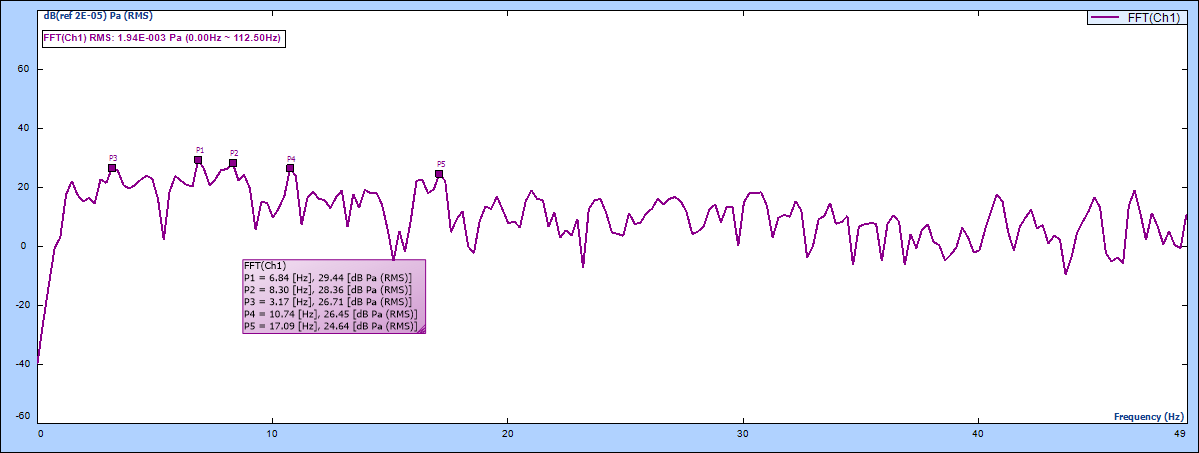


*Note: A bit of chatter in the background, but nothing that should have interfered. Seems to be some natural infrasound @ ~10 Hz at ~25 dB.*

Little infrasound present (could have been due to nearby LRT station)

During start of dress rehearsal (10:30 am)

ASL:87 dB


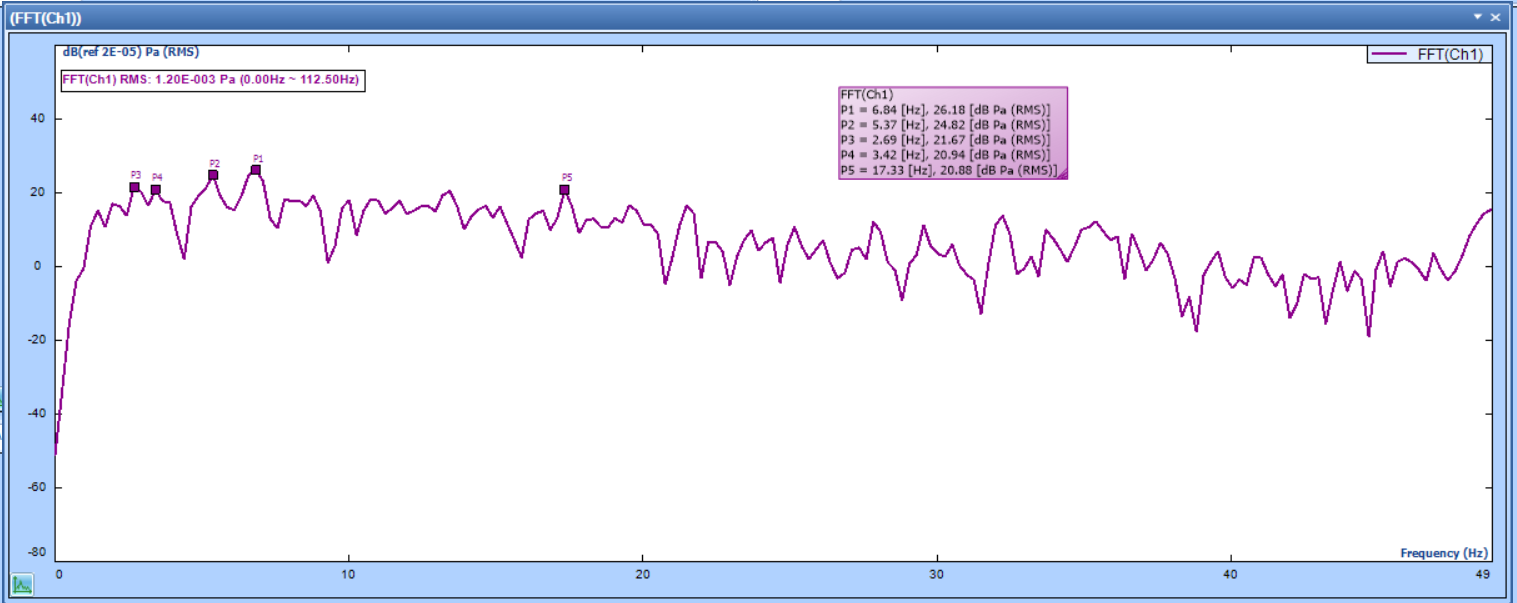


*Note: When lots of strings by themselves there is some infrasound @ ~17 Hz (~20 dB) (1^st^ song)*


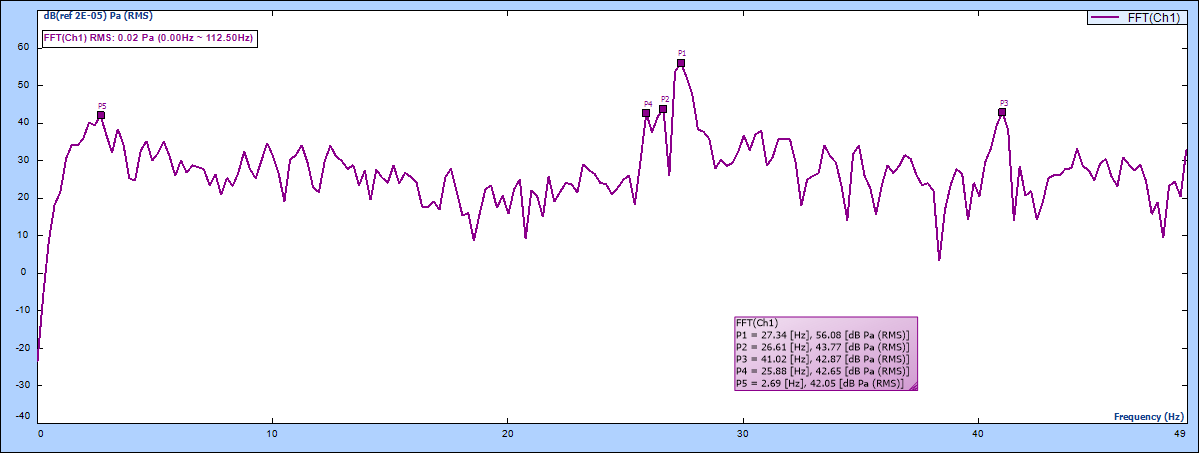


*Note: Lots of bass instruments and brass. Surprisingly no infrasound. (19 min. in; ASL = 84.9 dB).*

*
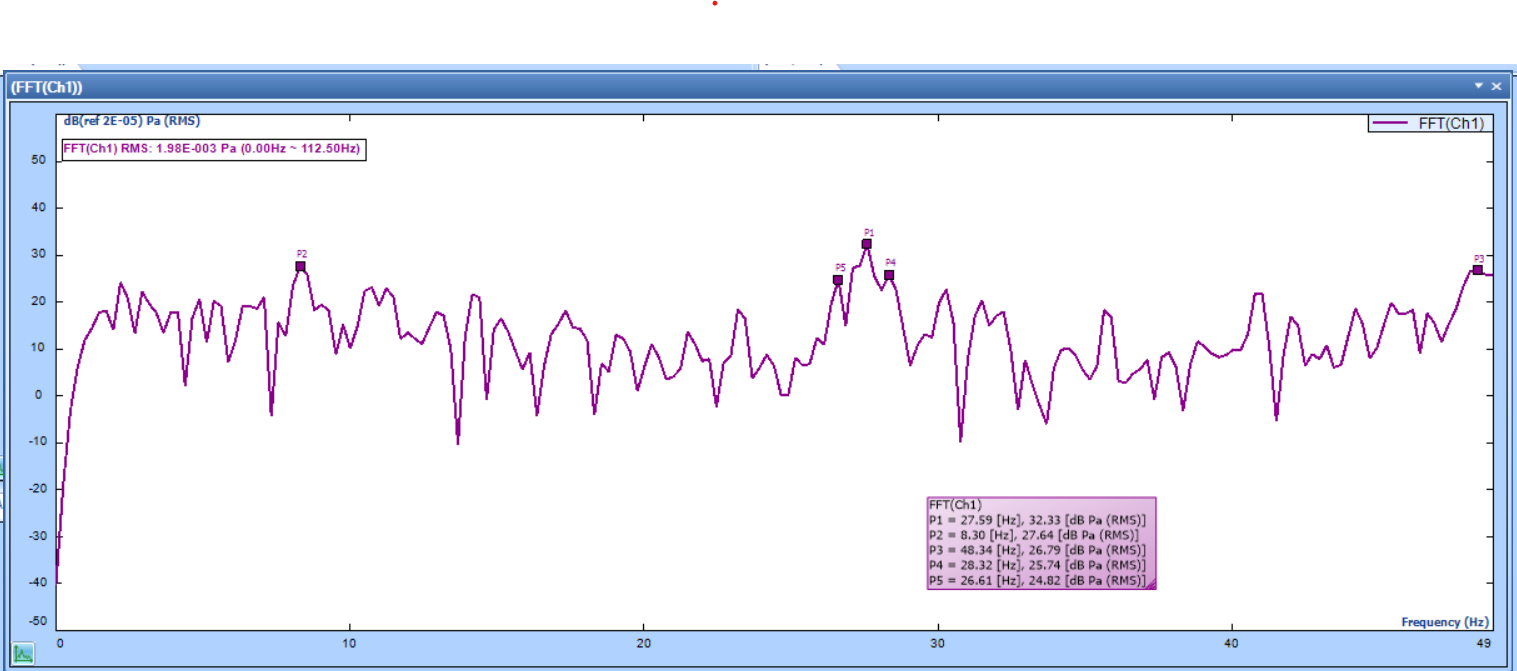
*

*Note: Bit of a spike when it was strings and woodwinds only (22 min. in; 2^nd^ song)*

*
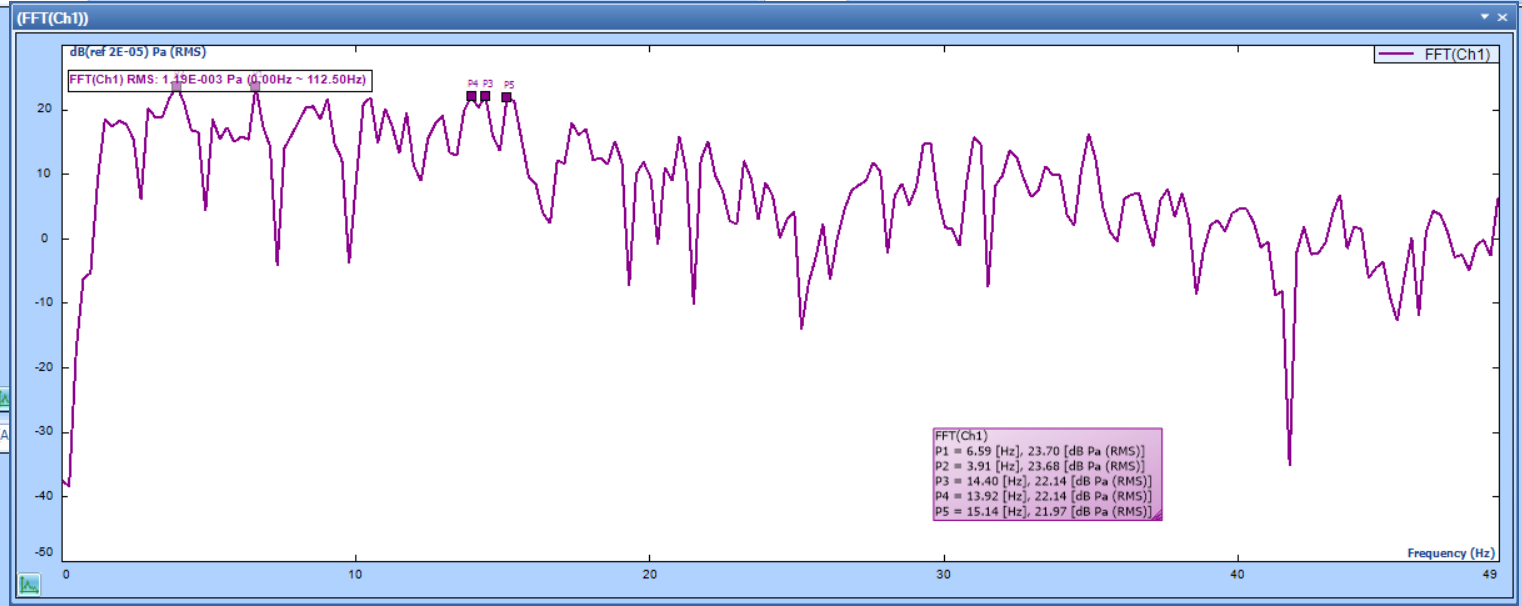
*

*Note: Lots of infrasound while lots of strings playing. Strangely higher notes (3^rd^ song; ASL = 82.2 dB)*

*
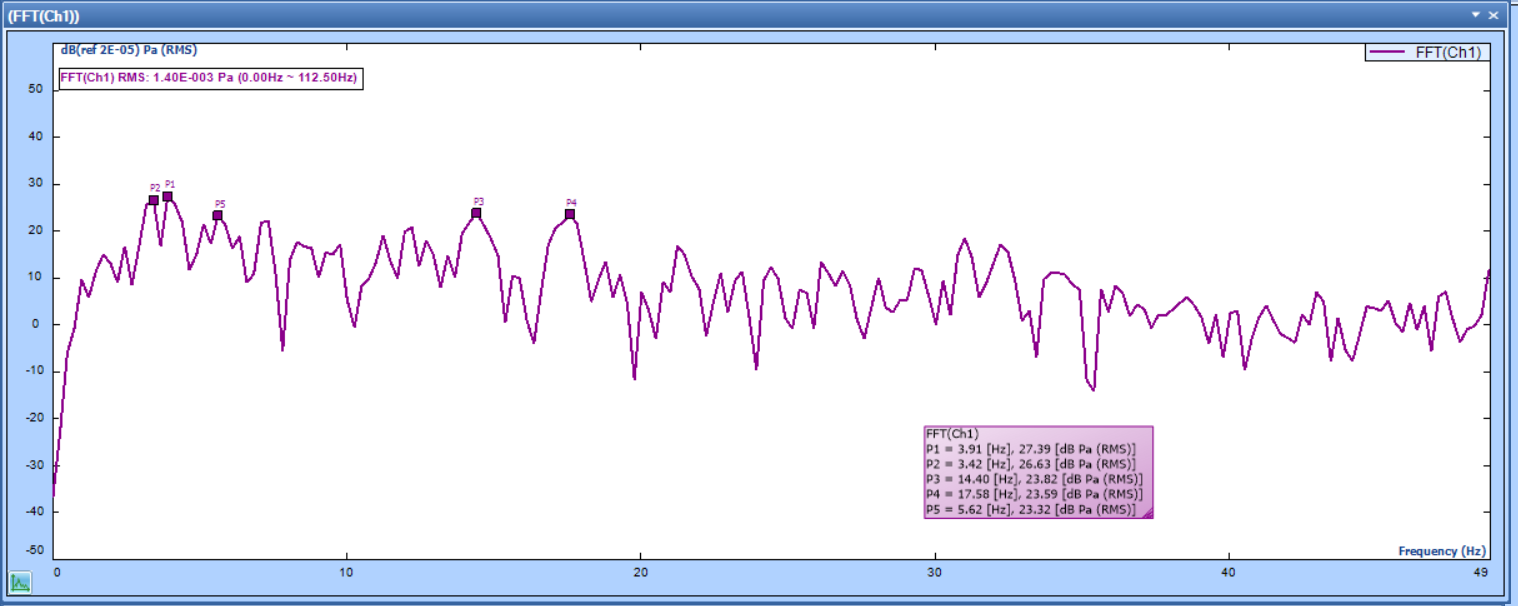
*

*Note: 3^rd^ song; lots of strings and very abrupt low brass. Lots of infrasound (ASL = 83.7 dB)*

*
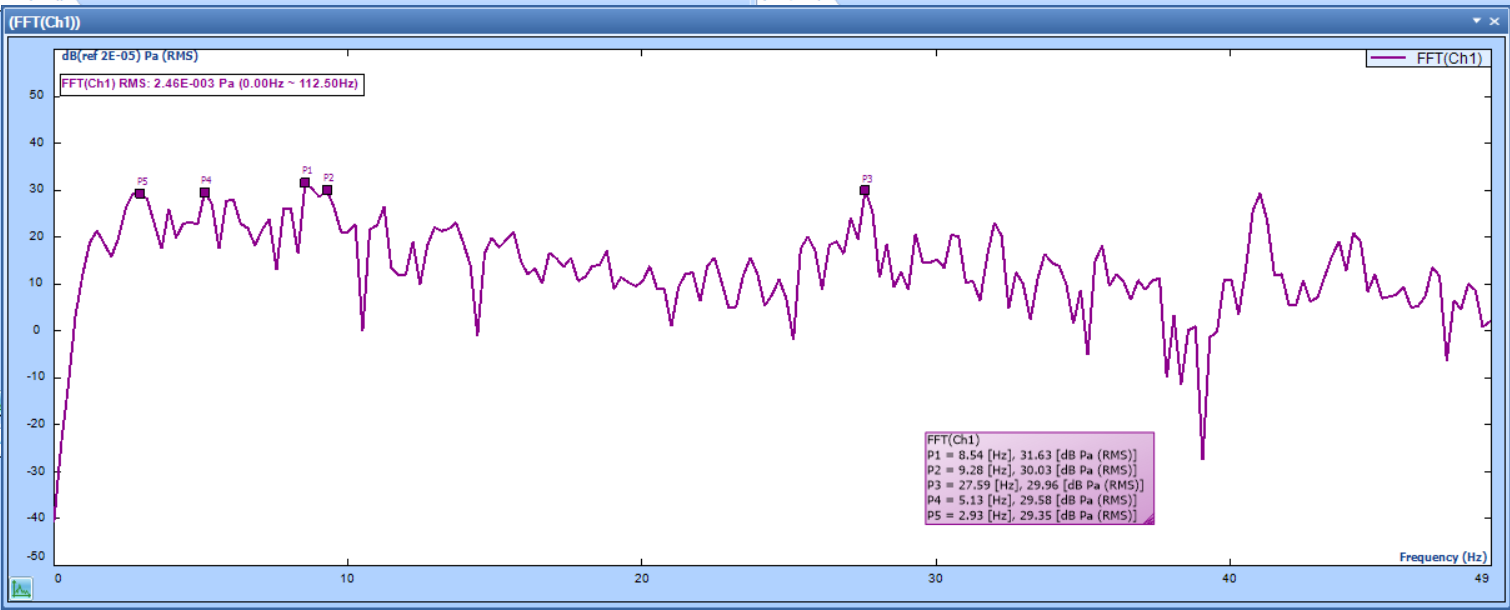
*

*Note: 4^th^ song(?) @ 34 min. in. Lots of strings and high piccolo. Decent infrasound @ 8.5 to 9.5 Hz (ASL = 66.8 dB).*

*
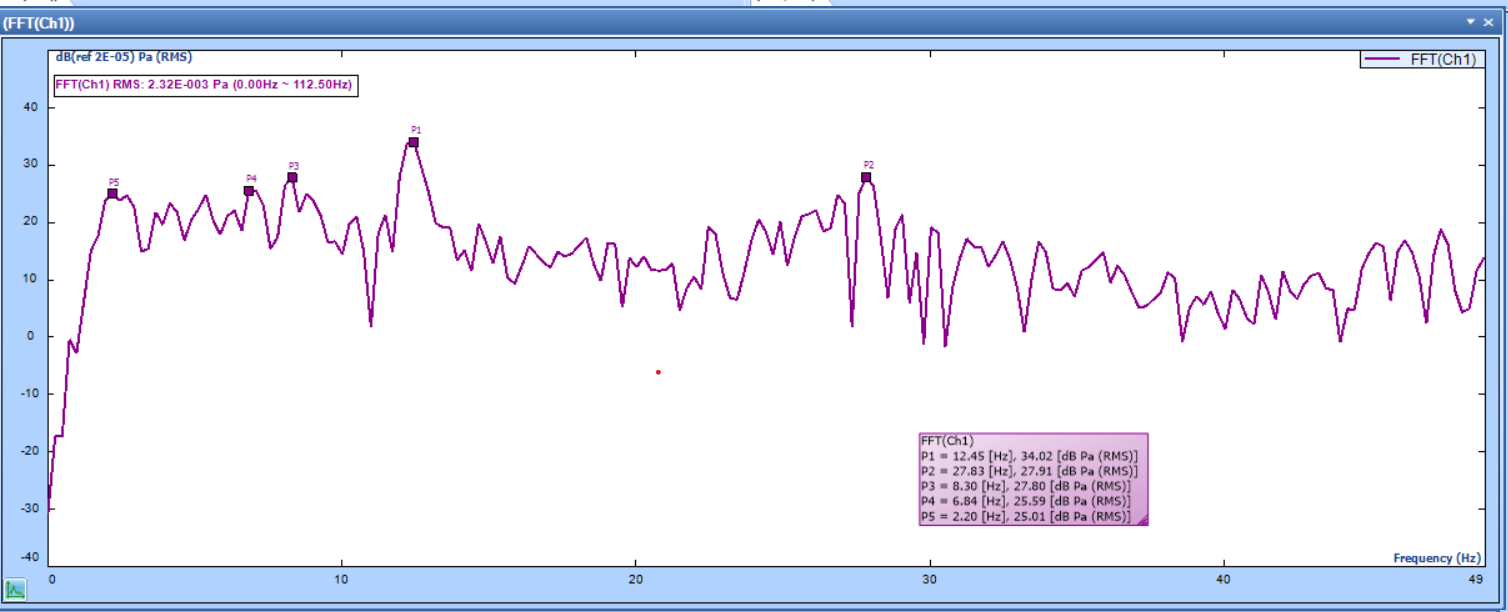
*

*Note: 12 Hz spike between 4^th^ and 5^th^ song when they were stomping their feet*

*
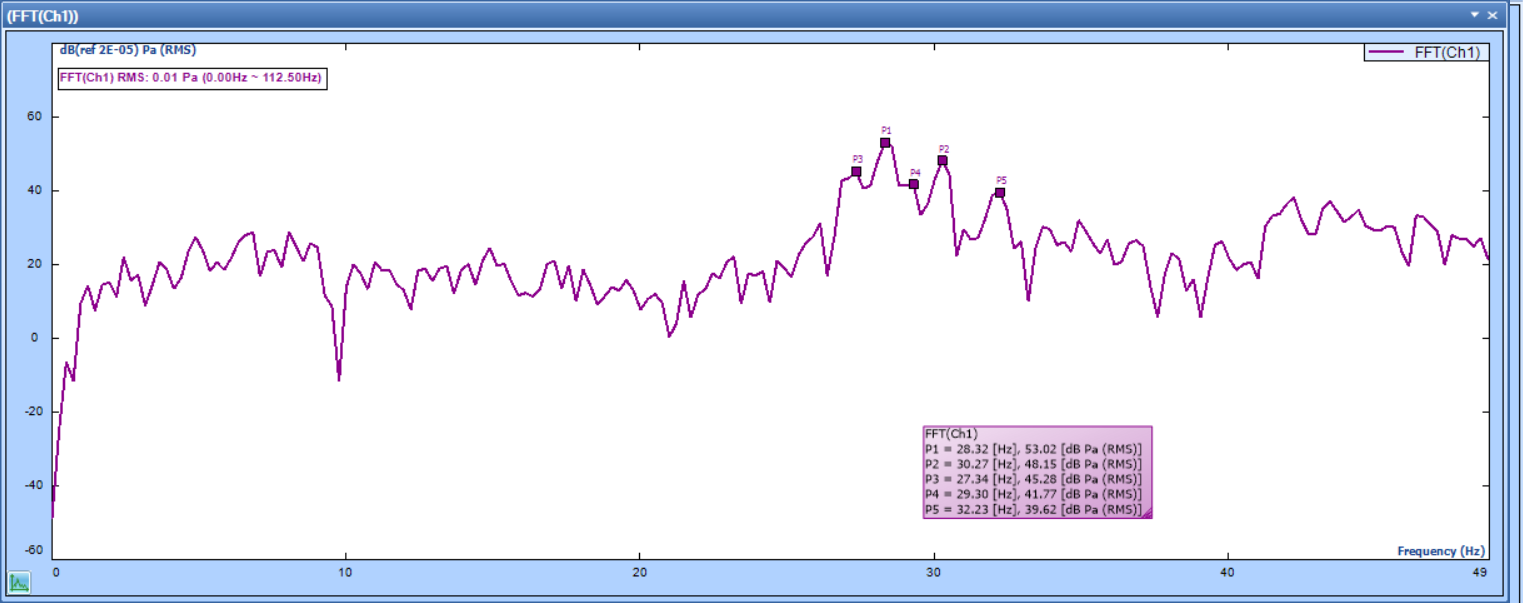
*

*Note: 5^th^ song during a brass and percussion heavy part. Surprisingly no distinct infrasound. No strings present. (ASL = 86.9 dB)*

*
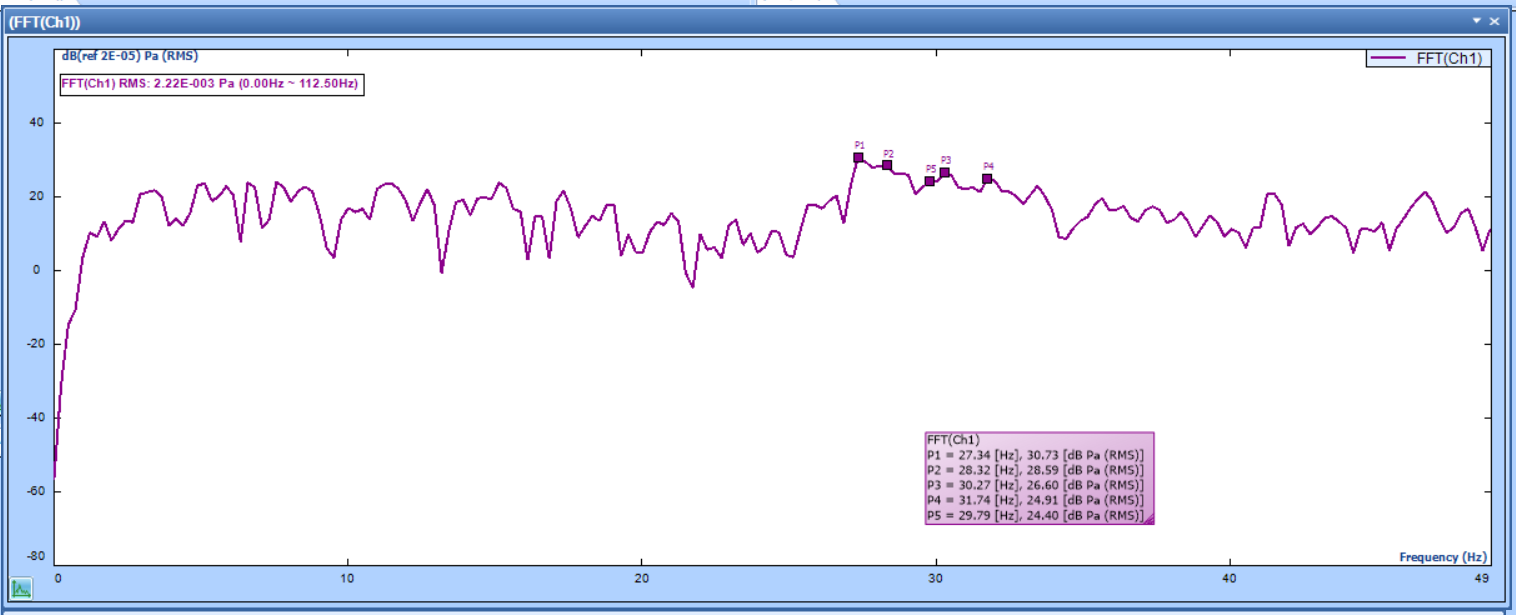
*

*Note: Nothing significant when just conductor talking @ 11:55 am.*

*
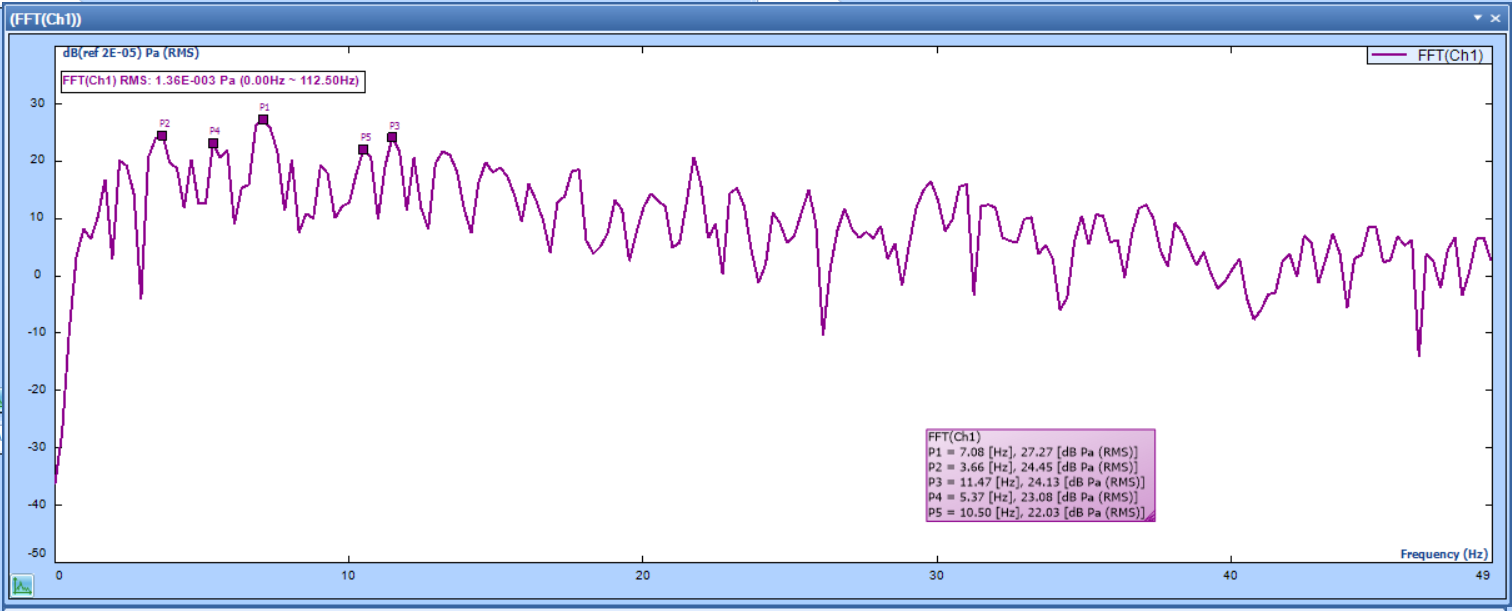
*

*Note: Definitely more present in the strings (2^nd^ last song; mainly strings; ASL = 71.8 dB)*

*
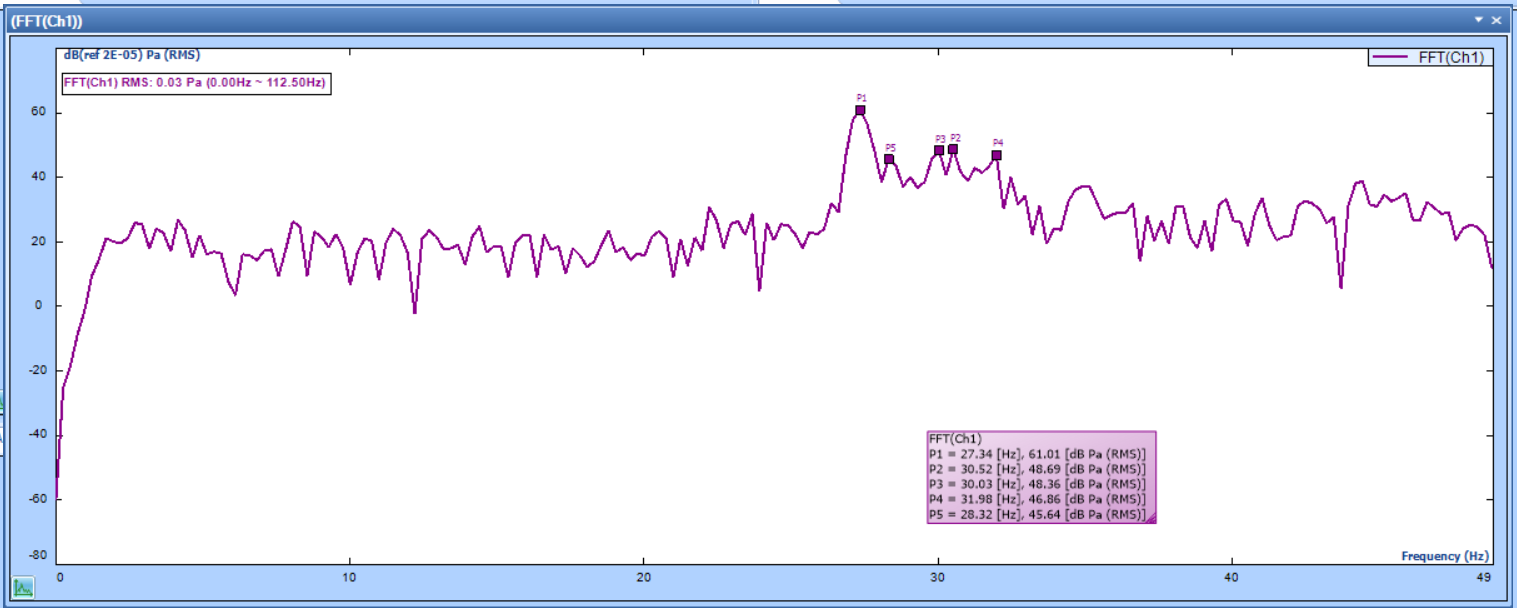
Note: Again, not very present in brass/woodwind/percussion parts (12:07 pm; ASL = 73.2 dB)*

*
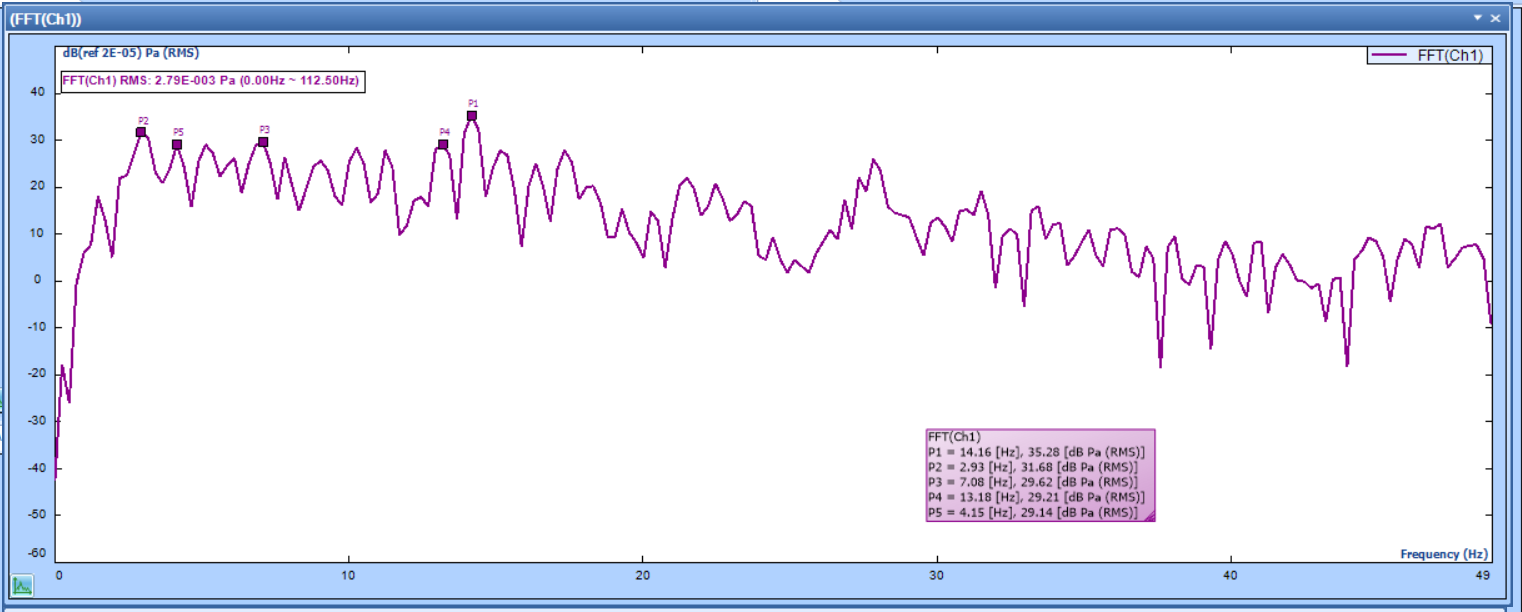
*

*Note: Between playing @ 12:10 pm. When everyone starts moving and shuffling there are more spikes. More of a mechanical vibration?*

*
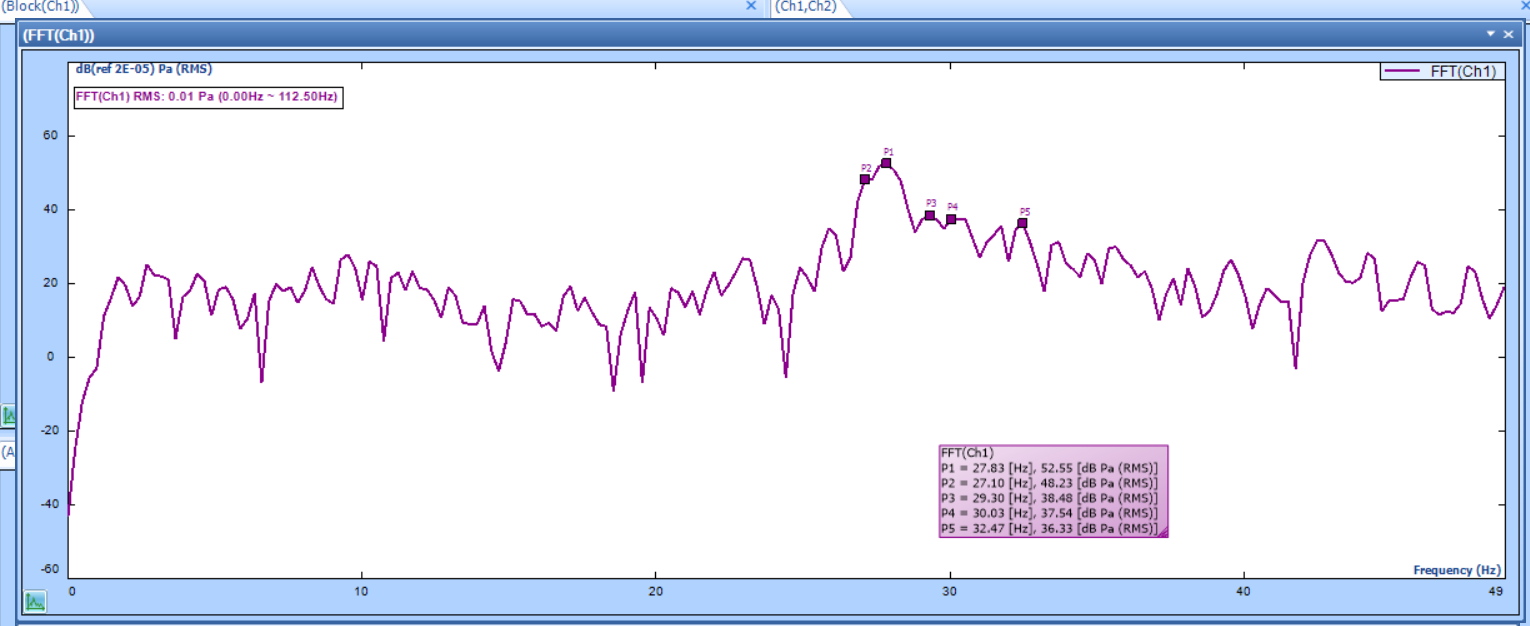
*

*Note: Again. Nothing while just conductor talking. ASL = 40.9 dB*

*
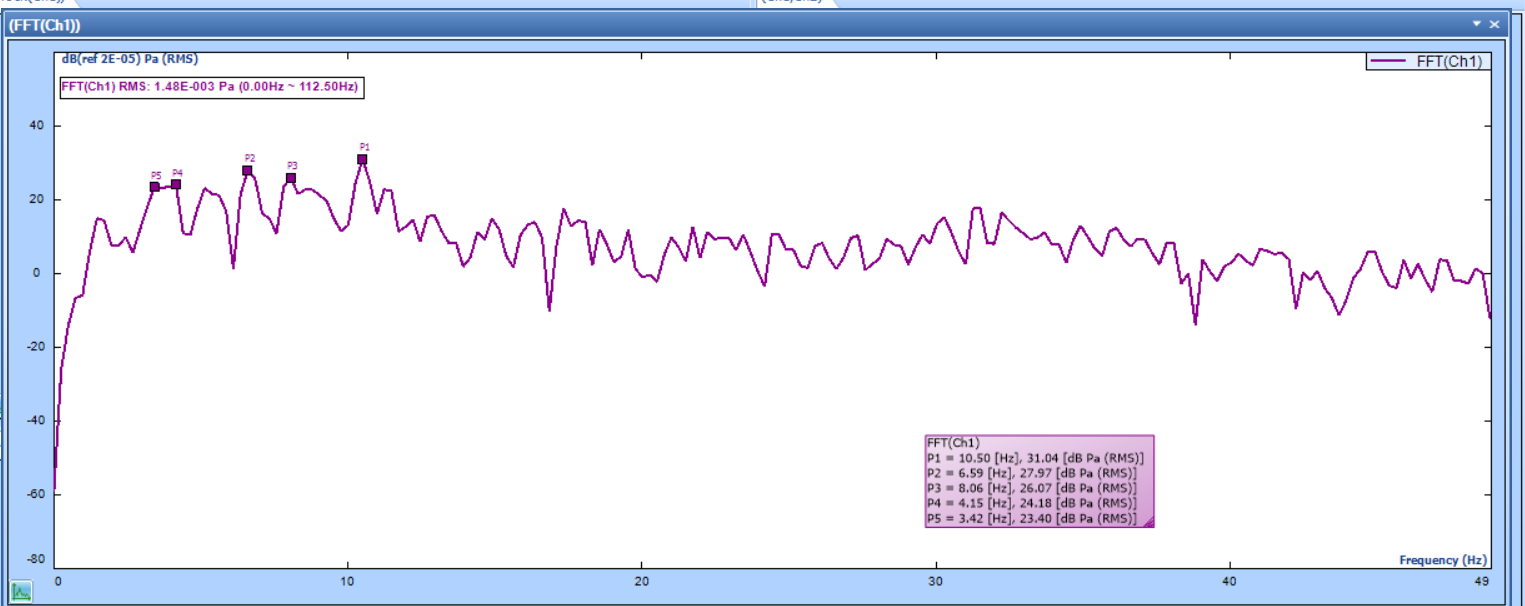
*

*Note: A little bit of 10 Hz in the Oboes/Clarinets? (ASL = 68.9 dB)*

*
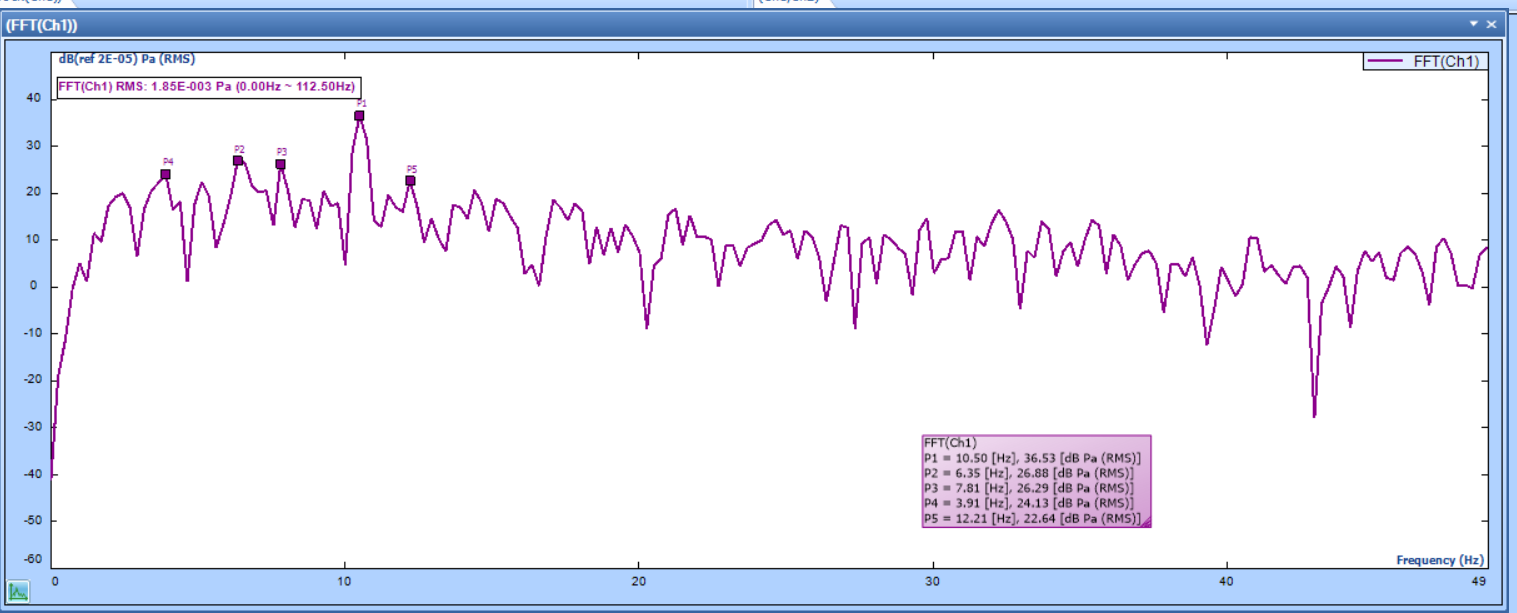
*

*Note: Again, spikes during shuffling.*

*
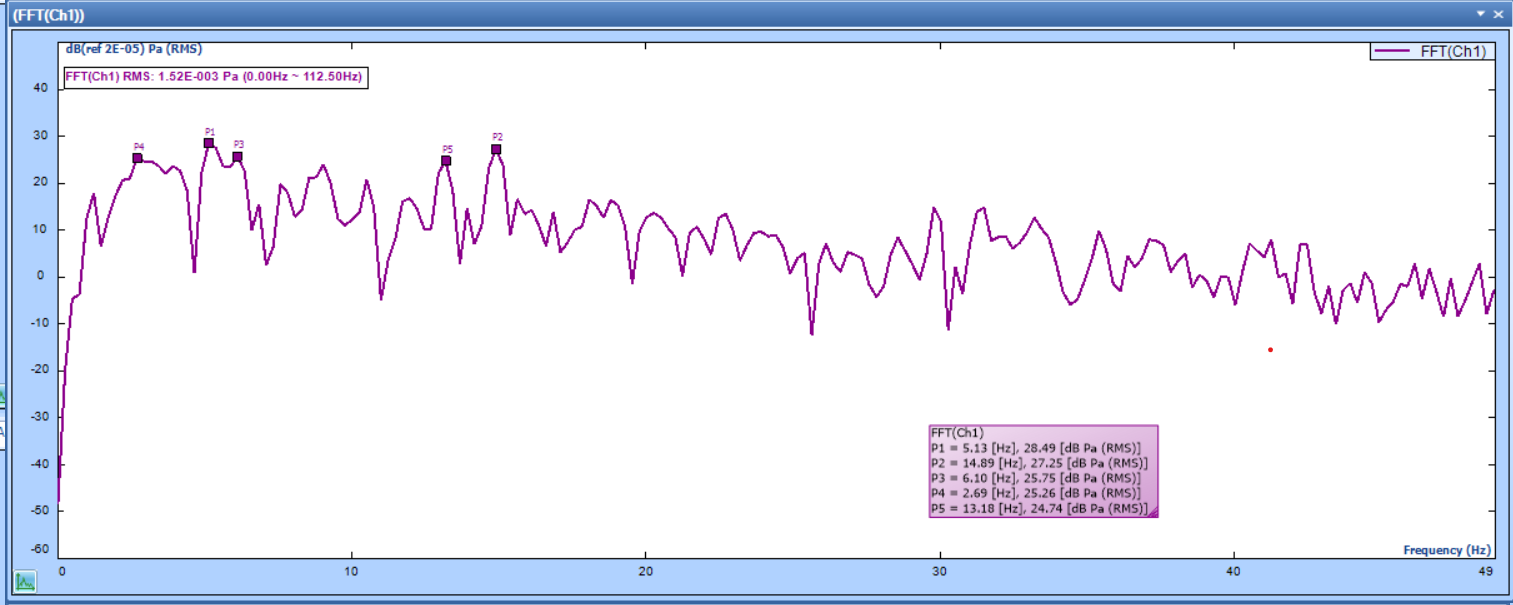
*

*Note: Some spikes w/ just strings and vocalists (ASL = 84.1 dB) Last song.*

*
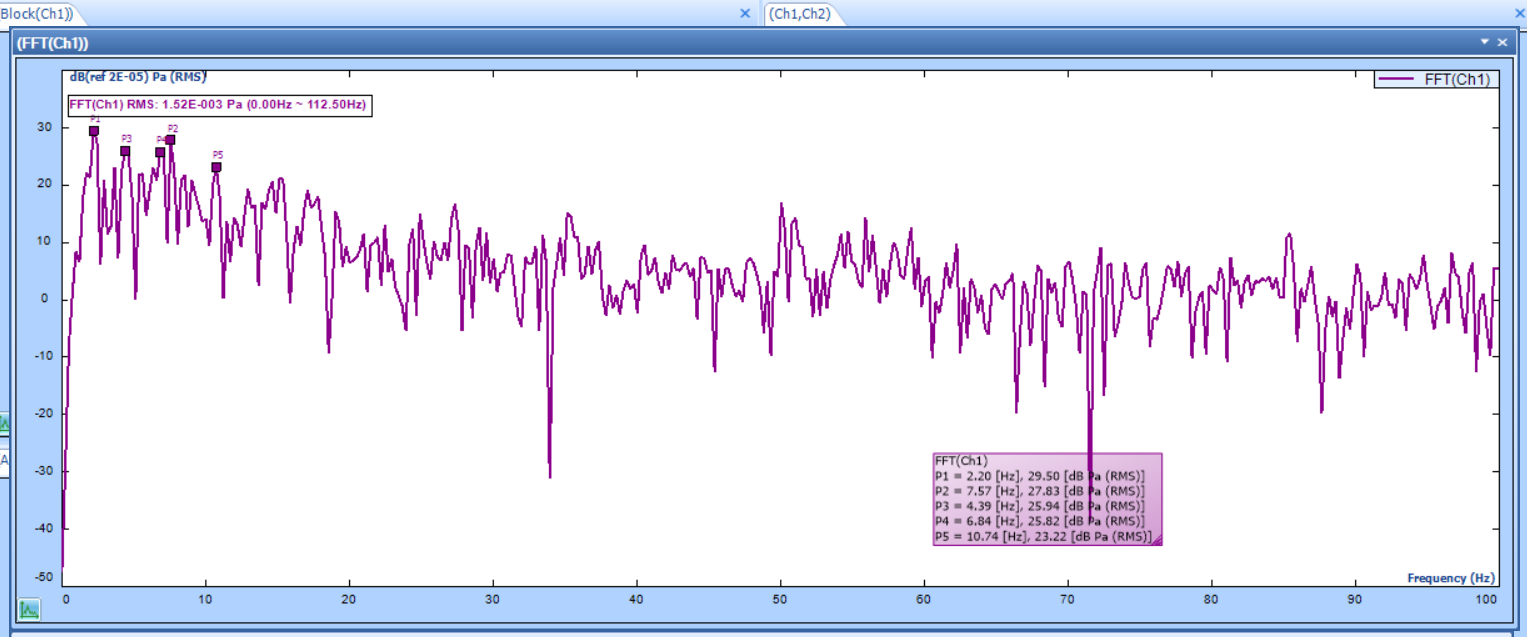
*

*Note: After playing. Just minor chatter. Seems to be some natural 10 Hz to the building. Could be nearby transportation. ASL = 43.6 dB.*

*⸫*

Large amount of body movement from musicians = some infrasound present

Orchestra playing with significant string presence = infrasound present

Orchestra playing with significant woodwind presence = little infrasound present

Orchestra playing with significant brass & percussion presence = no infrasound present

**Day 2 – April 2, 2023 – Rural Recordings – Lac St Anne County - ~15 km West of Onoway in the Forest – 7 to 8:30 pm**

ASL = 26.7 dB


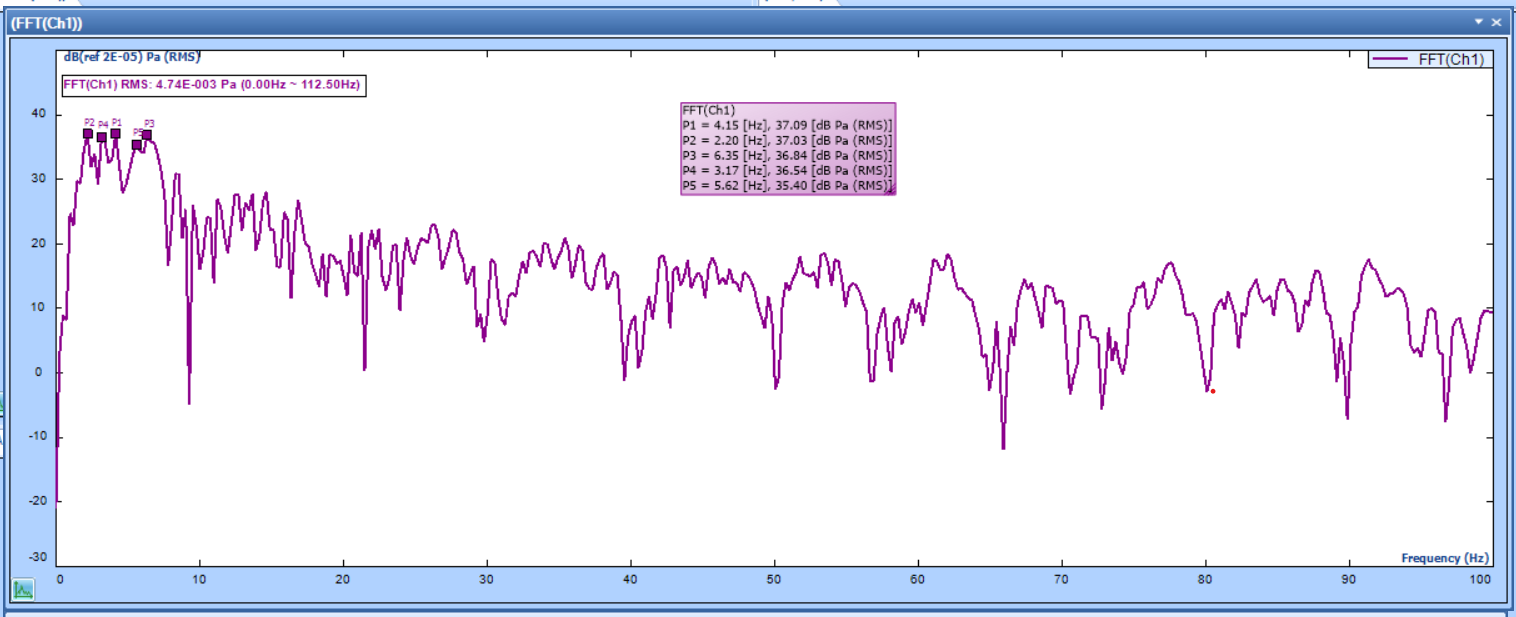


*Note: Some slight peaking @ 6.35 Hz, but close to 5 Hz threshold (7:10 pm)*


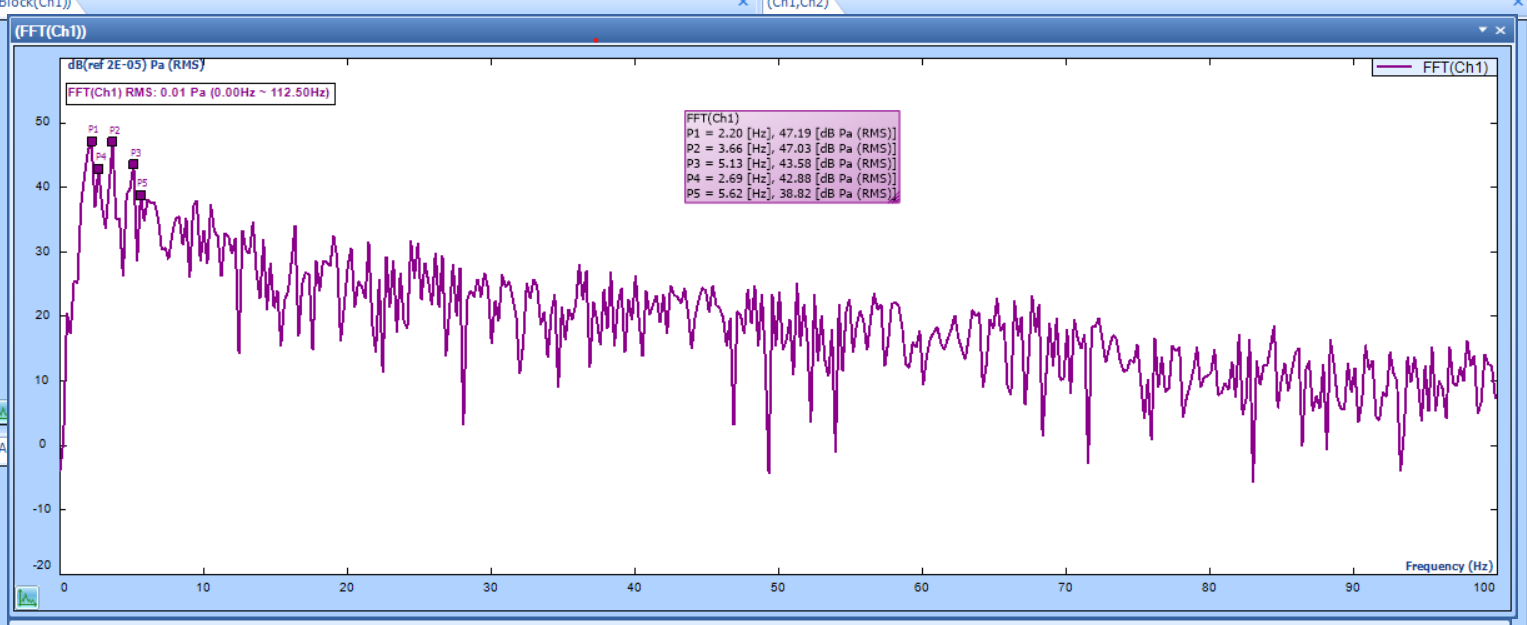


*Note: No significant infrasound peaks (7:30 pm)*


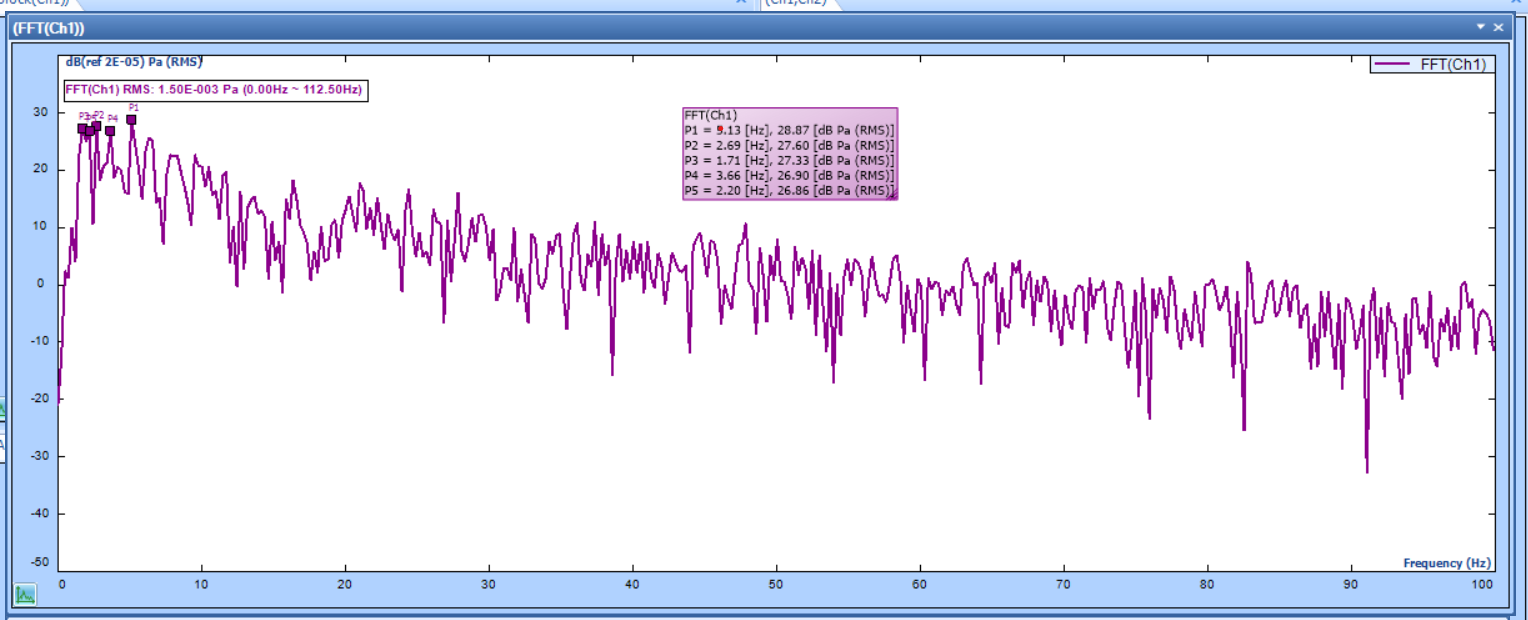


*Note: Still no notable infrasound peaks (7:50 pm)*


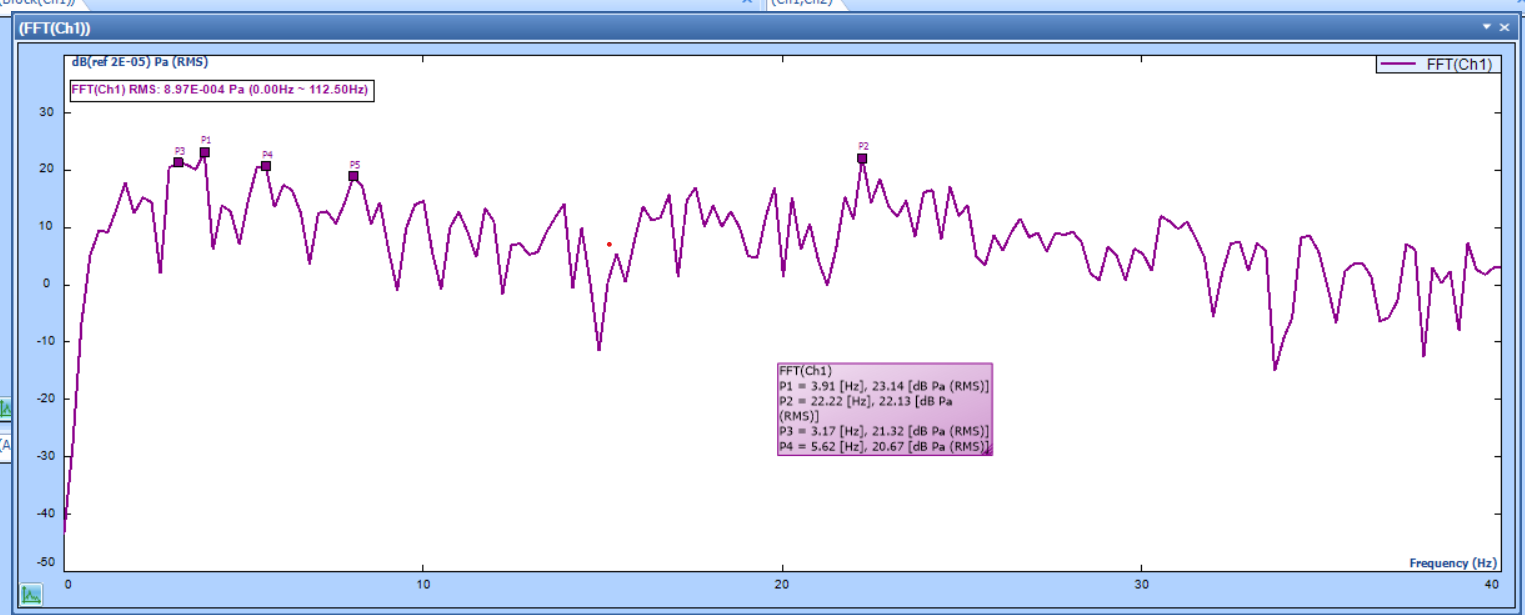


*Note: Light snow began so I switched to a narrower x-axis to filter out the ambient noise. No significant infrasound peaks present (8:05 pm).*


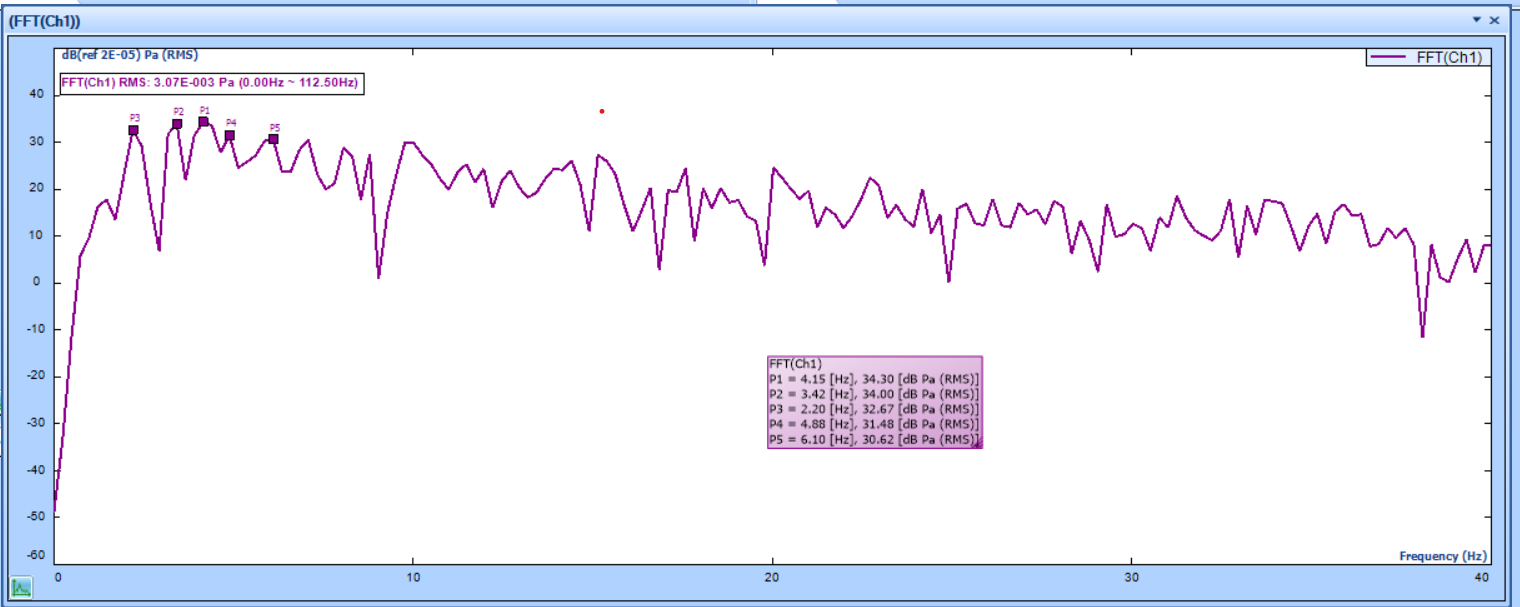


*Note: Still snowing lightly. Slight 6.10 Hz peak but again likely not indicative of significant infrasound (8:25 pm).*

Little infrasound to no infrasound present (the ~6 Hz here and there was questionable).

**Day 3 – Live Band Performance (Heavy Metal) – Grand Mal Rehearsal Studio @ 118 Ave & 142 st., Edmonton, AB – 6 to 8 pm**

Before playing

ASL = 44.1 dB


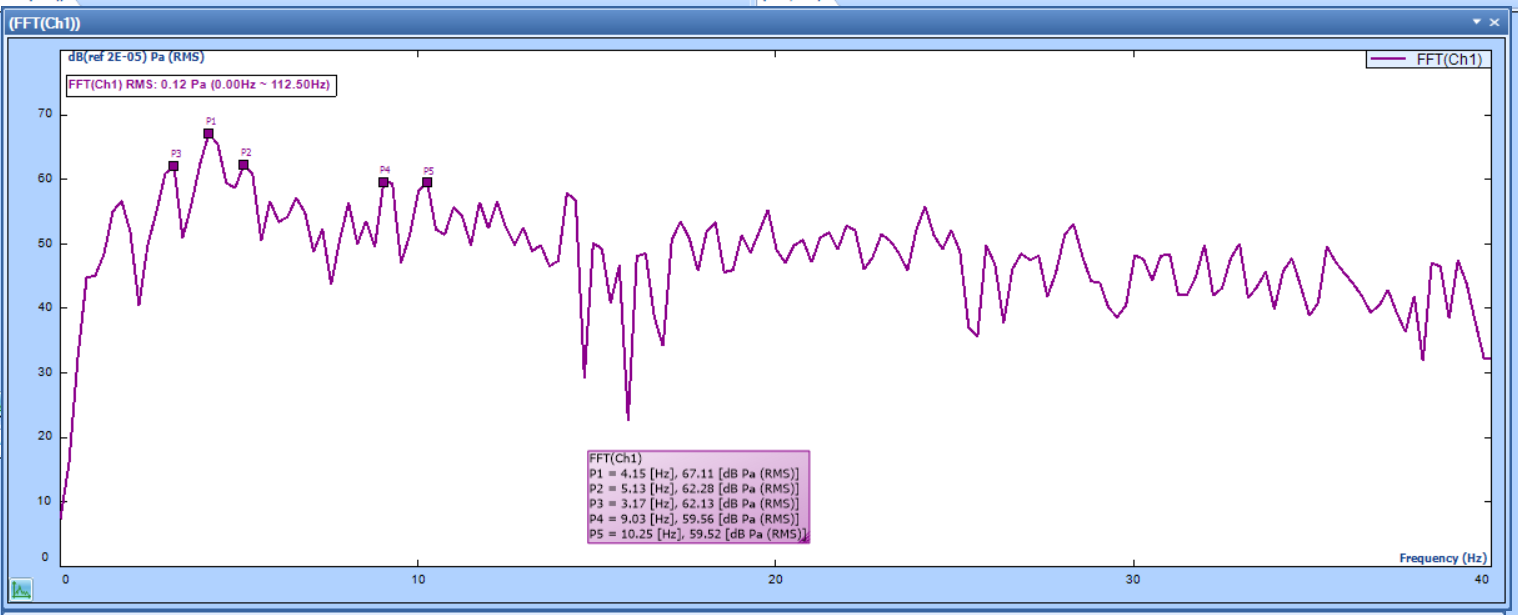


*Note: Some infrasound present @ 9.03 and 10.25 Hz naturally. Could be due to the older structure of the building or the traffic and nearby CN Rail depot.*

While playing

ASL = 111.9 dB


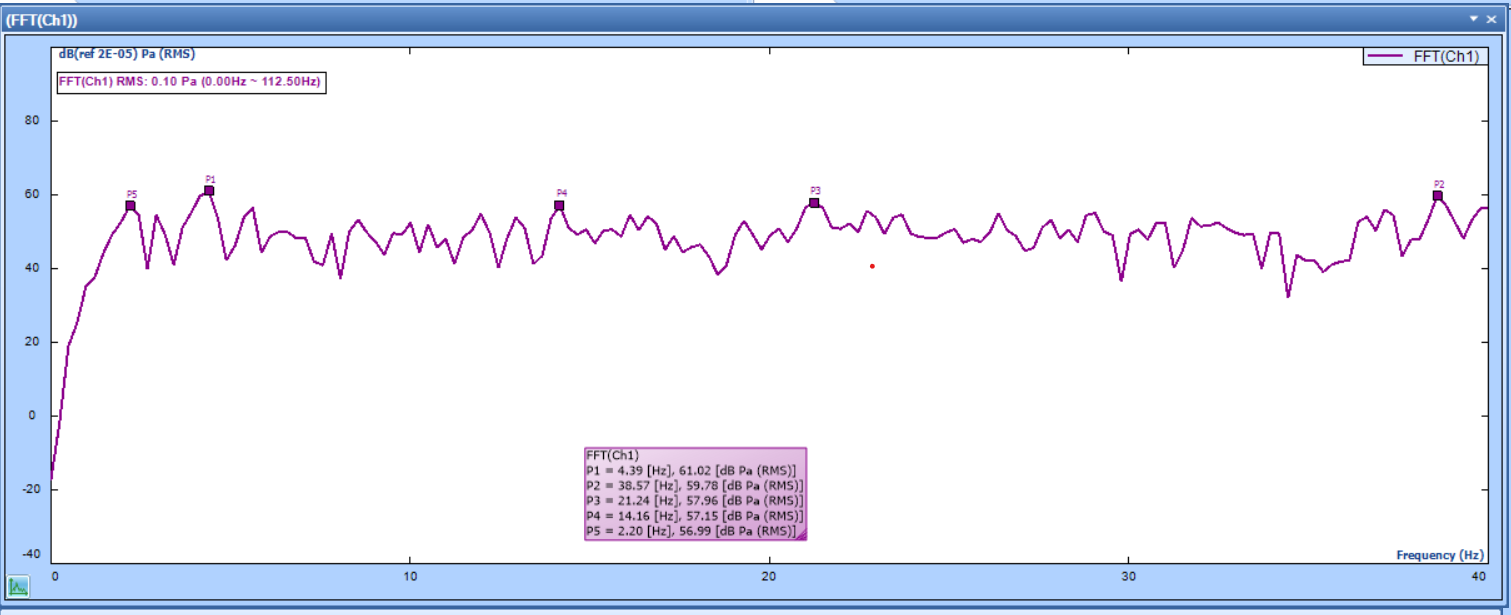


*Note:* Big jump in overall amplitude with an infrasound peak @ 14.16 Hz. Not visually too different from the rest of the spectrum though, so could be questionable.

Little infrasound to some infrasound present.

****END OF FIELD RECORDINGS**

Some evidence of infrasound was found within human environments. Mechanical systems appear to be prime sources as well as urban traffic, whereas construction sites and quieter upper floors of urban buildings don’t seem to show as much.

During performance, the USO appeared to show greatest amounts of infrasound when a lot of strings were playing, some infrasound when woodwinds were playing and when musicians were all moving around, and no infrasound when mainly brass and percussion was playing. The Winspear itself may have some infrasound present due to nearby traffic, but the infrasound found while the orchestra was playing appeared to be distinct from this.

As expected, rural forested areas showed virtually no significant infrasound, supporting the proposal that infrasound is a much bigger environmental pollutant in urban centres.

The heavy metal band performance yielded some less clear results due to some infrasound already being present in the building. The infrasound that was found during performance, however, was distinct from the baseline infrasound levels.

**Infrasound Testing Room Validation Notes**

*NOTE: Crystal Instruments service representatives have informed us that this microphone may not be appropriately sensitive to frequencies between 0-5 Hz. All peaks seen in the below figures @ 5 Hz and below should be considered unreliable and can be ignored.*

**Infrasound Off**

Ambient Noise Level:

Center = 39.6 dB

Left Room = 41.5 dB

Right Room = 40.7 dB


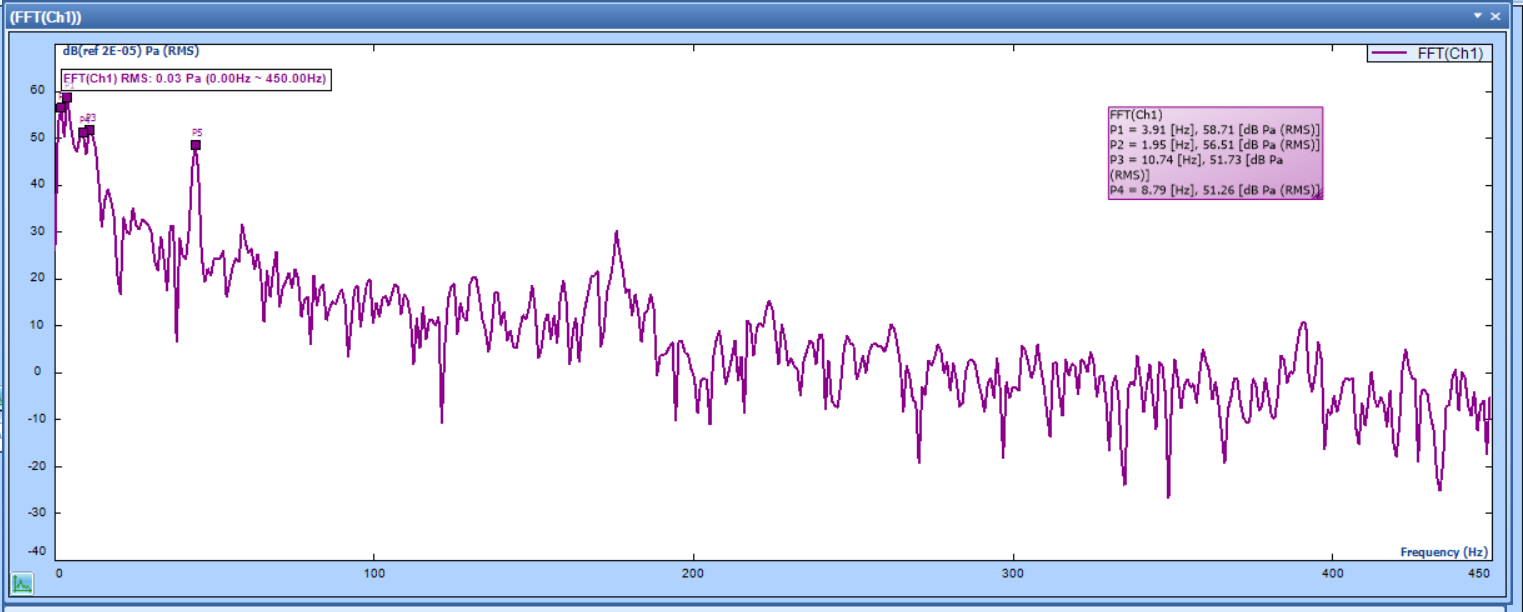


Natural peak at ~10 Hz. Consistent and doesn’t seem to change or go away. About 49-51 dB so we will have to note it but ensure our target frequency amplitudes are clearly more present. 10 Hz is likely coming from something mechanical in the room, but we likely won’t be targeting frequencies this low in the first place so it should be acceptable.

**Infrasound On**

Ambient Noise Level:

Center = 44.6 dB

Left Room = 41.5 dB

Right Room = 42.0 dB


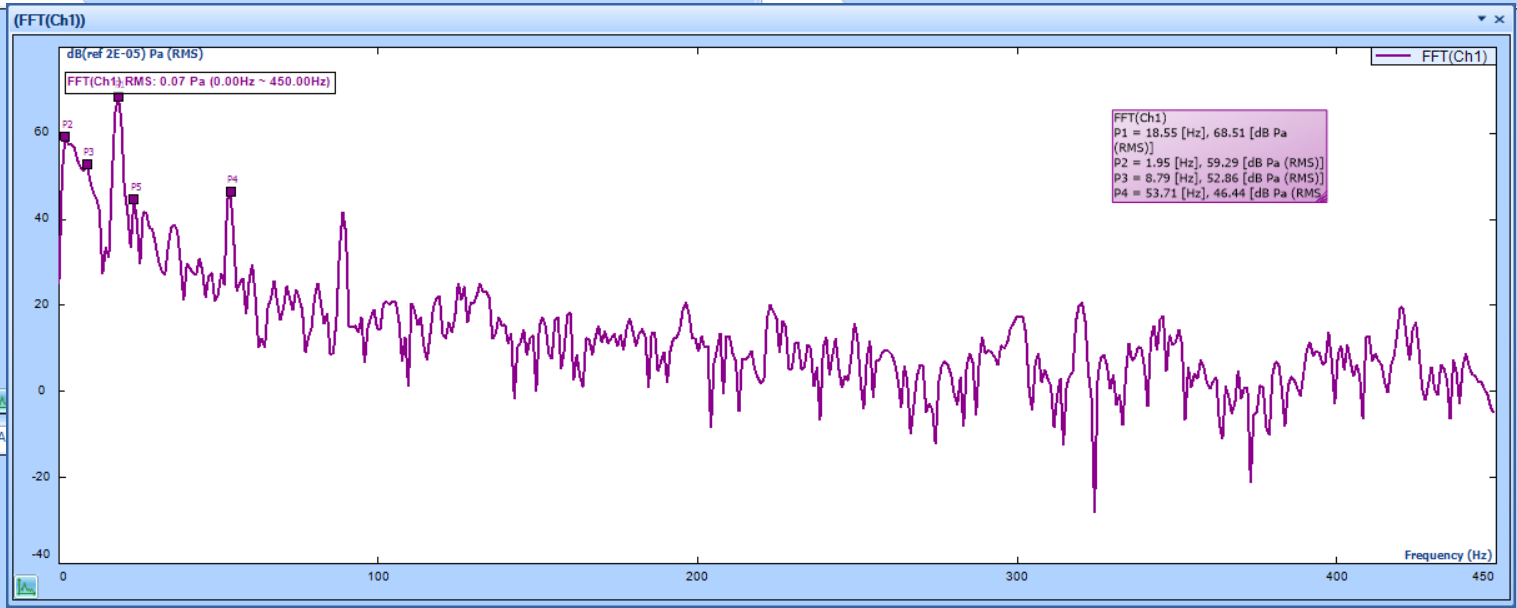


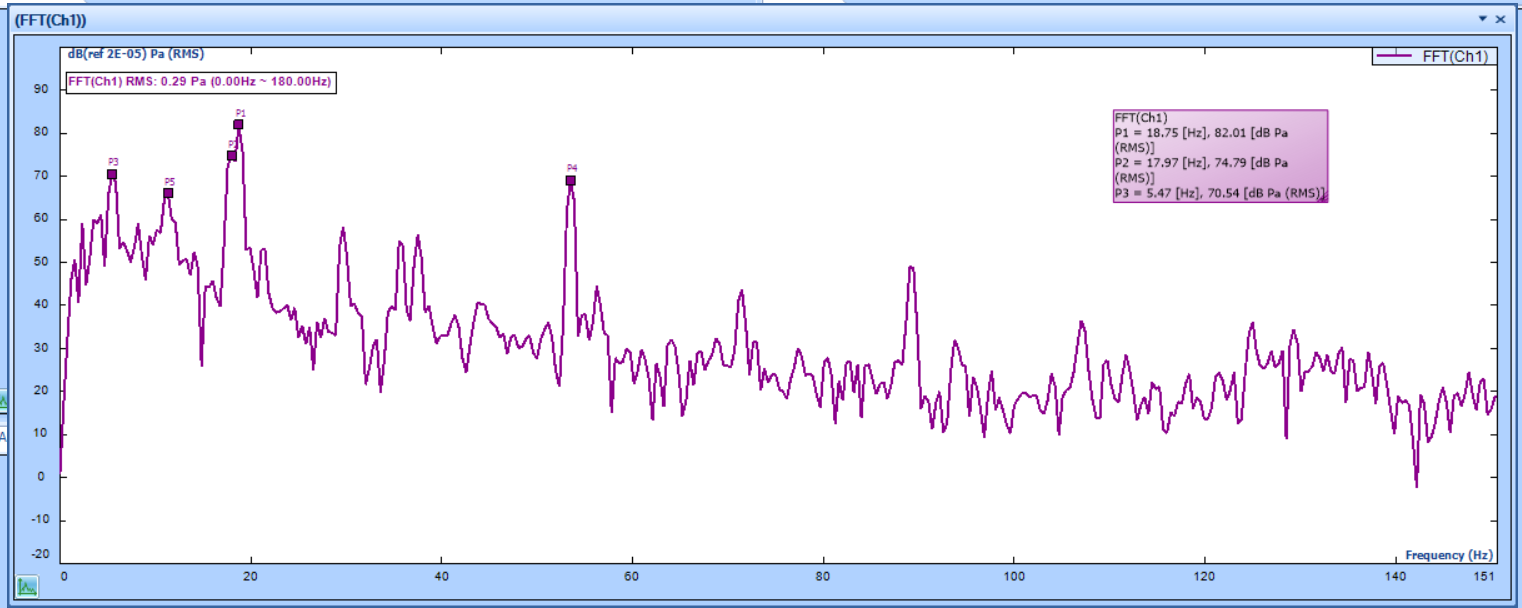


Consistent peaks at ~18 Hz between 75-82 dB. 15 Hz and lower was inconsistent and max of ~66 dB (not loud enough). Range will be set between 15-18 Hz.

**Speakers**

Frequency: Setpoint = 18 Hz; Actual = ~15-18 +/- 0.5 Hz

Small Speaker Amplitude to white line

Large speaker amplitude to pencil line

*Amplitude of infrasound measured to be within 0-2 dB between both rooms. This will be appropriately similar for the study.

**Self-Report Survey (Qualtrics)**

Infrasound and Human Behaviour Study 2023

Survey Flow

Standard: CONSENT (3 Questions)

Branch: New Branch

If

If Do you hereby agree to participate in the above-described research and understand that consent do... I do not consent Is Selected

EndSurvey:

Standard: Consent Done (2 Questions)

Standard: Demographic Information (5 Questions)

BlockRandomizer: 1 - Evenly Present Elements

Standard: Unsettling Song (2 Questions)

Standard: Calming Song (2 Questions)

Standard: Q2 (PANAS After) (3 Questions)

Standard: Clip Description (3 Questions)

Standard: Clip Feeling (3 Questions)

Standard: Q5 (2 Questions)

Standard: Finished (1 Question)

Standard: Participant # (4 Questions)

| Page Break |  |
| --- | --- |

Start of Block: CONSENT

Consent Timer Timing

First Click (1)

Last Click (2)

Page Submit (3)

Click Count (4)

Consent Instructions Please read the consent form on your desk and indicate whether or not you consent  to participation in this study as described.

**After you have signed** the document or indicated you do not consent, please indicate below so your response can be recorded electronically as well.

Consent Do you hereby agree to participate in the above-described research and understand that consent does not constitute a waiver of legal rights in the event of research-related harm?

- I consent (1)
- I do not consent (2)

Skip To: End of Survey If Consent = 2

End of Block: CONSENT

Start of Block: Consent Done

Before Sample Timer Timing

First Click (1)

Last Click (2)

Page Submit (3)

Click Count (4)

Consent Done Thank you for your consent to participate in this study. You may now provide your preliminary saliva sample as described to you by your experimenter and as outlined by the cheat sheet by your saliva collection kit.

If you have any questions on how to complete this, please indicate to your experimenter that you need help.

Once your sample has been provided, please ensure it is sealed and placed back in the appropriate position on the desk. You may then proceed with the survey and go to the next question.

End of Block: Consent Done

Start of Block: Demographic Information

Demographic Timer Timing

First Click (1)

Last Click (2)

Page Submit (3)

Click Count (4)

Age What is your age?

________________________________________________________________

Gender What is your preferred gender?


Rich Content Editor.

- Female (23)
- Male (24)
- Non-binary (25)
- Other (26)
- Prefer Not to Answer (27)

Sex What was your sex assigned at birth?

- Male (4)
- Female (5)
- Other (6)
- Prefer Not to Answer (7)

Medication Are you currently taking any prescribed medication for a mood or anxiety disorder?

- Yes, I am taking medication for an anxiety disorder (4)
- Yes, I am taking medication for a mood disorder (e.g., depression, bipolar) (5)
- Yes, I am taking medication for both disorders (6)
- No, I am not on either medication currently (7)

End of Block: Demographic Information

Start of Block: Unsettling Song

Unsettling Song Please play the below music clip and listen for the next 5 minutes while remaining seated. Infrasound may or may not be present during the duration of the listening period.

NOTE: If you are not the only participant, please before starting, tell the researcher that you are ready and wait for them to tell you both to start.

Unsettling Confirm Please confirm that you could hear music/sound playing.

- Yes, I heard the audio (6)
- No, I did not hear anything (7)

End of Block: Unsettling Song

Start of Block: Calming Song

Calming Song Please play the below music clip and listen for the next 5 minutes while remaining seated. Infrasound may or may not be present during the duration of the listening period.

NOTE: If you are not the only participant, please before starting, tell the researcher that you are ready and wait for them to tell you both to start.

Calming Confirmation Please confirm that you could hear music/sound playing.

- Yes, I heard the audio (6)
- No, I did not hear anything (7)

End of Block: Calming Song

Start of Block: Q2 (PANAS After)

PANAS Timer Timing

First Click (1)

Last Click (2)

Page Submit (3)

Click Count (4)

Q2 (PANAS After) This scale consists of a number of words that describe different feelings and emotions. Read each item and then list the number from the scale below next to each word. Indicate to what extent you feel this way right now, that is, at the present moment.

Q2 (PANAS After) After listening to the music clip, rate how you feel right at this current moment.

|  | Very Slightly (56) | A little (57) | Moderately (58) | Quite a bit (59) | Extremely (60) |
| --- | --- | --- | --- | --- | --- |
| Interested (46) |  |  |  |  |  |
| Distressed (47) |  |  |  |  |  |
| Excited (48) |  |  |  |  |  |
| Upset (49) |  |  |  |  |  |
| Strong (50) |  |  |  |  |  |
| Guilty (51) |  |  |  |  |  |
| Scared (52) |  |  |  |  |  |
| Hostile (53) |  |  |  |  |  |
| Enthusiastic (54) |  |  |  |  |  |
| Proud (55) |  |  |  |  |  |
| Irritable (56) |  |  |  |  |  |
| Alert (57) |  |  |  |  |  |
| Ashamed (58) |  |  |  |  |  |
| Inspired (59) |  |  |  |  |  |
| Nervous (60) |  |  |  |  |  |
| Determined (61) |  |  |  |  |  |
| Attentive (62) |  |  |  |  |  |
| Jittery (63) |  |  |  |  |  |
| Active (64) |  |  |  |  |  |
| Afraid (65) |  |  |  |  |  |

End of Block: Q2 (PANAS After)

Start of Block: Clip Description

Clip D Timer Timing

First Click (1)

Last Click (2)

Page Submit (3)

Click Count (4)

Q3 Instructions Please respond to the following questions by indicating which number most suits you on a scale from 1 to 9. Please adjust the slider to match your response for each question.

Q3 In general, I would say that the music clip was:

|  | Strongly Disagree | Neutral | Strongly agree |
| --- | --- | --- | --- |

|  | 1 | 2 | 3 | 3 | 4 | 5 | 6 | 7 | 7 | 8 | 9 |
| --- | --- | --- | --- | --- | --- | --- | --- | --- | --- | --- | --- |

| Happy () | 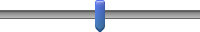 |
| --- | --- |
| Relaxing () | 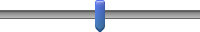 |
| Scary () | 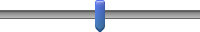 |
| Calming () | 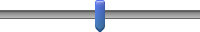 |
| Intense () | 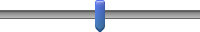 |
| Serious () | 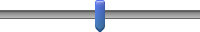 |
| Amusing () | 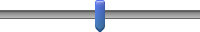 |
| Anxiety-provoking () | 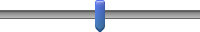 |
| Interesting () | 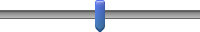 |
| Suspenseful () | 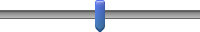 |
| Funny () | 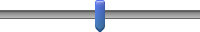 |
| Boring () | 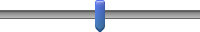 |
| Silly () | 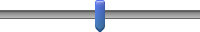 |
| Sad () | 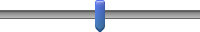 |

End of Block: Clip Description

Start of Block: Clip Feeling

CF Timer Timing

First Click (1)

Last Click (2)

Page Submit (3)

Click Count (4)

Q4 Instructions Please respond to the following questions by indicating which number most suits you on a scale from 1 to 9. Please adjust the slider to match your response for each question.

Q4 While listening to the music clip, I felt:

|  | Strongly Disagree | Neutral | Strongly Agree |
| --- | --- | --- | --- |

|  | 1 | 2 | 3 | 3 | 4 | 5 | 6 | 7 | 7 | 8 | 9 |
| --- | --- | --- | --- | --- | --- | --- | --- | --- | --- | --- | --- |

| Happy () | 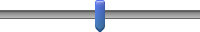 |
| --- | --- |
| Calm () | 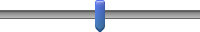 |
| Relaxed () | 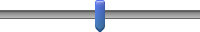 |
| Bored () | 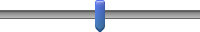 |
| Excited () | 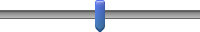 |
| Scared () | 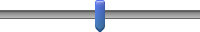 |
| Sad () | 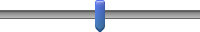 |
| Good () | 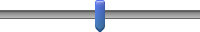 |
| Bad () | 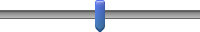 |
| Comfortable () | 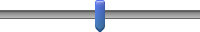 |
| Irritated () | 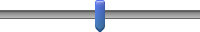 |
| Upset () | 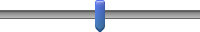 |

End of Block: Clip Feeling

Start of Block: Q5

Infrasound Timer Timing

First Click (1)

Last Click (2)

Page Submit (3)

Click Count (4)

Q5 Do you think the infrasound was on while you listened to the clip?

- Yes (1)
- No (2)

End of Block: Q5

Start of Block: Finished

Complete Your participation in this portion of the experiment is now finished. Please please report to the researcher present to complete the last steps of the experiment.

End of Block: Finished

Start of Block: Participant #

| 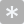 |
| --- |

ID For the researcher present, please enter the password and next indicate the anonymous participant identification number of the participant that has just completed this portion of the experiment.

This response is encoded and cannot be answered by the participant.

The participant identification number is only used to keep record of answers and samples and cannot be traced back to any personal information provided.

ID Please enter the participant identification number. 

Double check that this number is correct and corresponds to the number the participant's saliva samples are labelled with.

________________________________________________________________

On/Off Infrasound Condition

- Infrasound was ON (4)
- Infrasound was OFF (5)

Calming/Unsettling [Researcher Check] To the best of my memory or as I had indicated in my lab notes, I can confirm that this participant was exposed to the:

- Calming Song (4)
- Unsettling Song (5)

End of Block: Participant #

**Researcher Script**

**Infrasound Study v3 Summer 2023**

*Be sure that there are enough copies of all forms*

*Copies of the consent form and credit form should be in front of each computer*

*Pencils should be on desks in 6-340B and 6-340D*

*Ensure that the desks are in the correct place and only one chair is in the room in 6-340B and 6-340D.*

*Ensure that a disposable paper cup, bottle of water, straw, and eppendorf tubes with caps are set up at each desk.*

*Chairs can be moved close to the doors to make sure participants can hear the instructions.*

*Be sure the Chrome browser is open, and click on the bookmarked link to load Qualtrics prior to each study.*

*Check that the frequency generator is not facing toward the participants/rooms.*

[*Go to participants outside of 6-340*]

E: Hi, are you here for the infrasound study? Good. I’m________. Please follow me.

*[lead participants into 6-340, lead each participant into their own room]*

E: You can leave your things anywhere along the wall. If you have a cell phone, please make sure it’s turned off.

[*Experimenter stands in the sightline of both doors -- make sure you are seen and heard by both participants*]

E: Please have a seat and slide both of your chairs up to the doorway, facing me. Can you both hear me okay?

[*Wait for response*]

E: In today’s study, we are specifically looking at the effects of infrasound on how people evaluate things. Do either of you know what infrasound is?

[Wait for response]

E: Infrasound is low frequency sound that is below 20Hz and cannot be heard by humans. Some studies have shown that when people are exposed to infrasound, they may experience different sensations, such as feelings of awe or suspense. Although some studies have reported this, other research has reported that people feel nothing at all. Infrasound is actually pretty common in daily life, and is produced by things like low rumbling pipes and windmills. If you are exposed to infrasound in this study, it is nothing outside of what you would experience in your everyday life.

E: In this study, we’re interested to see if the presence of infrasound impacts how people perceive things – in this case, the perception of different styles of music. So, what will happen is that you will be listening to different types of music. Before listening to each musical piece, you will be asked to fill out a questionnaire and provide a saliva sample. After listening to each musical piece, you will be asked to fill out another questionnaire. The questionnaire will ask you to rate what you listened to. For some of you the infrasound will be on, for others it will be off. Right now the speaker is off. We can’t tell you which condition you will be in right now, but we’ll let you know after the study is over. While we debrief you on the study, you will provide another saliva sample Is everything that I’ve said clear so far?

*[Wait for response]*

E: Good. I will now give a brief demonstration of how the saliva sample should be provided. [Demonstrate with vial and straw; don’t actually rinse/spit]. First, you will pour some water into the paper cup provided. You will swish the water in your mouth and swallow 3 times for at least 5 seconds each time to ensure your mouth has been properly rinsed. After your mouth has been rinsed, you will hold the tube at your desk and spit through the sterile straw provided into the tube. Please ensure that you have filled the tube to approximately the black line shown on the tube. After your sample has been collected, please seal the tube with the cap next to it and place it back in the position on the desk where you picked it up from. This will be collected at the end of the experiment. A cheat sheet will be provided near the sample kit that you can follow. Are these instructions clear?

*[Wait for response]*

E: Good. Before we begin I’d like you to read the consent and credit forms that are on your desk. Carefully read the consent form and sign it if you decide to participate in this study. Your participation is completely voluntary and you are free to withdraw at any time without the loss of credit. You can now move your chairs to the desks and complete the forms. Once you’re finished please bring your chairs and your forms back to the doorway and we’ll begin.

*[Wait until they appear to be finished]*

E: Are you finished?

*[Wait for response, then pick up the consent and credit forms. Check the consent form for signatures. If one of the participants does not consent, go directly into the verbal debriefing. If they both consent, say, “I will be right back”. Bring the signed consent forms to 6-340A. Move back to the center of the room.]*

E: Are you ready to begin? [*Wait for response*]

E: Follow the instructions on your computers. Each stage of the study is time-locked so take your time because you cannot move on until the timer runs out. You will have lots of time to finish each stage. Okay, you can now turn to your computers and wait for me to tell you “you may now begin” to start the first questionnaire and then follow the rest of the instructions on the screen. *[Wait for response and for the participants to turn to their screens]*

E: You may now begin.

*[On the infrasound speaker in the main room, there will be a notepad. Take the top page off the notepad to reveal which condition the participant is in. In the infrasound condition, turn the amplitude up to the mark on the frequency generator* *]*

[*Move into 6-340A and wait until participants are finished*]

[*Move back into the middle of the room so that both participants can see you*]

E: Are you both finished?

*[Wait for response]*

E: Good. Once we have reached 20 minutes after the onset of the music clip, I will ask you to provide one more saliva sample before you leave.

*[Ensure that participants stay until 15-20 min after music started. If you are already at that point, simply ask them to collect. If it has not yet been 15-20 min, ask them to stay seated until 15 min and then provide another sample]*

E: Are you finished giving your second saliva sample?

*[Wait for response]*

E: Good, thank you. I will now debrief you on the study you just participated in.

E: *[verbal debrief]* Did you think the infrasound was on or off? [Wait for response] Well, it was ***[ON/OFF]***. This experiment examined the effect of infrasound on your perception of the videos you watched. We expected that exposure to infrasound may change your perception of the music. Some theories of the impact of infrasound predict that you would find the music less pleasant when the infrasound was on. Other theories predict that you would like pleasant music more and unpleasant music less when the infrasound is on. In this study, you were exposed to clips of both calming music and unsettling music. We were interested if the infrasound changed how you evaluated these clips.

E: Do you have any questions?

*[Wait for response]*

Thank you again for your participation, we greatly appreciate it.

*[Once you have finished the verbal debriefing, hand out the hard copy of the general debriefing form, explain that participants can keep that copy.]*

***[After the participant leaves, access the locked portion of the survey to mark their condition.]***

***[At the end of the testing day, please bring the marked saliva samples to the assigned freezer in the biology lab (if Kale) or to the behavioural neruoscience lab fridge (if not Kale) and email Kale Scatterty that there are samples ready for itemization and long term storage]***

**Cheat Sheet (Provided to Participants)**


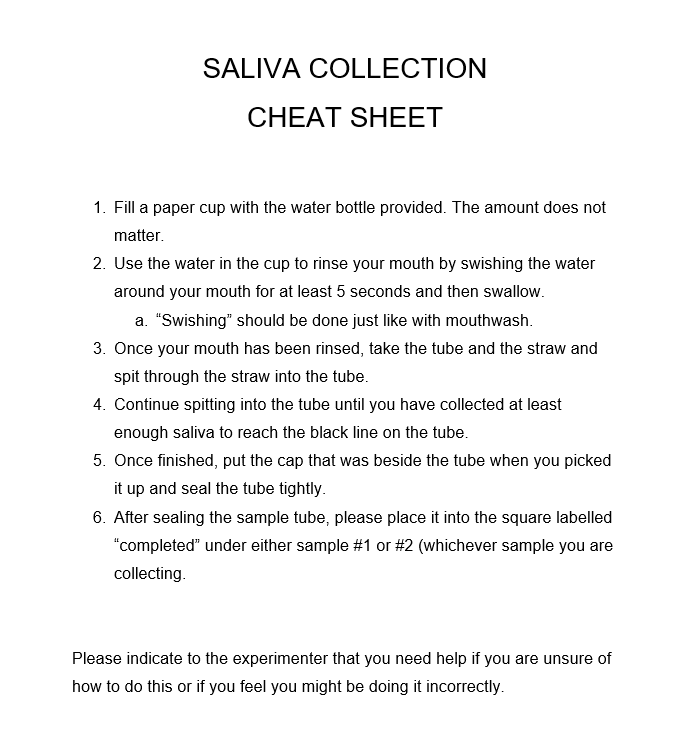


**Saliva Sample Placement Sheet**

**
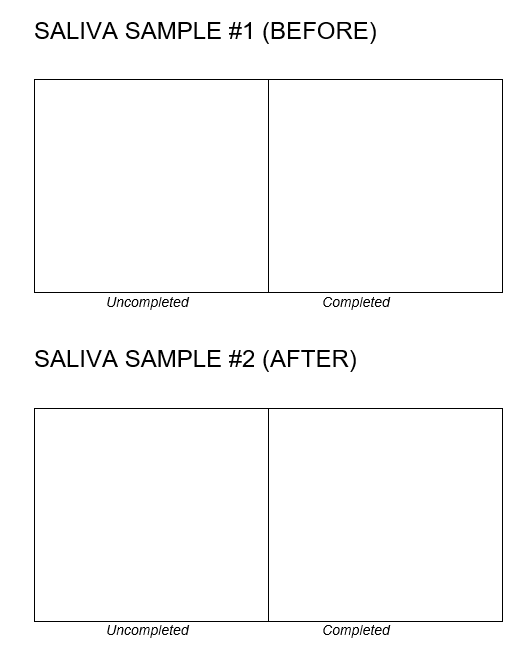
**

**Plate Reader Layout and Readouts**

**
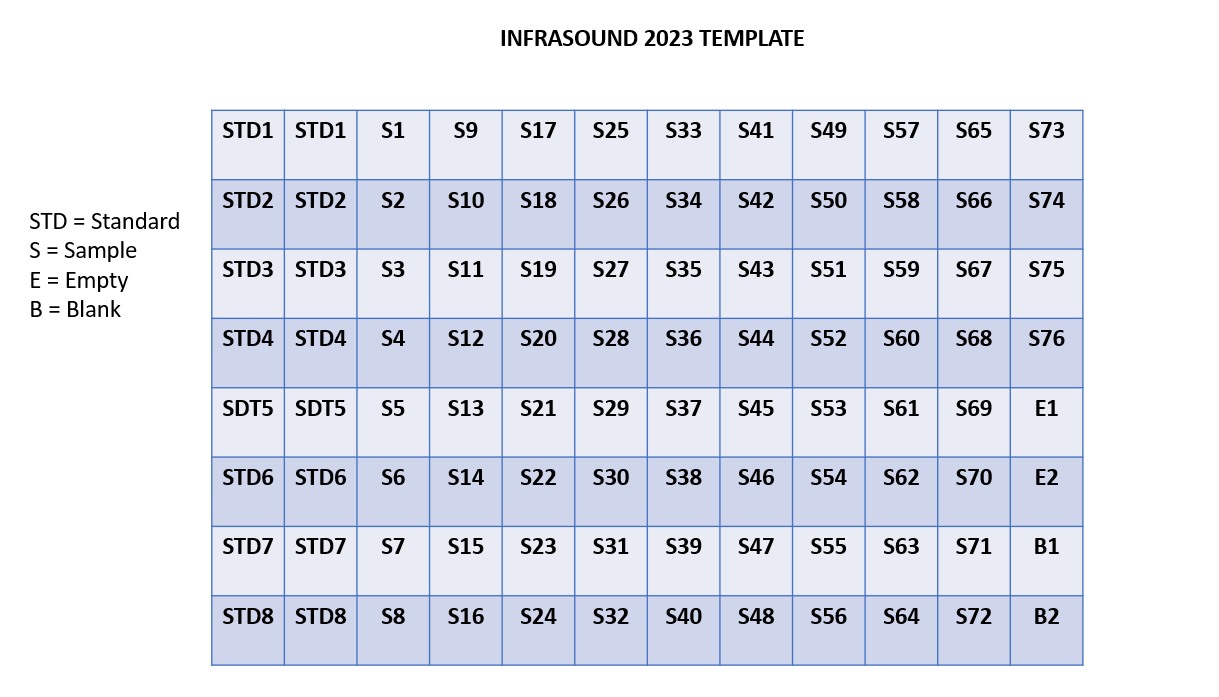
**


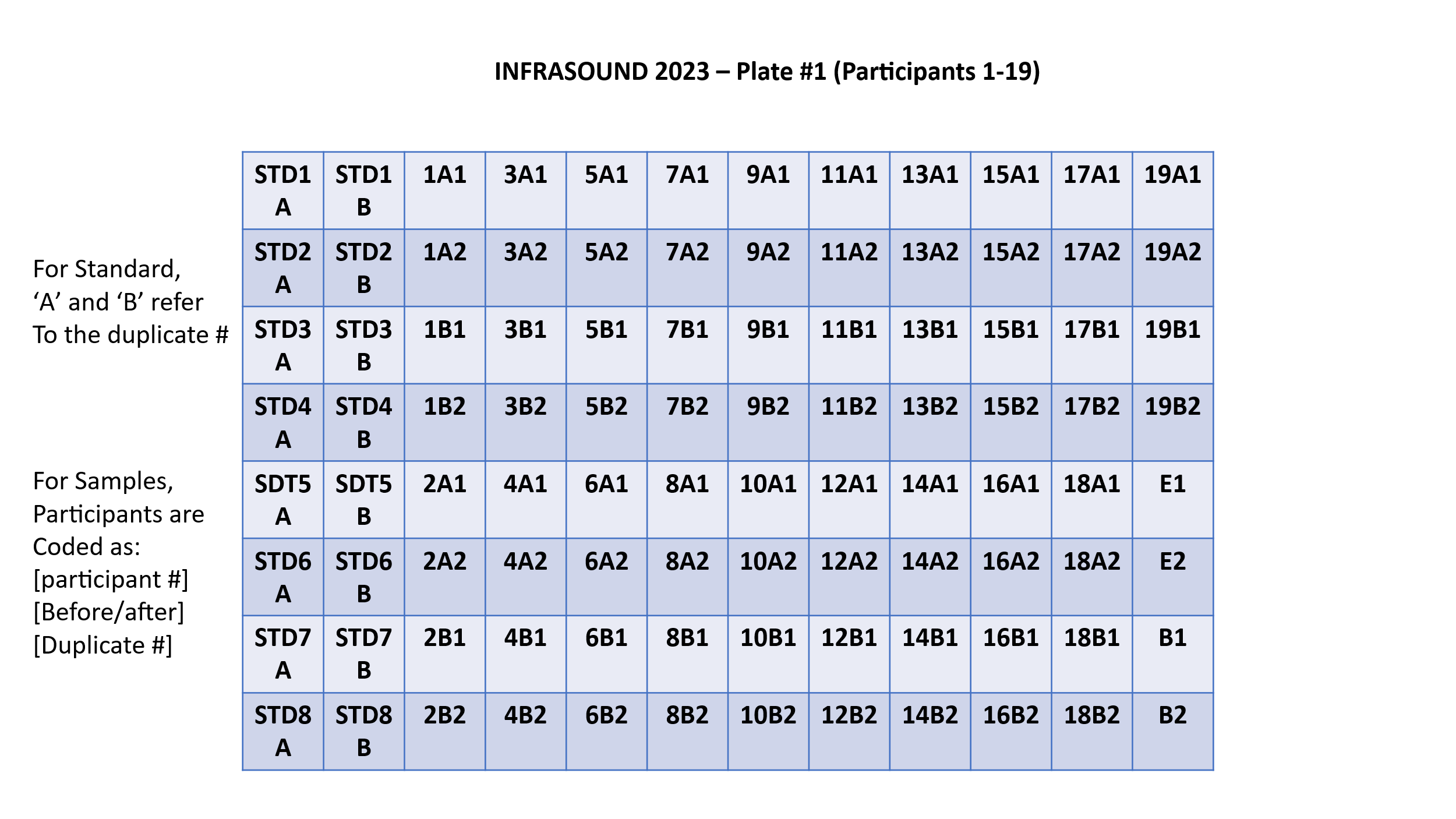


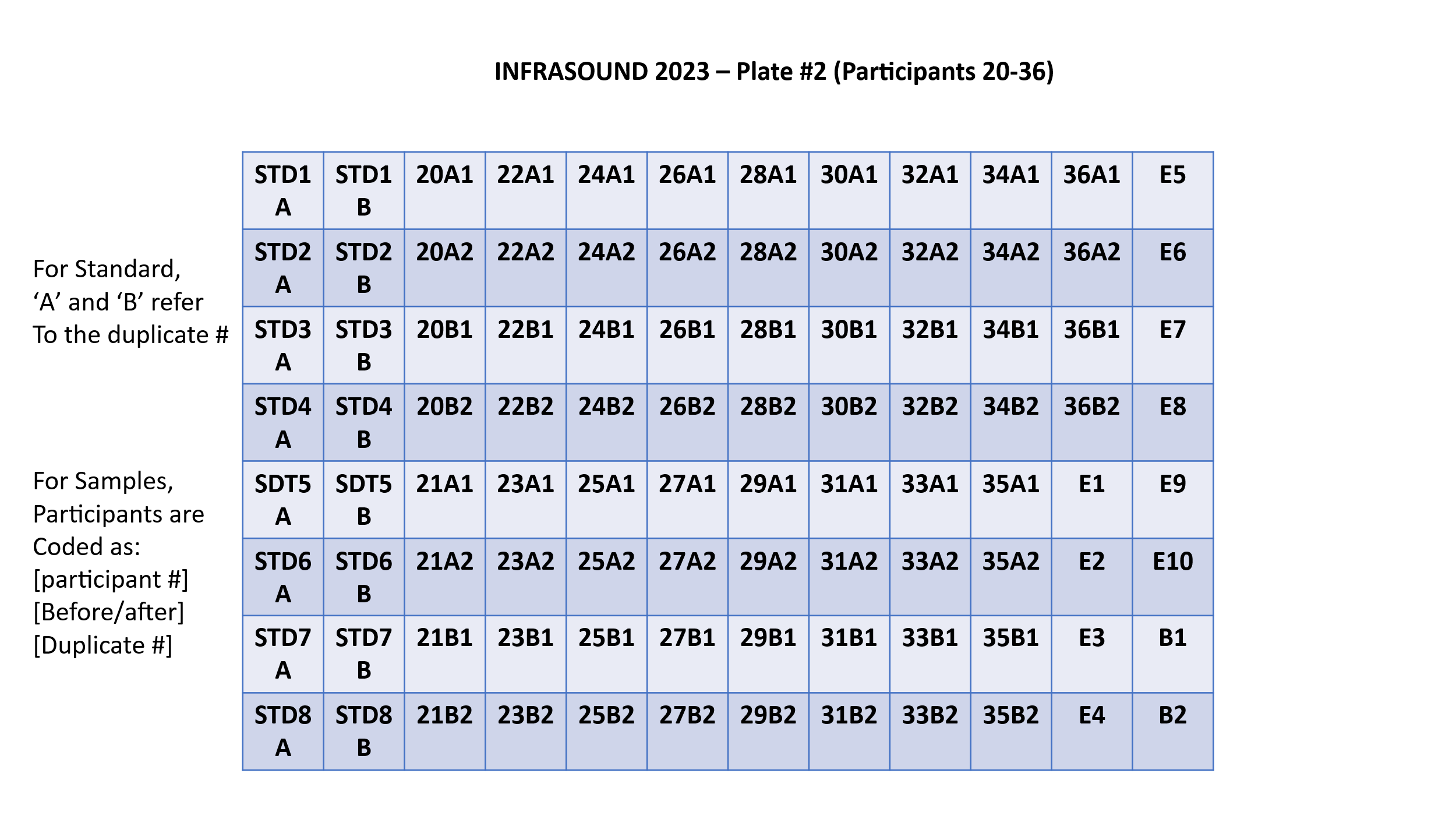

**R Code for Conditional Inference Forest Model**

## Required Packages ####

# Only load these if they aren't installed

install.packages("caTools")

install.packages("stablelearner")

### Decision Tree/Random Forest Packages ####

install.packages("tree")

### Conditional Inference Tree/Forest Packages ####

install.packages("party")

install.packages("Hmisc")

install.packages("pdp")

## Load Packages ####

library(readxl)

library(partykit)

library(party)

library(Hmisc)

library(pdp)

library(tree)

library(randomForest)

library(reprtree)

library(caTools)

# Processing ####

## Load Data Set ####

InfrasoundHumans2023DataR <- read_excel("InfrasoundHumans2023DataR.xlsx")

View(InfrasoundHumans2023DataR)

# str(InfrasoundHumans2023DataR)

ss.data <- InfrasoundHumans2023DataR[-12,-c(1:6,54,56,57,59)]

View(ss.data)

## Full Data Set ####

ss.data$Song <- as.factor(ss.data$Song)

ss.data$Infrasound <- as.factor(ss.data$Infrasound)

# Data Analysis ####

## Decision tree ####

### Full ####

tree.try <- tree(Infrasound ~ ., data = ss.data)

summary(tree.try)

plot(tree.try)

text(tree.try, pretty = 0)

tree.try

### Training/Test Split ####

( n <- nrow(ss.data) )

class.rate <- NULL

percent.split <- 0.75

for(i in 1:500){

train <- sample(1:n,percent.split*n)

test.true <- ss.data$Infrasound[-train]

tree.infra <- tree(Infrasound~., ss.data, subset = train )

tree.pred <- predict(tree.infra, test.set, type = "class")

tab.class <- table(tree.pred,test.true)

class.rate[i] <- sum(diag(tab.class))/sum(tab.class)

}

summary(class.rate); sd(class.rate)

class.rate <- NULL

percent.split <- 0.75

for(i in 1:500){

# cat("Trial =",i,"\n")

train <- sample(1:n,percent.split*n)

test.set <- ss.data[-train,]

test.true <- ss.data$Infrasound[-train]

tree.infra <- tree(Infrasound~., ss.data, subset = train )

cv.infra <- cv.tree(tree.infra, FUN = prune.misclass)

# par(mfrow = c(1,2))

# plot(cv.infra$size, cv.infra$dev, type = "b")

# plot(cv.infra$k, cv.infra$dev, type = "b")

# par(mfrow=c(1,1))

best <- cv.infra$size[which(cv.infra$dev == min(cv.infra$dev))]

# cat("best = ",best,"\n")

# cat("length best =",length(best),"\n")

if(length(best) > 1){

# print("Yes")

best <- best[1]

}

# cat("best =",best,"\n")

prune.infra <- prune.misclass(tree.infra, best = best)

if(nrow(prune.infra[[1]]) > 1){

# plot(prune.carseats)

# text(prune.carseats, pretty = 0)

tree.pred <- predict(prune.infra, test.set, type = "class")

tab.class <- table(tree.pred,test.true)

# print(tab.class)

# print(sum(diag(tab.class))/sum(tab.class))

class.rate[i] <- sum(diag(tab.class))/sum(tab.class)

}

}

summary(class.rate); sd(class.rate,na.rm=TRUE)

## Bagging ####

p <- ncol(ss.data)

bag <- randomForest(Infrasound ~ ., mtry = p-1, data = ss.data, importance = TRUE)

class.rate <- NULL

percent.split <- 0.75

## Random Forest ####

p <- ncol(ss.data)

rf <- randomForest(Infrasound ~ ., mtry = floor(p/2), data = ss.data)

importance(rf)

# coerce to a stabletree

srf <- stablelearner::as.stabletree(rf)

print(srf)

summary(srf, original = FALSE) # there is no original tree

barplot(srf)

image(srf)

plot(srf)

### Training/Test Split ####

( n <- nrow(ss.data) )

class.rate <- NULL

percent.split <- 0.75

trials <- 500

imp.mat <- matrix(NA,nrow=p-1,ncol=trials)

for(i in 1:trials){

cat("Trial =",i,"\n")

train <- sample(1:n,percent.split*n)

test.set <- ss.data[-train,]

test.true <- ss.data$Infrasound[-train]

rf.infra <- randomForest(Infrasound~., mtry = floor(p/2), ss.data, subset = train )

imp.mat[,i] <- importance(rf.infra)

# print(head(importance(rf.infra)))

rf.pred <- predict(rf.infra, test.set, type = "class")

tab.class <- table(rf.pred,test.true)

class.rate[i] <- sum(diag(tab.class))/sum(tab.class)

}

summary(class.rate); sd(class.rate)

rownames(imp.mat) <- colnames(ss.data[,-48])

res.imp <- cbind(

apply(imp.mat,1,mean),

apply(imp.mat,1,sd)

)

colnames(res.imp) <- c("mean","sd")

res.imp[order(res.imp[,1],decreasing=TRUE),]

## Conditional Inference Tree ####

cit.infra <- ctree(Infrasound ~ ., data = ss.data)

plot(cit.infra)

nodes(cit.infra,1)[[1]]$criterion$criterion

plot(cit.infra, inner_panel = node_barplot)

nodes(cit.infra, 1)[[1]]$prediction

## Conditional Random Forest ####

p <- ncol(ss.data)

set.seed(61)

crf.infra <- cforest(Infrasound ~., data = ss.data,

controls=cforest_unbiased(mtry = floor(p/2), ntree = 500))

infra.imp <- varimp(crf.infra, conditional = TRUE)

dotchart(sort(infra.imp), xlab = "Conditional variable importance")

abline(v = abs(min(infra.imp)), lty = 2, lwd = 2, col = "red")

cforest_model <- partykit::cforest(Infrasound ~ ., data = ss.data, ntree = 500)

cf_partykit_st <- stablelearner::as.stabletree(cforest_model)

summary(cf_partykit_st, original = FALSE)

### Repetition for variance ####

p <- ncol(ss.data)

trials <- 25

class.rate <- NULL

C <- NULL

mtry.val <- sqrt(p) #Can try sqrt(p) or p/2

imp.mat <- matrix(NA,nrow=p-1,ncol=trials)

rownames(imp.mat) <- colnames(ss.data[,-48])

for(i in 1:trials){

cat("Trial =",i,"\n")

crf.infra <- cforest(Infrasound ~., data = ss.data,

controls=cforest_unbiased(mtry = floor(mtry.val), ntree = 500))

imp.mat[,i] <- varimp(crf.infra, conditional = TRUE)

pred.crf.oob <- predict(crf.infra, OOB = TRUE)

tab.class <- table(pred.crf.oob, ss.data$Infrasound)

class.rate[i] <- sum(diag(tab.class))/sum(tab.class)

prob.crf.oob <- unlist(predict(crf.infra, type="prob", OOB=TRUE))[c(FALSE, TRUE)]

C[i] <- somers2(prob.crf.oob, as.numeric(ss.data$Infrasound)- 1)[1]

}

res.imp <- cbind(

apply(imp.mat,1,mean,na.rm=T),

apply(imp.mat,1,sd,na.rm=T)

)

colnames(res.imp) <- c("mean","sd")

res.imp[order(res.imp[,1],decreasing=TRUE),]

dotchart(sort(res.imp[,1]), xlab = "Average conditional variable importance", main="Variable Importance Plot")

abline(v = abs(min(res.imp[,1])), lty = 2, lwd = 2, col = "red")

# Out of bag classification rate

summary(class.rate); sd(class.rate)

# C-index It shows the proportion of times when the randomly sampled observation

#with outcome A also has a higher probability of A predicted by the model than a

# randomly sampled instance of B.

summary(C); sd(C)

### Training/Test Split ####

n <- nrow(ss.data)

p <- ncol(ss.data)

class.rate <- NULL

percent.split <- 0.9

trials <- 25

mtry.val <- sqrt(p) #Can try sqrt(p) or p/2

imp.mat <- matrix(NA,nrow=p-1,ncol=trials)

for(i in 1:trials){

cat("Trial =",i,"\n")

train <- sample(1:n,percent.split*n)

train.set <- ss.data[train,]

test.set <- ss.data[-train,]

test.true <- ss.data$Infrasound[-train]

crf.infra <- cforest(Infrasound ~., data = train.set,

controls=cforest_unbiased(mtry = floor(mtry.val), ntree = 500))

imp.mat[,i] <- varimp(crf.infra, conditional = TRUE)

# print(head(importance(rf.infra)))

crf.pred <- predict(crf.infra, newdata=test.set)

tab.class <- table(crf.pred,test.true)

class.rate[i] <- sum(diag(tab.class))/sum(tab.class)

}

# Model performance

summary(class.rate); sd(class.rate)

# Importance Score evaluation

rownames(imp.mat) <- colnames(ss.data[,-48])

res.imp <- cbind(

apply(imp.mat,1,mean),

apply(imp.mat,1,sd)

)

colnames(res.imp) <- c("mean","sd")

res.imp[order(res.imp[,1],decreasing=TRUE),]

dotchart(sort(res.imp[,1]), xlab = "Conditional variable importance", main="Training Data Sets")

par(marabline(v = abs(min(res.imp[,1])), lty = 2, lwd = 2, col = "red"))
